# In-Water and Neat Batch and Continuous-Flow Direct Esterification and Transesterification by a Porous Polymeric Acid Catalyst

**DOI:** 10.1038/srep25925

**Published:** 2016-05-18

**Authors:** Heeyoel Baek, Maki Minakawa, Yoichi M. A. Yamada, Jin Wook Han, Yasuhiro Uozumi

**Affiliations:** 1 RIKEN Center for Sustainable Resource Science, Wako, Saitama 351-0198, Japan; 2 Department of Chemistry, Hanyang University, Seoul 04763, Korea; 3 Institute for Molecular Science (IMS), Myodaiji, Okazaki, Aichi 444-8787, Japan

## Abstract

A porous phenolsulphonic acid—formaldehyde resin (PAFR) was developed. The heterogeneous catalyst PAFR was applied to the esterification of carboxylic acids and alcohols, affording the carboxylic acid esters in a yield of up to 95% where water was not removed from the reaction mixture. Surprisingly, the esterification in water as a solvent proceeded to afford the desired esters in high yield. PAFR provided the corresponding esters in higher yield than other homogeneous and heterogeneous catalysts. The transesterification of alcohols and esters was also investigated by using PAFR, giving the corresponding esters. PAFR was applied to the batch-wise and continuous-flow production of biodiesel fuel FAME. The PAFR-packed flow reactor that was developed for the synthesis of carboxylic acids and FAME worked for four days without loss of its catalytic activity.

Dehydrative esterification of carboxylic acids and alcohols, also known as Fischer esterification^1^, and transesterification of esters with alcohols^2^,^3^,^4^,^5^,^6^,^7^ are important fundamental reactions for obtaining organic esters. These are both equilibrium reactions: the esterification of carboxylic acids and alcohols gives the carboxylic acid esters and water; the transesterification of organic esters with alcohols affords the corresponding organic esters and released alcohols. Since these reactions are thermodynamically reversible, their completion requires removal of the resulting water and alcohols.

Nonequilibration of these equilibrium reactions should overcome the issue. For example, some enzymes mediate the dehydrative reaction in water for the synthesis of nucleic acids and proteins with the assistance of phase separation^8^. Some homogeneous/heterogeneous catalysts promoted in-water esterification by tuning their hydrophobicity or hydrophilicity as well as phase separation ability^9^,^10^,^11^,^12^,^13^,^14^,^15^,^16^,^17^,^18^,^19^,^20^,^21^. Some homogeneous/heterogeneous catalysts promoted it under neat or solvent conditions^9^,^10^,^11^,^12^,^13^,^14^,^15^,^16^,^17^,^18^,^19^,^20^,^21^,^22^,^23^,^24^,^25^,^26^,^27^. For example, Kobayashi *et al.* reported esterification under neat conditions by using a surfactant-type dodecylbenzene sulfonic acid catalyst (DBSA). Yamamoto and Ishihara *et al.* reported highly efficient and direct ester condensation in hydrocarbons by using hafnium(IV) salts such as an HfCl_4_·(THF)_2_ catalyst under azeotropic reflux conditions. Transesterification of various esters with alcohols using heterogeneous and homogeneous acidic catalysts has also been reported^28^,^29^,^30^,^31^,^32^,^33^,^34^,^35^,^36^,^37^,^38^.

We designed a phenolsulfonic acid – formaldehyde resin (PAFR) catalyst as a novel acidic insoluble catalyst for the esterification of carboxylic acids and alcohols. Although PAFRs have been utilized as electron-conductive compositions or cation exchangers^39^,^40^,^41^,^42^,^43^,^44^,^45^, they have not been used for heterogeneous catalysts. During our investigation of their preparation procedure, we found that macroporous and nonporous PAFR (**1a** and **1b**, respectively (Fig. 1)) were prepared separately by changing the cooling time. To our surprise, 0.7 mol% of the macroporous PAFR **1a** promoted the esterification of carboxylic acids and alcohols under neat conditions without removal of water more efficiently than the nonporous PAFR **1b** and general homogeneous/heterogeneous acid catalysts, affording the corresponding esters with up to a quantitative yield. Here, we show the full details of the preparation of PAFR **1a** and its application to the esterification of carboxylic acids and alcohols under neat conditions without removal of water^46^. At the time, we were pleased to find that the in-water esterification proceeded smoothly by using **1a** to give the corresponding esters with high yield. Catalyst **1a** was also applied to the transesterification of carboxylic esters with alcohols. Moreover, a column packed with PAFR was utilized for the continuous-flow esterification to give the corresponding esters including the biodiesel fuel FAME.

## Results and Discussion

### Preparation of Phenolsulfonic Acid–Formaldehyde Resins (PAFRs) 1a and 1b

**Figure 1:**
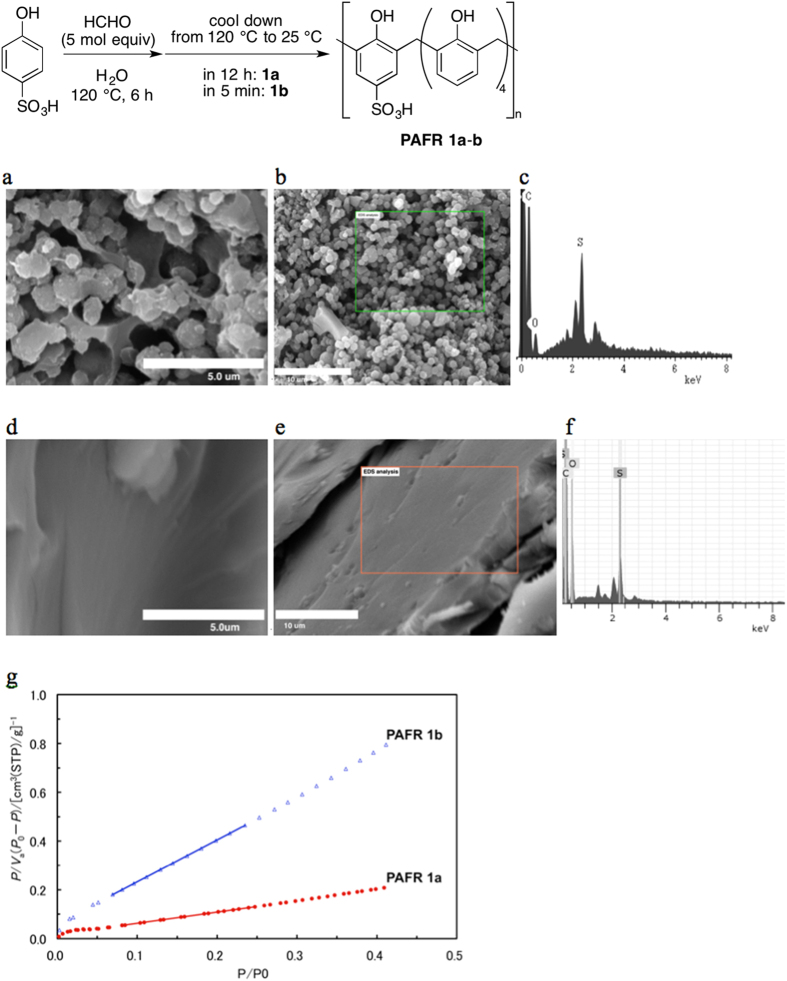
Preparation and images of catalysts PAFR **1a** and **1b**; PAFR **1a** images of (**a**) SEM (x15k), (**b**) SEM (x5k), (**c**) EDS; PAFR **1b** images of (**d**) SEM (x15k), (**e**) SEM (x5k), (**f**) EDS; (**g**) BET of PAFRs **1a** and **1b**.

PAFRs **1a** and **1b** were prepared by polymerization of *p*-phenolsulfonic acid with formaldehyde in H_2_O to give brown precipitates. The suspension was cooled from 120 °C to 25 °C in 12 h, and dried under reduced pressure, affording brownish and insoluble PAFR **1a**. High-resolution Scanning Electron Microscope (SEM) observation revealed that PAFR **1a** was an aggregated macroporous solid with a pore size of 1–5 μm wide, in which sulfur moiety from the SO_3_H unit in **1a** was readily detected (Energy Dispersive x-ray Spectroscopy, EDS) (Fig. 1(a–c)). Infrared spectra and elemental analysis (Figure S1, supporting information) of PAFR **1a** showed the ratio of phenol sulfonic acid moiety/phenol moiety as 1/4. In contrast, when the reaction mixture was suddenly cooled down to 25 °C in 5 min, a hardly soluble brownish solid was also given. However, high- and low-resolution SEM did not show a macroporous structure in **1b** although sulfur atoms (SO_3_H moiety) were uniformly dispersed on the polymeric matrix (Fig. 1(d–f)). The surface area of **1a** and **1b** was 11.5 m^2^/g and 3.1 m^2^/g, respectively (Kr-adsorption Brunauer–Emmett–Teller (BET) analysis; Fig. 1(g)). We have not clarified the reason for the formation of macropores in **1a**. However, water could have been absorbed in **1a** during gradual cooling, acting as a template for macropore formation. Evaporation of water under reduced pressure could have provided the macropores in **1a**. In contrast, water might have been discharged from **1b** during sudden cooling, giving the flat surface on **1b**.

### Direct Esterification and Transesterification without Removal of the Resulting Water/Alcohol

**Figure 2:**
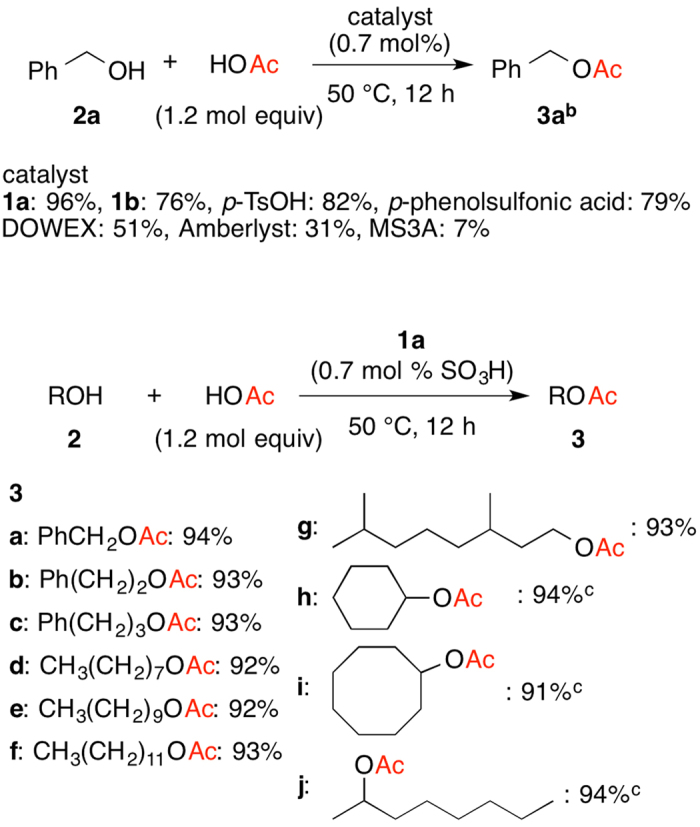
Direct Esterification of Benzyl Alcohol (**2a**) and Acetic acid Catalyzed by Various Catalysts, and PAFR **1a**–Promoted Esterification of **2a–h** and Acetic Acid^a^. (**a**) Conditions: **2a** (1.0 mmol), acetic acid (1.2 mmol), **1a** (0.0070 mmol); (**b**) The yield % was determined with GC analysis; (**c**) 80 °C.

With PAFRs **1a** and **1b** in hand, the reaction of benzyl alcohol (**2a**) with acetic acid was carried out with less than 0.1 mol% of homogeneous and heterogeneous catalyst including **1a** and **1b** to survey the catalytic activity in the esterification (Fig. 2). In all the cases, the resulting water in the reaction was not removed from the reaction suspension/solution during the reaction. The reaction with PAFR **1a** provided higher conversion to give benzyl acetate (**3a**) in 96% while that in PAFR **1b** was slower to afford **3a** in 76%. The esterification with homogeneous catalysts *p*-toluenesulfonic acid (*p*-TsOH) and *p*-phenolsulfonic acids under similar conditions yielded **3a** in 82% and 79%, respectively. Heterogeneous catalyst (DOWEX, Amberlyst and MS3A) led to the formation of **3a** in 51%, 31%, and 7%, respectively. Since PAFR **1a** has hydrophilic pore structure in the catalyst, it could absorb hydrophilic alcohols and carboxylic acids and convert them to the less hydrophilic esters. The product might be discharged from the catalyst, and not attached to the catalyst. This is our working hypothesis of the efficient esterification with phase separation under nonequilibrium conditions to give the desired esters.

Since **1a** was the best catalyst for direct esterification, the reaction of various alcohols with acetic acid was carried out at 50 °C for 12 h by using **1a** (0.7 mol%) (Fig. 2). All the products in the following esterification were isolated to determine the isolated yield. The reaction of benzyl alcohol (**2a**) gave **3a** in 94% isolated yield (Entry 1) that corresponded with GC yield (96%). The reaction of aliphatic primary alcohols **2b**–**g** proceeded smoothly to give the corresponding esters **3b**–**g** in 92–93% (Entries 2–7). The secondary alcohols **2 h**–**j** was converted at 80 °C to the esters **3 h**–**j** in up to 94% (Entries 8–10).

**Figure 3:**
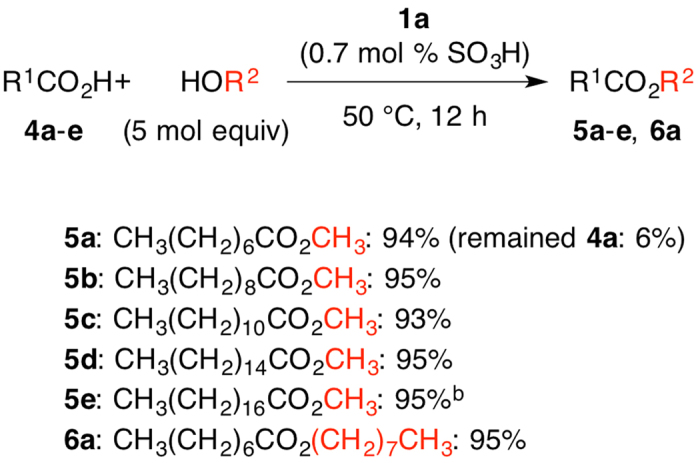
PAFR **1a**–Promoted Esterification of **4a–e**^a^. (**a**) Conditions: **4a**–**e** (1.0 mmol), alcohol (5.0 mmol), **1a** (0.0070 mmol); (**b**) 60 °C.

Figure 3 shows the esterification of carboxylic acids **4a**–**e** with methyl alcohol and octanol. The reaction of a variety of carboxylic acid was carried out with methyl alcohol to afford the esters **5a**–**e** in 93–95% (Entries 1–5). The reaction of **4a** with octanol was performed to yield **6a** in 95% (Entry 6).

**Figure 4:**
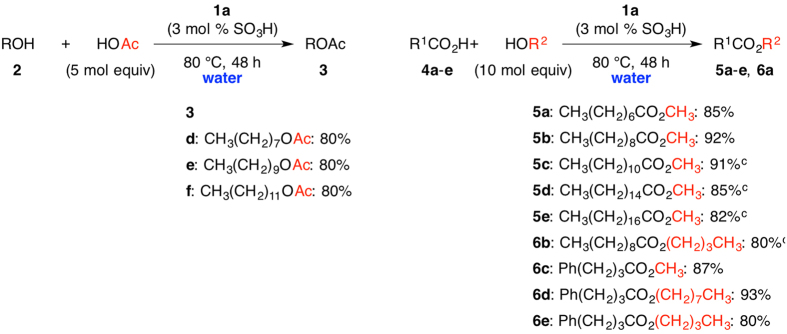
In-Water Esterification of Alcohols **2d–f** and AcOH^a^, and In-Water Esterification of Carboxylic Acids and Alcohols^b^. (**a**) Conditions: **2** (1.0 mmol), acetic acid (5.0 mmol), **1a** (0.03 mmol), water (3.0 M substrate); (**b**) Conditions: **4** (1.0 mmol), alcohol (10 mmol), **1a** (0.03 mmol), water (3.0 M substrate); (**c**) 90 °C.

Direct esterification in water is a kind of paradox reaction, and thus the development of heterogeneous acid catalysts is an important challenge in organic syntheses. Esterification of carboxylic acids and alcohols in water by homogeneous and heterogeneous catalysts has been reported although these catalytic system is still developing in terms of catalytic activity and loading^9^,^10^,^11^,^12^,^13^,^14^. Since catalyst **1a** efficiently promoted esterification under neat conditions, we tested the reaction in water, as shown in Fig. 4, left. The reaction of octanol (**2d**) with acetic acid was performed with 3 mol% of **1a** in water at 80 °C to give octylacetate (**3d**) with 80% yield (entry 1). The reaction of decanol (**2e**) and dodecanol (**2 f**) was also carried out under similar conditions to afford the corresponding acetates **3e** and **3 f** with 80% and 80% yield, respectively.

In-water direct esterification of various carboxylic acids and alcohols was investigated (Fig. 4, right). When the reaction of octanoic acid (**4a**) was carried out with methyl alcohol in water at 80 °C, we were pleased to find that the reaction proceeded to give methyl octanoate (**5a**) with 85% yield (entry 1). C10, C12, C16 and C18 carboxylic acids were readily converted: the reaction of decanoic acid (**4b**), dodecanoic acid (**4c**), hexadecanoic acid (**4d**), and octadecanoic acid (**4e**) with methyl alcohol gave the corresponding esters **5b**–**5e** with 92%, 91%, 85%, 82% yield, respectively (entries 2–5). Not only methyl alcohol but also ethyl alcohol, butyl alcohol and octanol were suitable reactants in this reaction: the reaction of decanoic acid (**4b**) and butyl alcohol in water gave butyl decanoate **6b** with 80% yield (entry 6). In-water esterification of hydrocinnamic acid with methyl alcohol, butyl alcohol and octanol was carried out to afford the corresponding esters **6c**–**e** with 80–93% yield (entries 7–9). As shown in the abovementioned results, the polymeric acid catalyst **1a** efficiently promoted in-water esterification of many carboxylic acids and alcohols.

**Figure 5:**
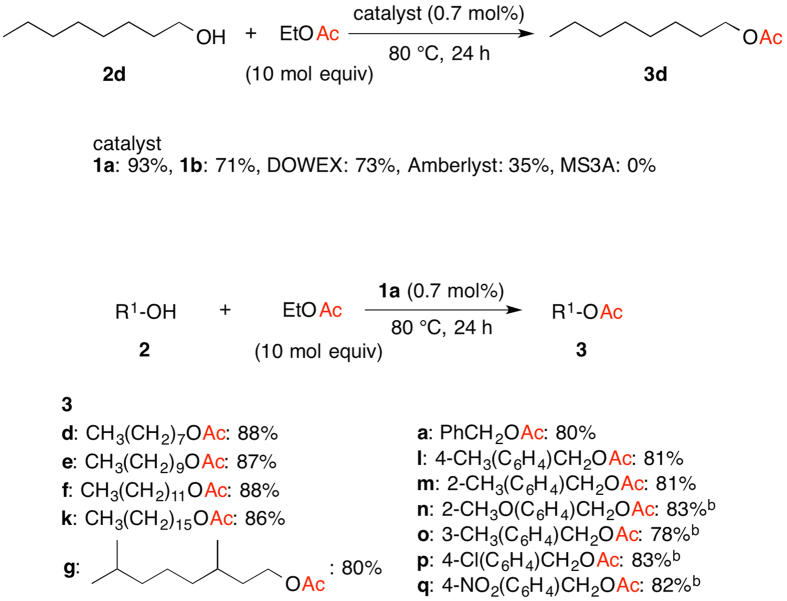
Transesterification of Octanol (**2d**) and Ethyl Acetate Catalyzed by Various Catalysts (top), and Transesterification of Alcohols **2** and Ethyl Acetate Catalyzed by PAFR **1a** (bottom)^a^. (**a**) Conditions: **2** (1.0 mmol), Ethyl Acetate (10 mmol), catalyst (0.007 mmmol); (**b**) 48 h.

Catalytic transesterification of esters with alcohols is an important transformation to produce valuable ester materials by simple exchange of alcohol moieties, and thus PAFR **1a** was applied to this transformation. The catalytic activity in transesterification was examined with less than 1 mol% of heterogeneous acid catalysts (Fig. 5, top). In all the cases, the resulting alcohol was not removed from the reaction vessel during the reaction. The reaction of octanol (**2d**) was performed with ethyl acetate and 0.7 mol% of **1a** at 80 °C for 24 h, giving a fruity-scented octyl acetate (**3d**) with 93% GC yield. In contrast, catalyst **1b** led to the formation of **3d** with 71% GC yield under similar conditions. The general heterogeneous catalysts Dowex, Amberlyst and Zeolite (MS3A) showed lower activity than that of **1a** to give **3d** with 73%, 35% and 0% GC yield, respectively.

Since **1a** had higher catalytic activity for not only direct esterification but also transesterification under neat conditions, the transesterification of various alcohols and ethyl acetate was investigated (Fig. 5, bottom). The products were isolated to determine the isolated yield. The reaction of octanol (**2d**) with ethyl acetate at 80 °C for 24 h gave **3d** with 88% isolated yield (entry 1). More hydrophobic aliphatic alcohols, decanol (**2e**), dodecanol (**2 f**) and hexadecanol (**2k**) were readily converted to the corresponding esters **3e**, **3 f** and **3k** with 87%, 88% and 86% yield, respectively (entries 2–4). Tetrahydrogeraniol (**2 g**) was reacted with ethyl acetate to give 3,7-dimethyloctyl acetate (**3 g**) with 80% yield (entry 5). Various benzylic alcohols were also suitable substrates for transesterification. The reaction of benzyl alcohol (**2a**) afforded benzyl acetate (**3a**) with 80% yield (entry 6). Benzylic alcohols bearing electron-donating and -withdrawing groups led to the formation of the corresponding esters with 78–83% yield (entries 6–12).

**Figure 6:**
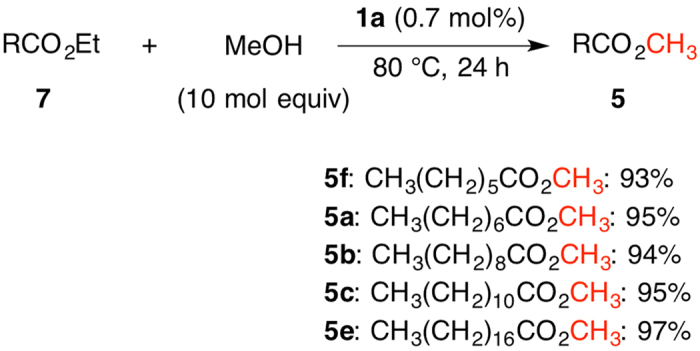
Transesterification of Ethyl Esters with Methyl Alcohol Catalyzed by **1a**^a^. (**a**) Conditions: Ethyl Esters **7** (1.0 mmol), Methyl Alcohol (10.0 mmol), **1a** (0.007 mmol).

Transesterification of carboxylic acid ethyl esters with methyl alcohol also proceeded to give the corresponding methyl esters (Fig. 6). Ethyl heptanoate (**7d**) was reacted with methyl alcohol at 80 °C for 24 h in the presence of **1a** to give methyl heptanoate (**5 f**) with 93% yield (entry 1). More hydrophobic carboxylic acid ethyl esters **7a**, **7b**, **7c** and **7e** were readily converted to the methyl esters **5a**, **5b**, **5c** and **5e** in 95%, 94%, 95% and 97% yield, respectively (entries 2–5).

**Figure 7:**
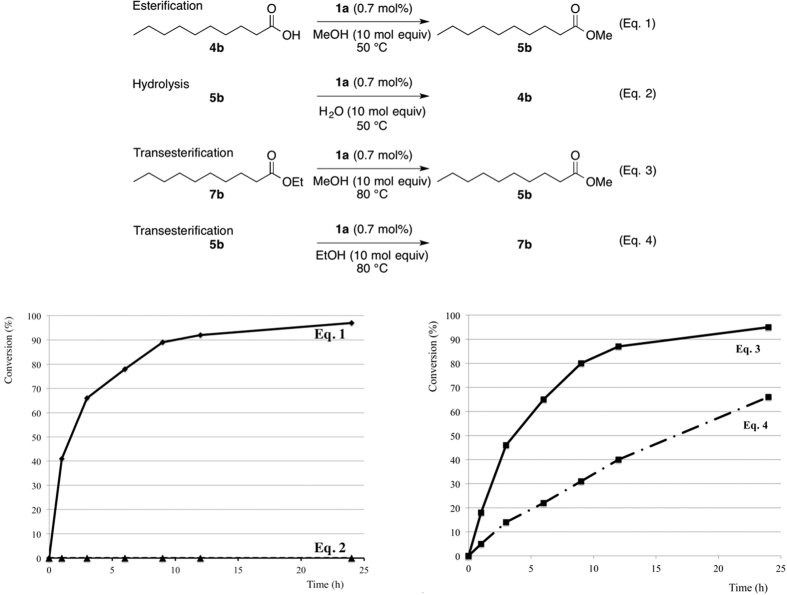
Time course experiments of esterification, hydrolysis and transesterification with PAFR **1a**.

To clarify the high conversion rate of esterification and transesterification using PAFR **1a**, time course experiments of the esterification of **4b** with methyl alcohol, the hydrolysis of **5b**, transesterification of **7b** and **5b** were investigated (Fig. 7). As shown in Eq. 1, direct esterification of **4b** with methyl alcohol proceeded smoothly at 50 °C to give the methyl ester **5b** with >90% conversion in 12 h. In contrast, the reverse reaction, the hydrolysis of **5b** with water did not proceed (Eq. 2). These results suggest that the equilibrium is not an important factor when using PAFR **1a**, and thus the high catalytic activity of **1a** is important for full conversion. Since the transesterification of the ethyl ester **7b** to the methyl ester **5b** was faster than that of the methyl ester **5b** to the ethyl ester **7b** (Eqs. 3 and 4), more that 90% yield of the methyl esters **5** in Fig. 6 should be obtained.

### Batch and Continuous-Flow Synthesis of Biodiesel Fuel (FAME) via Esterification and Flow Transesterification

**Figure 8:**
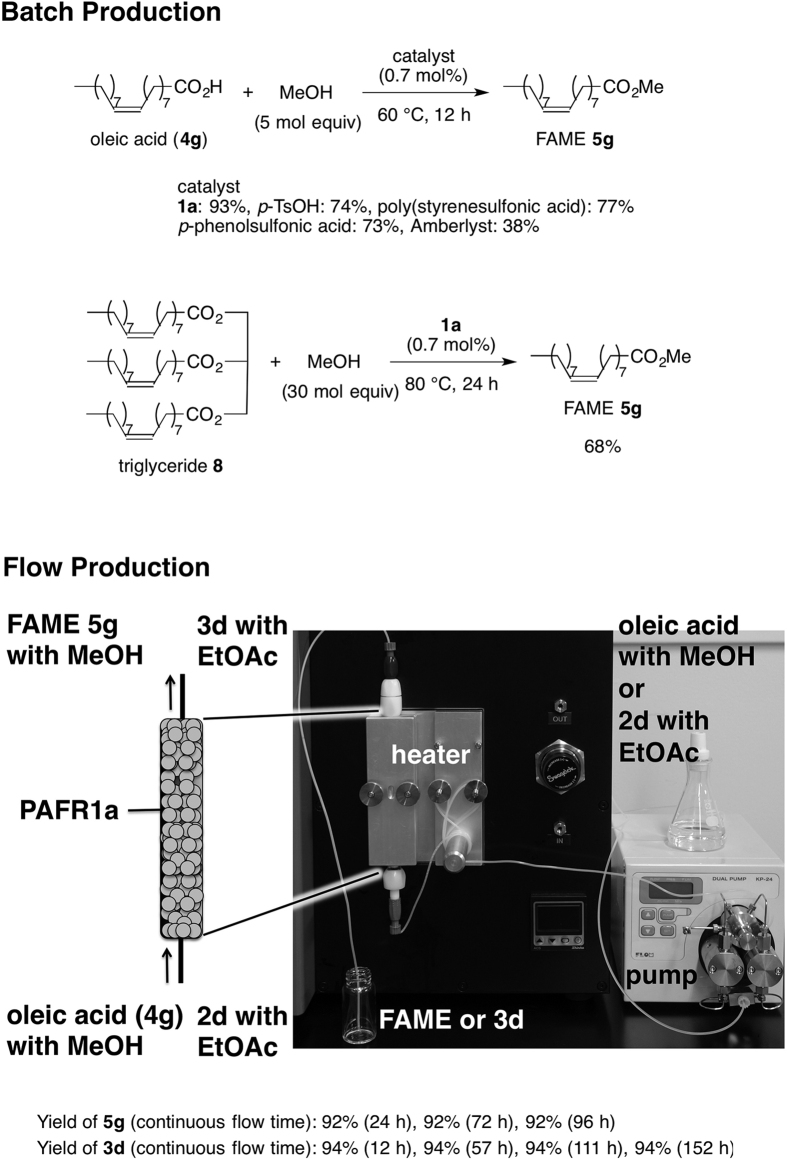
Batch and Flow Production of FAME **5 g** via Esterification, and of **3d** via Transesterification.

Efficient production of biodiesel fuel (fatty acid methyl ester; FAME) is important for green sustainable chemistry and fossil chemistry^47^,^48^,^49^,^50^. Researchers have reported esterification with solid acid catalysts for the production of biodiesel fuel^51^,^52^,^53^,^54^,^55^. Since the heterogeneous catalyst PAFR **1a** efficiently promoted direct esterification, it was applied to the synthesis of FAME **5 g** (Fig. 8, top). The reaction of oleic acid (**4 g**) with methyl alcohol was carried out with 0.7 mol% SO_3_H of **1a** to give **5 g** in 93%. In contrast, the reaction was performed with homogeneous and heterogeneous catalysts, *p*-TsOH, poly(styrenesulfonic acid), *p*-phenolsulfonic acid and Amberlyst under similar conditions, affording **5 g** in 74%, 77%, 73% and 38%, respectively. The results clearly show the superiority of PAFR **1a** for the efficient production of FAME. Transesterification of the triglyceride **8** was also carried out with PAFR **1a** to give FAME in 68%.

Moreover, the production of FAME **5 g** was achieved by using a column-type PAFR **1a**-promoted flow reactor under continuous-flow conditions. The flow reactor was composed of a PAFR **1a**-packed column with a heater and HPLC pump in which a methanolic solution of oleic acid (**4 g**) was continuously injected from an inlet (Fig. 8, bottom). The flow catalytic reaction system promoted the esterification in 18 min to provide FAME **5 g** in over 90% for continuous four days. Furthermore, the flow transesterification of **2d** with EtOAc also proceeded in 20 min to afford **3d** in 94% for continuous six days.

## Conclusion

PAFR **1a** was developed. The heterogeneous catalyst PAFR **1a** with less than 1 mol% promoted the esterification of carboxylic acids and alcohols, where the resulting water was not removed from the reaction vessel during the reaction. Direct esterification in water also proceeded by using PAFR **1a** to afford the corresponding esters. Transesterification was also performed with PAFR **1a**, efficiently giving the desired esters. Moreover, PAFR **1a** was applied to both batch and continuous-flow production of FAME, providing FAME **5 g** in high yield. We found that porosity in the polymeric acid catalyst was particularly important for efficient catalytic esterification and transesterification.

## Methods

### Preparation of a porous PAFR 1a and 1b

A mixture of a 2.0 M aqueous solution of *p*-phenol sulfonic acid (14.5 mL; 29.0 mmol), an 37% aqueous solution of formaldehyde (14.3 mL; 145 mmol) was stirred in a 300 mL flask with a reflux condenser at 120 °C (oil bath temperature) for 6 h under refluxing conditions. The flask was gradually cooled down to 25 °C in 12 h (for **1a**) or in 5 min (for **1b**) on an oil bath (for **1a**) or an ice-water bath (for **1b**) to give a pale brownish gel. The obtained gel material was washed with methanol and acetone, and then was dried under reduced pressure. A hardly soluble polymer PAFR **1a** was obtained in 72% yield (3.6 g) as a reddish brown solid. ATR-IR µ 3376, 1598, 1469, 1032, 773, 750, 708, 612 cm^−1^; Anal. Calcd. for (C_35_H_30_O_8_S·6 H_2_O)_n_: C, 58.49; H, 5.89; S, 4.46. Found: C, 57.78; H, 5.42; S, 4.08. **1b:** Anal. Calcd. for (C_35_H_30_O_8_S·2 H_2_O)_n_: C, 65.00; H, 5.30; S, 4.96. Found: C, 64.82; H, 5.67; S, 2.99.

### General procedure for the direct esterification under solvent-free conditions

To a 4 mL vial was added PAFR **1a** (0.7 mol%, 5.6 mg), an alcohol **2** (1.0 mmol), and acetic acid (1.2 mol equiv). The mixture was shaken by a shaker (16 Hz, Petisyzer) for 12 h at 50–80 °C. After the reaction, PAFR **1a** was filtered by filtration and washed with acetone. The filtrate was evaporated to give the corresponding acetate **3**.

### General procedure for the esterification in water

To a 4 mL vial was added PAFR **1a** (3 mol%, 24 mg), an alcohol **2** (1.0 mmol), and acetic acid (5 mol equiv) in water (3.0 M). The mixture was shaken by a shaker (16 Hz, Petisyzer) for 48 h at 80 °C. After the reaction, FAPR **1a** was filtered by filtration and washed with acetone. The filtrate was evaporated to give the corresponding acetate **3**.

### General procedure for the transesterification

To a 4 mL vial was added PAFR **1a** (0.7 mol%, 5.6 mg), **2** (1.0 mmol), and ethyl acetate (10 mol equiv). The mixture was shaken by a shaker (16 Hz, Petisyzer) for 24 h at 80 °C without removal of ethanol. After the reaction, PAFR **1a** was filtered by filtration and washed with acetone. The filtrate was evaporated to give the corresponding methyl esters **3**.

### Continuous flow reaction

PAFR **1a** (900 mg, 1.1 mmol) was packed into a glass column (15 cm × 6.6 mm bed reactor) that was attached to a heat block. A solution of the mixture of oleic acid and methanol was installed with a flow pump through capillary tubing. The flow reaction of oleic acid and methanol (5 mol equiv) was carried at a flow rate of 10 μL/min through the PAFR-packed column at 80 °C (residence time: 18 min). The reaction mixture solution was collected from the outlet of column, and evaporated to give FAME **5 g** in 92–96% conversion.

## Additional Information

**How to cite this article**: Baek, H. *et al.* In-Water and Neat Batch and Continuous-Flow Direct Esterification and Transesterification by a Porous Polymeric Acid Catalyst. *Sci. Rep.* **6**, 25925; doi: 10.1038/srep25925 (2016).

## Supplementary Material

Supplementary Information
