# Supplementary material for: In-Water and Neat Batch and Continuous-Flow Direct Esterification and Transesterification by a Porous Polymeric Acid Catalyst
Source: Sci Rep. 2016 May 18;6:25925. doi: 10.1038/srep25925 (PMC4870501; doi:10.1038/srep25925)
Supplement: Supplementary Information [file srep25925-s1.pdf]

SUPPORTING INFOMATION

In-Water and Neat Batch and Continuous-Flow Direct Esterification and Transesterification by a Porous Polymeric Acid Catalyst

Heeyoel Baek[1,3], Maki Minakawa[1], Yoichi M. A. Yamada[1,*], Jin Wook Han[3], and Yasuhiro Uozumi[1,2,*]

[1]RIKEN Center for Sustainable Resource Science, Wako, Saitama 351-0198, Japan
[2]Institute for Molecular Science (IMS), Myodaiji, Okazaki, Aichi 444-8787, Japan
[3]Department of Chemistry, Hanyang University, Seoul 04763, Korea
[*]ymayamada@riken.jp, uo@ims.ac.jp

General Information:

All reagents, purchased from TCI, Aldrich, Wako, MERCK, and Fluka, were used without further purification. Water was deionized with a Millipore system as a Milli-Q grade. NMR spectra were recorded with JEOL JNM-AL500 spectrometer (500 MHz) and JEOL JNM-AL400 spectrometer (400 MHz) in $CDCl_3$ at 25 °C. For ^1H NMR spectra, proton chemical shifts (δ) are given in ppm relative to tetramethylsilane (0.00 ppm) in $CDCl_3$. Multiplicities are indicated by s (singlet), d (doublet), t (triplet), m (multiplet), and br (broad). For ^{13}C NMR spectra, carbon chemical shifts were internally referenced to the deuterated solvent signal of $CDCl_3$ (77.16 ppm). Mass spectra were recorded with Accu TOF GC (JEOL JMS-100GC). SEM images were obtained by using a scanning electron microscope (Hitachi TS3030Plus). EDX (Energy dispersive X-ray spectroscopy) analyses were recorded on Bruker nanoGmbH Quantax 70. BET (Brunauer-Emmett-Teller) surface area analysis was performed on BELSORP-36 (BEL JAPAN, INC). ATR-IR spectra were taken with JASCO Fourier Transform Infrared Spectrometer-6200.

Amberlyst® 16 wet : CAS: 125004-35-5, H^+-form , strongly acidic (Fluka)

DOWEX: CAS: 69011-20-7, 50WX2-100-200 mesh (H) Cation Exchange Resin (Sigma-Aldrich)

MS3A: CAS: 308080-99-1, 1/16″ rod, 3 Å pore diameter (Wako)

p-phenolsulfonic acid: CAS:98-67-9, >85.0% (TCI)

p-TsOH: CAS: 6192-52-5 (Wako)

Experimental Section:

Preparation of a porous PAFR 1a and 1b

A mixture of a 2.0 M aqueous solution of *p*-phenol sulfonic acid (14.5 mL; 29.0 mmol), an 37 % aqueous solution of formaldehyde (14.3 mL; 145 mmol) was stirred in a 300 mL flask with a reflux condenser at 120 °C (oil bath temperature) for 6 h under refluxing conditions. The flask was gradually cooled down to 25 °C in 12 h (for **1a**) or in 5 min (for **1b**) on an oil bath (for **1a**) or an ice-water bath (for **1b**) to give a pale brownish gel. The obtained gel material was washed with methanol and acetone, and then was dried under reduced pressure. A hardly soluble polymer PAFR **1a** was obtained in 72% yield (3.6 g) as a reddish brown solid. ATR-IR ν 3376, 1598, 1469, 1032, 773, 750, 708, 612 cm^{-1}; Anal. Calcd. for $(C_{35}H_{30}O_8S{\bullet}6H_2O)_n$: C, 58.49; H, 5.89; S, 4.46. Found: C, 57.78; H, 5.42; S, 4.08. **1b:** Anal. Calcd. for $(C_{35}H_{30}O_8S{\bullet}2H_2O)_n$: C, 65.00; H, 5.30; S, 4.96. Found: C, 64.82; H, 5.67; S 2.99.

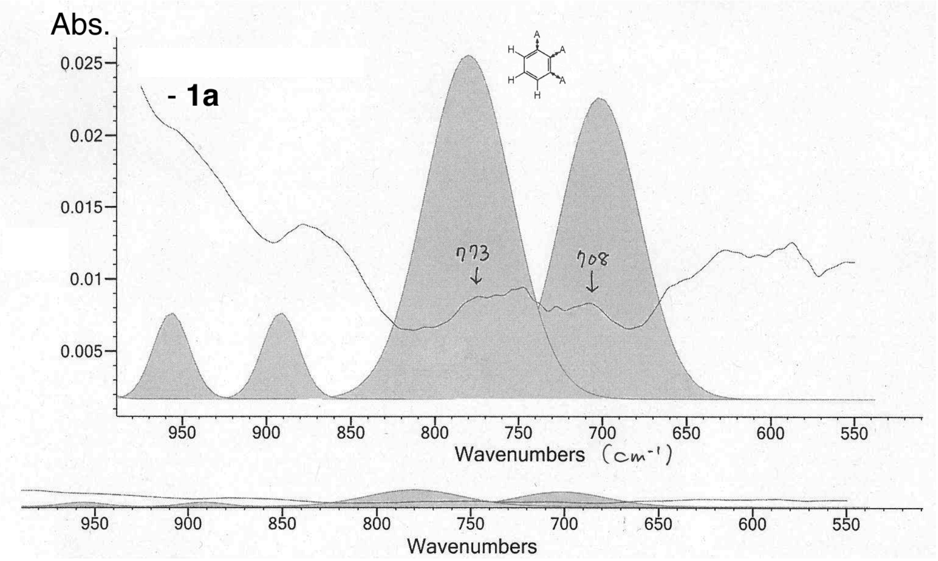

Figure S1. IR spectrum of **1a** and IR spectrum simulation of 1,2,3-trisubstituted benzenes (550-1000 cm^{-1})

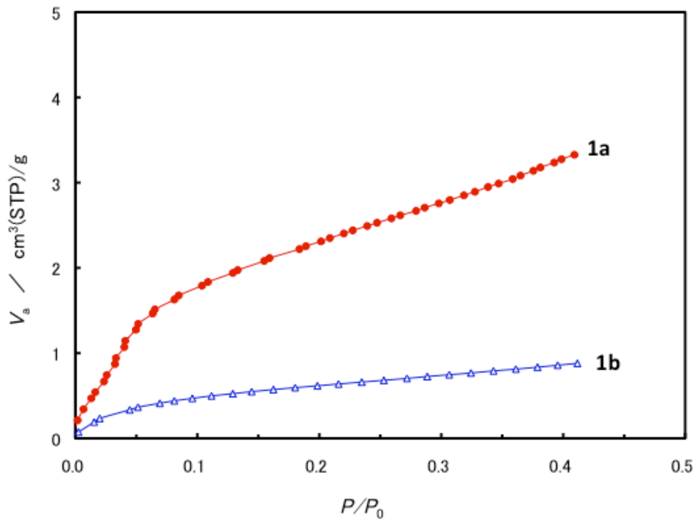

Figure S2. Kr-adsorption isotherm for **1a** and **1b**

General procedure for the direct esterification under solvent-free conditions

Figure 3:

To a 4 mL vial was added a *p*-phenol sulfonic acid-formaldehyde catalyst **1a**, **1b** (0.7 mol%, 5.6 mg) or commercial available homogeneous and heterogeneous catalysts (0.7 mol%), a benzyl alcohol **2a** (1.0 mmol), and acetic acid (1.2 mol equiv). The mixture was shaken by a shaker (16 Hz, Petisyzer) for 12 h at 50°C. During the reaction, the reaction mixture was monitored with GC with decane as an internal standard.

Table 1:

To a 4 mL vial was added a *p*-phenol sulfonic acid-formaldehyde catalyst **1a** (0.7 mol%, 5.6 mg),

an alcohol **2** (1.0 mmol), and acetic acid (1.2 mol equiv). The mixture was shaken by a shaker (16 Hz, Petisyzer) for 12 h at 50-80 °C. After the reaction, the catalyst **1a** was filtered by filtration and washed with acetone. The filtrate was evaporated to give the corresponding acetate **3**.

Table 2:

To a 4 mL vial was added *p*-phenol sulfonic acid-formaldehyde **1a** (0.7 mol%, 5.6 mg), a carboxylic acid **4** (1.0 mmol), and methanol (5 mol equiv). The mixture was shaken by a shaker (16 Hz, Petisyzer) for 12 h at 50-60 °C. After the reaction, the catalyst **1a** was filtered by filtration and washed with acetone. The filtrate was evaporated to give the corresponding methyl esters **5**.

General procedure for the esterification in water

Table 3:

To a 4 mL vial was added a *p*-phenol sulfonic acid-formaldehyde catalyst **1a** (3 mol%, 24 mg), an alcohol **2** (1.0 mmol), and acetic acid (5 mol equiv) in water (3.0 M). The mixture was shaken by a shaker (16 Hz, Petisyzer) for 48 h at 80 °C. After the reaction, the catalyst **1a** was filtered by filtration and washed with acetone. The filtrate was evaporated to give the corresponding acetate **3**.

Table 4:

To a 4 mL vial was added *p*-phenol sulfonic acid-formaldehyde **1a** (3 mol%, 24 mg), a carboxylic acid **4** (1.0 mmol), and an alcohol (10 mol equiv) in water (3.0 M). The mixture was shaken by a shaker (16 Hz, Petisyzer) for 48 h at 80 °C. After the reaction, the catalyst **1a** was filtered by filtration and washed with acetone. The filtrate was evaporated to give the corresponding methyl esters **5**.

Figure 4:

Esterification:

To a 4 mL vial was added *p*-phenol sulfonic acid-formaldehyde **1a** (0.7 mol%, 5.6 mg), a decanoic acid **4b** (1.0 mmol), and an alcohol (10 mol equiv). The mixture was shaken by a shaker (16 Hz, Petisyzer) for 24 h at 50 °C. During the reaction, the reaction mixture was monitored by GC with decane as a internal standard.

Hydrolysis:

To a 4 mL vial was added *p*-phenol sulfonic acid-formaldehyde **1a** (0.7 mol%, 5.6 mg), a methyl decanoate **5b** (1.0 mmol), and an H_2O (10 mol equiv). The mixture was shaken by a shaker (16 Hz, Petisyzer) for 24 h at 50 °C. During the reaction, the reaction mixture was monitored by GC with decane as a internal standard.

General procedure for the transesterification

Figure 5:

To a 4 mL vial was added *p*-phenol sulfonic acid-formaldehyde **1a**, **1b** (0.7 mol%, 5.6 mg) or commercial available heterogeneous catalysts (0.7 mol%), **2d** (1.0 mmol), and ethyl acetate (10 mol equiv). The mixture was shaken by a shaker (16 Hz, Petisyzer) for 24 h at 80 °C without removal of ethanol. During the reaction, the reaction mixture was monitored by GC with decane as an internal standard.

Table 5:

To a 4 mL vial was added *p*-phenol sulfonic acid-formaldehyde **1a** (0.7 mol%, 5.6 mg), **2** (1.0

mmol), and ethyl acetate (10 mol equiv). The mixture was shaken by a shaker (16 Hz, Petisyzer) for 24 h at 80 °C without removal of ethanol. After the reaction, the catalyst **1a** was filtered by filtration and washed with acetone. The filtrate was evaporated to give the corresponding methyl esters **3**.

Table 6:

To a 4 mL vial was added *p*-phenol sulfonic acid-formaldehyde **1a** (0.7 mol%, 5.6 mg), **6** (1.0 mmol), and methanol (10 mol equiv) without removal of ethanol. The mixture was shaken by a shaker (16 Hz, Petisyzer) for 24 h at 80 °C. After the reaction, the catalyst **1a** was filtered by filtration and washed with acetone. The filtrate was evaporated to give the corresponding methyl esters **5**.

Synthesis of biodiesel fuel (FAME):

To a 4 mL vial was added *p*-phenol sulfonic acid-formaldehyde **1a** (0.7 mol%, 5.6 mg) or commercially available homogeneous and heterogeneous acid catalyst (0.7 mol%), oleic acid (1.0 mmol), and methanol (5 mol equiv). The mixture was shaken by a shaker (16 Hz, Petisyzer) for 12 h at 60 °C. After the reaction, the catalyst **1a** was filtered by filtration and washed with acetone. The filtrate was evaporated to give the corresponding biodiesel fuel **5g**.

Continuous flow reaction:

The acid catalyst **1a** (900 mg, 1.1 mmol) was packed into a glass column (15 cm× 6.6 mm bed reactor) that was attached to a heat block. A solution of the mixture of oleic acid and methanol was installed with a flow pump through capillary tubing. The flow reaction of oleic acid and

methanol (5 mol equiv) was carried at a flow rate of 10 μL/min through the **1a**-packed column at 80 °C (residence time: 18 min). The reaction mixture solution was collected from the outlet of column, and evaporated to give the corresponding biodiesel fuel **5g** in 92-96% conversion.

^{1}H and ^{13}C NMR data of products

benzyl acetate (3a)

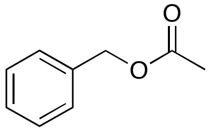

^{1}H NMR (500 MHz, $CDCl_3$): δ 2.10 (s, 3H), 5.11 (s, 2H), 7.31-7.39 (m, 5H). ^{13}C NMR (125 MHz, $CDCl_3$): δ 21.2, 66.5, 128.4, 128.7, 136.1, 171.0. GC-TOF HRMS: calcd for $C_9H_{10}O_2$ $[M]^+$ 150.0681, found 150.0672.

2-phenyl ethyl acetate (3b)

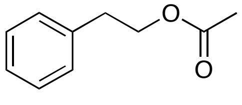

^{1}H NMR (500 MHz, $CDCl_3$): δ 2.04 (s, 3H), 2.94 (t, J=6.9 Hz, 2H), 4.28 (t, J=7.2 Hz, 2H), 7.21-7.32 (m, 5H). ^{13}C NMR (125 MHz, $CDCl_3$): δ 21.1, 35.2, 65.1, 126.7, 128.6, 129.0, 138.0, 171.2. GC-TOF HRMS: calcd for $C_{10}H_{12}O_2$ $[M]^+$ 164.0837, found 164.0846.

3-phenyl propyl acetate (3c)

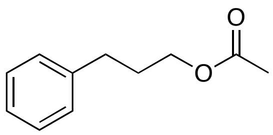

^{1}H NMR (500 MHz, $CDCl_3$): δ 1.93-1.99 (m, 2H), 2.05 (s, 3H), 2.69 (t, J=7.7 Hz, 2H), 4.09 (t,

J=6.6 Hz, 2H), 7.18-7.21 (m, 3H), 7.26-7.30 (m, 2H). ^{13}C NMR (125 MHz, $CDCl_3$): δ 21.1, 30.3, 32.3, 64.0, 126.2, 128.5, 128.6, 141.4, 171.3. GC-TOF HRMS: calcd for $C_{11}H_{14}O_2$ $[M]^+$ 178.0994, found 178.0989.

octyl acetate (3d)

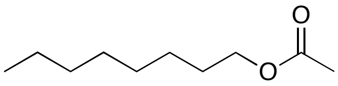

^{1}H NMR (500 MHz, $CDCl_3$): δ 0.88 (t, J=6.9 Hz, 3H), 1.22-1.38 (m, 10H), 1.59-1.65 (m, 2H), 2.05 (s, 3H), 4.05 (t, J = 6.9 Hz, 2H). ^{13}C NMR (125 MHz, $CDCl_3$): δ 14.2, 21.2, 22.8, 26.1, 28.8, 29.3, 29.4, 31.9, 64.8, 171.4. GC-TOF HRMS: calcd for $C_{10}H_{20}O_2$ $[M]^+$ 172.1463, found 172.1491.

decyl acetate (3e)

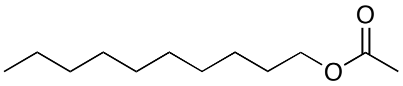

^{1}H NMR (500 MHz, $CDCl_3$): δ 0.88 (t, J=6.9 Hz, 3H), 1.22-1.38 (m, 14H), 1.59-1.65 (m, 2H), 2.05 (s, 3H), 4.05 (t, J = 6.9 Hz, 2H). ^{13}C NMR (125 MHz, $CDCl_3$): δ 14.3, 21.2, 22.8, 26.1, 28.8, 29.4, 29.4, 29.7, 29.7, 32.0, 64.8, 171.4. GC-TOF HRMS: calcd for $C_{12}H_{24}O_2$ $[M]^+$ 200.1776, found 200.1815.

dodecyl acetate (3f)

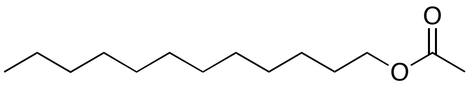

^{1}H NMR (500 MHz, $CDCl_3$): δ 0.88 (t, J=7.2 Hz, 3H), 1.22-1.37 (m, 16H), 1.59-1.65 (m, 2H), 2.05 (s, 3H), 4.05 (t, J = 6.9 Hz, 2H). ^{13}C NMR (125 MHz, $CDCl_3$): δ 14.3, 21.2, 22.8, 26.1, 28.8, 29.4,

29.5, 29.7, 29.7, 29.8, 29.8, 32.1, 64.8, 171.4. GC-TOF HRMS: calcd for $C_{14}H_{28}O_2$ $[M]^+$ 228.2089, found 228.2105.

3,7-dimethyloctyl acetate (3g)

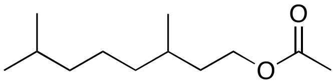

^{1}H NMR (500 MHz, CDCl$_3$): δ 0.87 (d, *J*=6.9 Hz, 6H), 0.90 (d, *J*=6.9 Hz, 3H), 1.10-1.16 (m, 3H), 1.21-1.34 (m, 3H), 1.39-1.46 (s, 1H), 1.48-1.56 (m, 2H), 1.62-1.69 (m, 1H), 2.04 (s, 3 H), 4.05-4.15 (m, 2H). ^{13}C NMR (125 MHz, CDCl$_3$): δ 19.7, 21.2, 22.7, 22.8, 24.7, 28.1, 30.0, 35.6, 37.3, 39.4, 63.3, 171.4. GC-TOF HRMS: calcd for $C_{12}H_{24}O_2$ $[M]^+$ 200.1773, found 200.1768.

cyclohexyl acetate (3h)

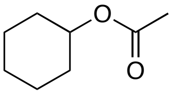

^{1}H NMR (500 MHz, CDCl$_3$): δ 1.21–1.28 (m, 1H), 1.32-1.44 (m, 3H), 1.52-1.57 (s, 2H), 1.70-1.75 (m, 2H), 1.84-1.87 (m, 2H), 2.03 (s, 3H) 4.71-4.76 (m, 1H). ^{13}C NMR (125 MHz, CDCl$_3$): δ 21.6, 24.0, 25.5, 31.8, 72.8, 170.8. GC-TOF HRMS: calcd for $C_8H_{14}O_2$ $[M]^+$ 142.0994, found 142.0976.

cyclooctyl acetate (3i)

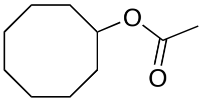

^{1}H NMR (500 MHz, CDCl$_3$): δ 1.45-41.84 (m, 14H), 2.02 (s, 3H), 4.93 (sept, J = 4.2 Hz, 1H). ^{13}C NMR (125 MHz, CDCl$_3$): δ 21.7, 23.1, 25.5, 27.2, 31.6, 75.6, 170.7. GC-TOF HRMS: calcd for $C_{10}H_{18}O_2$ $[M]^+$ 170.1307, found 170.1323.

octan-2-yl acetate (3j)

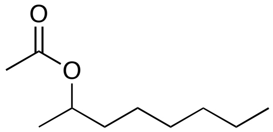

^{1}H NMR (500 MHz, $CDCl_3$): δ 0.88 (t, *J*=6.9 Hz, 3H), 1.20 (d, *J*=6.3 Hz, 3H), 1.22-1.35 (m, 8H), 1.41-1.50 (m 1H), 1.54-1.62 (m, 1H), 2.03 (s, 3 H), 4.89 (sext, *J*=6.3 Hz, 1H). ^{13}C NMR (125 MHz, $CDCl_3$): δ 14.2, 20.1, 21.4, 22.7, 25.5, 29.2, 31.9, 36.1, 71.6, 171.0. GC-TOF HRMS: calcd for $C_{10}H_{18}O_2$ $[M]^+$ 172.1463, found 172.1447.

hexadecyl acetate (3k)

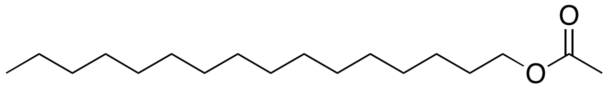

^{1}H NMR (400 MHz, $CDCl_3$): δ 0.88 (t, *J*=6.7 Hz, 3H), 1.25-1.38 (m, 26H), 1.58-1.65 (m, 2H), 2.04 (s, 3H), 4.03 (t, *J* = 6.7 Hz, 2H). ^{13}C NMR (100 MHz, $CDCl_3$): δ 14.1, 20.9, 22.7, 25.9, 28.6, 29.2, 29.3, 29.5, 29.5, 29.6, 29.6, 29.7, 31.9, 64.6, 171.1. GC-TOF HRMS: calcd for $C_{18}H_{36}O_2$ $[M]^+$ 284.2715, found 284.2710.

4-methylbenzyl acetate (3l)

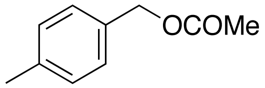

^{1}H NMR (400 MHz, $CDCl_3$): δ 2.08 (s, 2H), 2.35 (s, 3H), 5.06 (s, 2H), 7.16-7.25 (m, 4H). ^{13}C NMR (100 MHz, $CDCl_3$): δ 21.0, 21.2, 66.2, 128.4, 129.2, 132.9, 138.1, 170.9. GC-TOF HRMS: calcd for $C_{10}H_{12}O_2$ $[M]^+$ 164.0837, found 164.0839.

2-methylbenzyl acetate (3m)

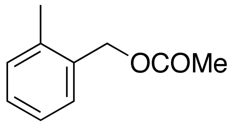

^{1}H NMR (400 MHz, $CDCl_3$): δ 2.10 (s, 3H), 2.35 (s, 3H), 5.12 (s, 2H), 7.20-7.33 (m, 4H). ^{13}C NMR (100 MHz, $CDCl_3$): δ 18.8, 20.9, 64.7, 126.0, 128.5, 129.2, 130.3, 133.8, 136.9, 172.7. GC-TOF HRMS: calcd for $C_{10}H_{12}O_2$ $[M]^+$ 164.0837, found 164.0840.

2-methoxybenzyl acetate (3n)

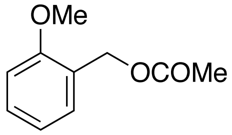

^{1}H NMR (400 MHz, $CDCl_3$): δ 2.10 (s, 3H), 3.84 (s, 3H), 5.17 (s, 2H), 6.85-7.33 (m, 4H). ^{13}C NMR (100 MHz, $CDCl_3$): δ 21.0, 55.3, 61.7, 110.4, 120.3, 124.2, 129.5, 129.7, 157.4, 170.9. GC-TOF HRMS: calcd for $C_{10}H_{12}O_3$ $[M]^+$ 180.0786, found 180.0759.

3-methoxybenzyl acetate (3o)

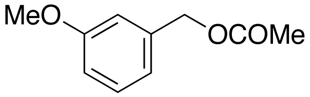

^{1}H NMR (400 MHz, $CDCl_3$): δ 2.10 (s, 3H), 3.81 (s, 3H), 5.08 (s, 2H), 6.85-7.30 (m, 4H). ^{13}C NMR (100 MHz, $CDCl_3$): δ 20.9, 55.2, 66.1, 113.6, 113.7, 120.4, 129.6, 137.4, 159.7, 170.8. GC-TOF HRMS: calcd for $C_{10}H_{12}O_3$ $[M]^+$ 180.0786, found 180.0799.

4-chlorobenzyl acetate (3p)

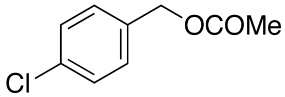

^{1}H NMR (400 MHz, $CDCl_3$): δ 2.10 (s, 3H), 5.06 (s, 2H), 7.31-7.39 (m, 4H). ^{13}C NMR (100 MHz, $CDCl_3$): δ 20.9, 65.5, 128.8, 129.6, 134.2, 134.4, 170.8. GC-TOF HRMS: calcd for $C_9H_9ClO_2$ $[M]^+$ 184.0291, found 184.0293.

4-nitrobenzyl acetate (3q)

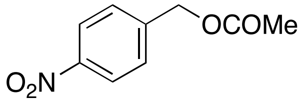

^{1}H NMR (400 MHz, $CDCl_3$): δ 2.15 (s, 3H), 5.20 (s, 2H), 7.53 (d, J = 8.7 Hz, 2H), 8.24 (d, J = 8.7 Hz, 2H). ^{13}C NMR (100 MHz, $CDCl_3$): δ 20.9, 29.7, 64.8, 123.8, 128.4, 143.2. GC-TOF HRMS: calcd for $C_9H_9O_4N$ $[M]^+$ 195.0531, found 195.0578.

methyl octanoate (5a)

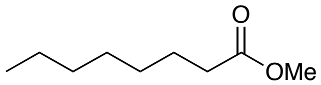

^{1}H NMR (500 MHz, $CDCl_3$): δ 0.88 (t, J = 6.9 Hz, 3H), 1.22-1.34 (m, 8H), 1.59-1.65 (m, 2H), 2.30 (t, J = 7.5 Hz, 2H), 3.67 (s, 3H). ^{13}C NMR (125 MHz, $CDCl_3$): δ 14.2, 22.7, 25.1, 29.1, 29.3, 31.8, 34.3, 51.6, 174.5. GC-TOF HRMS: calcd for $C_9H_{18}O_2$ $[M]^+$ 158.1307, found 158.1340.

methyl decanoate (5b)

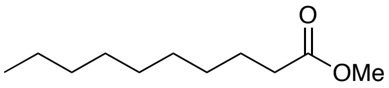

^{1}H NMR (500 MHz, $CDCl_3$): δ 0.88 (t, J = 6.9 Hz, 3H), 1.22-1.35 (m, 10H), 1.59-1.65 (m, 2H), 2.30 (t, J = 7.5 Hz, 2H), 3.67 (s, 3H). ^{13}C NMR (125 MHz, $CDCl_3$): δ 14.2, 22.8, 25.1, 29.3, 29.4, 29.4, 29.6, 32.0, 34.3, 51.6, 174.5. GC-TOF HRMS: calcd for $C_{11}H_{22}O_2$ $[M]^+$ 186.1620, found 186.1639.

methyl dodecanoate (5c)

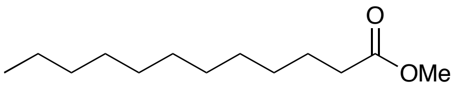

^{1}H NMR (500 MHz, $CDCl_3$): δ 0.88 (t, J = 6.9 Hz, 3H), 1.22-1.35 (m, 16H), 1.58-1.65 (m, 2H), 2.30 (t, J = 7.5 Hz, 2H), 3.67 (s, 3H). ^{13}C NMR (125 MHz, $CDCl_3$): δ 14.3, 22.8, 25.1, 29.3, 29.4, 29.5, 29.6, 29.7, 29.7, 32.1, 34.3, 51.6, 174.5. GC-TOF HRMS: calcd for $C_{13}H_{26}O_2$ $[M]^+$ 214.1933, found 214.1935.

methyl hexadecanoate (5d)

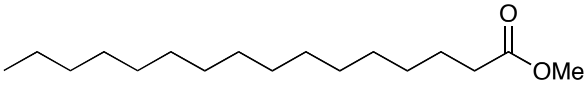

^{1}H NMR (500 MHz, $CDCl_3$): δ 0.88 (t, J = 6.9 Hz, 3H), 1.22-1.35 (m, 24H), 1.59-1.65 (m, 2H), 2.30 (t, J = 7.7 Hz, 2H), 3.67 (s, 3H). ^{13}C NMR (125 MHz, $CDCl_3$): δ 14.3, 22.8, 25.1, 29.3, 29.4, 29.5, 29.6, 29.7, 29.8, 29.8, 29.8, 29.8. 29.8, 32.0, 34.3, 51.6, 174.5. GC-TOF HRMS: calcd for $C_{17}H_{34}O_2$ $[M]^+$ 270.2559, found 270.2559.

methyl octadecanoate (5e)

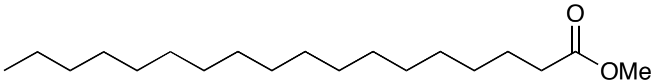

^1H NMR (500 MHz, $CDCl_3$): δ 0.88 (t, J = 7.2 Hz, 3H), 1.22-1.35 (m, 28H), 1.59-1.65 (m, 2H), 2.30 (t, J = 7.5 Hz, 2H), 3.67 (s, 3H). ^{13}C NMR (125 MHz, $CDCl_3$): δ 14.3, 22.8, 25.1, 29.3, 29.4, 29.5, 29.6, 29.7, 29.8, 29.8, 29.8, 29.8. 29.8, 29.8, 29.8, 32.1, 34.3, 51.6, 174.5. GC-TOF HRMS: calcd for $C_{19}H_{38}O_2$ $[M]^+$ 298.2872, found 298.2861.

methyl heptanoate (5f)

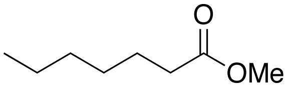

^1H NMR (400 MHz, $CDCl_3$): δ 0.88 (t, J = 7.1 Hz, 3H), 1.22-1.35 (m, 6H), 1.60-1.64 (m, 2H), 2.30 (t, J = 7.9 Hz, 2H), 3.67 (s, 3H). ^{13}C NMR (100 MHz, $CDCl_3$): δ 14.0, 22.4, 24.8, 28.8, 31.4, 34.1, 51.4, 174.3. GC-TOF HRMS: calcd for $C_8H_{16}O_2$ $[M]^+$ 144.1150, found 144.1144.

methyl oleate (5g)

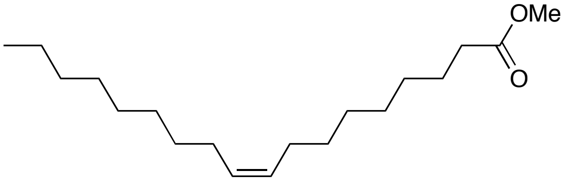

^1H NMR (500 MHz, $CDCl_3$): δ 0.88 (t, J = 6.9 Hz, 3H), 1.22-1.35 (m, 20H), 1.59-1.65 (m, 2H), 1.99-2.02 (m, 4H), 2.30 (t, J = 7.7 Hz, 2H), 3.67 (s, 3H), 5.31-5.38 (m, 2H). ^{13}C NMR (125 MHz, $CDCl_3$): δ 14.3, 22.8, 25.1, 27.3, 27.4, 29.2, 29.3, 29.3, 29.5, 29.5, 29.7, 29.8, 29.9, 32.1, 34.3, 51.6, 129.9, 130.2, 174.5. GC-TOF HRMS: calcd for $C_{19}H_{36}O_2$ $[M]^+$ 296.2715, found 296.2732.

octyl octanoate (6a)

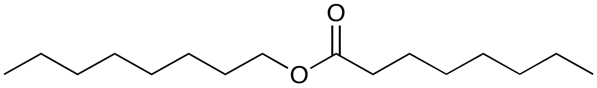

^1H NMR (500 MHz, $CDCl_3$): δ 0.88 (m, 6H), 1.23-1.35 (m, 18H), 1.59-1.65 (m, 4H), 2.29 (t, J = 7.5 Hz, 2H), 4.06 (t, J = 6.9 Hz, 2H). ^{13}C NMR (125 MHz, $CDCl_3$): δ 14.2, 14.2, 22.8, 22.8, 25.2, 26.1, 28.8, 29.1, 29.3, 29.3, 29.4, 31.8, 31.9, 34.6, 64.6, 174.2. GC-TOF HRMS: calcd for $C_{16}H_{32}O_2$ $[M]^+$ 256.2402, found 256.2389.

butyl decanoate (6b)

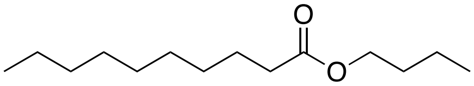

^1H NMR (400 MHz, $CDCl_3$): δ 0.88 (m, 6H), 1.19-1.36 (m, 14H), 1.50-1.57 (m, 4H), 2.22 (t, J = 7.1 Hz, 2H), 4.00 (t, J = 6.3 Hz, 2H). ^{13}C NMR (100 MHz, $CDCl_3$): δ 13.7, 14.1, 19.1, 22.6, 25.0, 20.1, 29.2, 29.3, 29.4, 30.7, 31.8, 34.4, 64.1, 174.0. GC-TOF HRMS: calcd for $C_{14}H_{28}O_2$ $[M]^+$ 228.2089, found 228.2143.

methyl 4-phenylbutanoate (6c)

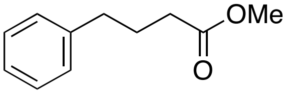

^1H NMR (400 MHz, $CDCl_3$): δ 1.98-2.05 (m, 2H), 2.39 (t, J = 7.6 Hz, 2H), 2.71 (t, J = 7.6 Hz, 2H), 3.72 (s, 3H), 7.22-7.25 (m, 3H), 7.31-7.35 (m, 2H). ^{13}C NMR (100 MHz, $CDCl_3$): δ 26.5, 33.4, 35.1, 51.5, 126.0, 128.4, 141.4, 174.0. GC-TOF HRMS: calcd for $C_{11}H_{14}O_2$ $[M]^+$ 178.0994, found 178.1005.

octyl 4-phenylbutanoate (6d)

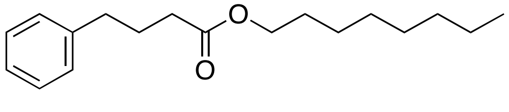

^{1}H NMR (300 MHz, $CDCl_3$): δ 0.93 (t, *J* = 6.9 Hz, 3H), 1.27-1.30 (m, 10H), 1.60-1.61 (m, 2H), 1.90-2.00 (m, 2H), 2.32 (t, *J* = 7.8 Hz, 2H), 2.65 (t, *J* = 7.8 Hz, 2H), 4.06 (t, *J* = 6.6 Hz, 2H), 7.17-7.21 (m, 3H), 7.26-7.31 (m, 2H). ^{13}C NMR (75 MHz, $CDCl_3$): δ 14.1, 22.6, 25.9, 26.5, 28.6, 29.1, 31.7, 33.6, 35.1, 64.5, 125.9, 128.3, 128.4, 141.4, 173.5. GC-TOF HRMS: calcd for $C_{18}H_{28}O_2$ $[M]^+$ 276.2089, found 276.2141.

butyl 4-phenylbutanoate (6e)

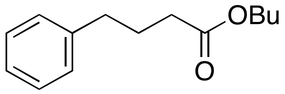

^{1}H NMR (400 MHz, $CDCl_3$): δ 0.93 (t, *J* = 7.6 Hz, 3H), 1.32-1.43 (m, 2H), 1.57-1.64 (m, 2H), 1.92-2.00 (m, 2H), 2.32 (t, *J* = 7.2 Hz, 2H), 2.65 (t, *J* = 7.6 Hz, 2H), 4.07 (t, *J* = 6.8 Hz, 2H), 7.16-7.19 (m, 3H), 7.28-7.30 (m, 2H). ^{13}C NMR (100 MHz, $CDCl_3$): δ 14.0, 19.2, 26.6, 30.7, 33.7, 35.2, 64.2, 125.9, 128.4, 141.5, 173.6. GC-TOF HRMS: calcd for $C_{14}H_{20}O_2$ $[M]^+$ 220.1463, found 220.1448.

SUPPORTING INFOMATION II

In-Water and Neat Batch and Continuous-Flow Direct Esterification and Transesterification by a Porous Polymeric Acid Catalyst

Heeyoel Baek[1,3], Maki Minakawa[1], Yoichi M. A. Yamada[1,*], Jin Wook Han[3], and Yasuhiro Uozumi[1,2,*]

[1]RIKEN Center for Sustainable Resource Science, Wako, Saitama 351-0198, Japan
[2]Institute for Molecular Science (IMS), Myodaiji, Okazaki, Aichi 444-8787, Japan
[3]Department of Chemistry, Hanyang University, Seoul 04763, Korea
[*]ymayamada@riken.jp, uo@ims.ac.jp

^{1}H and ^{13}C NMR spectra

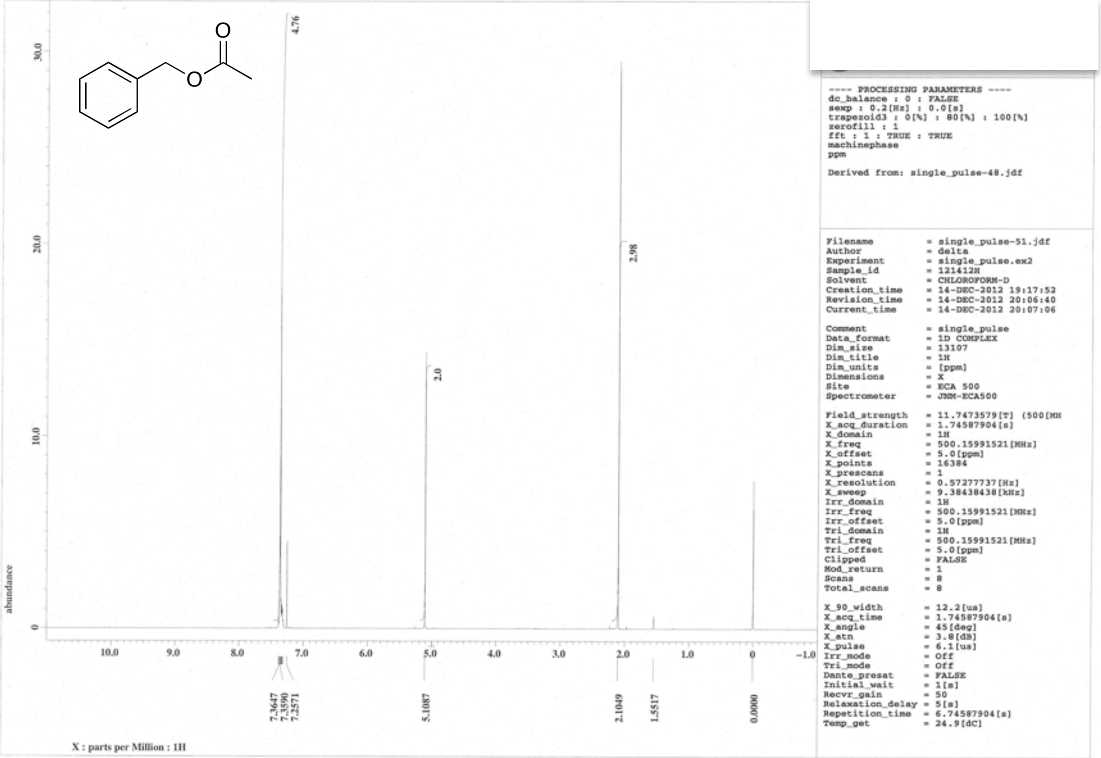
---- PROCESSING PARAMETERS ----
dc_balance : 0 : FALSE
sexp : 0.2[Hz] : 0.0[s]
trapezoid3 : 0[%] : 80[%] : 100[%]
zerofill : 1
fft : 1 : TRUE : TRUE
machinephase
ppm
Derived from: single_pulse-48.jdf
Filename = single_pulse-51.jdf
Author = delta
Experiment = single_pulse.ex2
Sample_id = 121412H
Solvent = CHLOROFORM-D
Creation_time = 14-DEC-2012 19:17:52
Revision_time = 14-DEC-2012 20:06:40
Current_time = 14-DEC-2012 20:07:06
Comment = single_pulse
Data_format = 1D COMPLEX
Dim_size = 13107
Dim_title = 1H
Dim_units = [ppm]
Dimensions = X
Site = ECA 500
Spectrometer = JNM-ECA500
Field_strength = 11.7473579[T] (500[MH
X_acq_duration = 1.74587904[s]
X_domain = 1H
X_freq = 500.15991521[MHz]
X_offset = 5.0[ppm]
X_points = 16384
X_prescans = 1
X_resolution = 0.57277737[Hz]
X_sweep = 9.38438438[kHz]
Irr_domain = 1H
Irr_freq = 500.15991521[MHz]
Irr_offset = 5.0[ppm]
Tri_domain = 1H
Tri_freq = 500.15991521[MHz]
Tri_offset = 5.0[ppm]
Clipped = FALSE
Mod_return = 1
Scans = 8
Total_scans = 8
X_90_width = 12.2[us]
X_acq_time = 1.74587904[s]
X_angle = 45[deg]
X_atn = 3.8[dB]
X_pulse = 6.1[us]
Irr_mode = Off
Tri_mode = Off
Dante_presat = FALSE
Initial_wait = 1[s]
Recvr_gain = 50
Relaxation_delay = 5[s]
Repetition_time = 6.74587904[s]
Temp_get = 24.9[dC]
4.76
2.98
2.0
abundance
7.3647
7.3590
7.2571
5.1087
2.1049
1.5517
0.0000
X : parts per Million : 1H

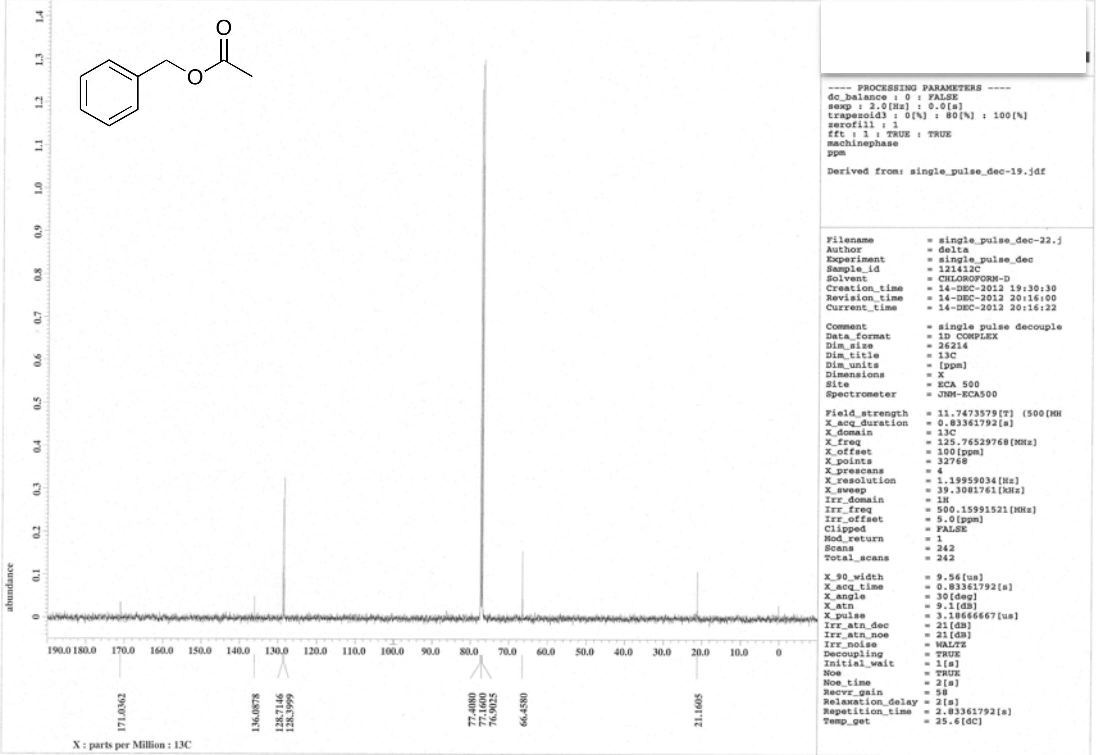
---- PROCESSING PARAMETERS ----
dc_balance : 0 : FALSE
sexp : 2.0[Hz] : 0.0[s]
trapezoid3 : 0[%] : 80[%] : 100[%]
zerofill : 1
fft : 1 : TRUE : TRUE
machinephase
ppm
Derived from: single_pulse_dec-19.jdf
Filename = single_pulse_dec-22.j
Author = delta
Experiment = single_pulse_dec
Sample_id = 121412C
Solvent = CHLOROFORM-D
Creation_time = 14-DEC-2012 19:30:30
Revision_time = 14-DEC-2012 20:16:00
Current_time = 14-DEC-2012 20:16:22
Comment = single pulse decouple
Data_format = 1D COMPLEX
Dim_size = 26214
Dim_title = 13C
Dim_units = [ppm]
Dimensions = X
Site = ECA 500
Spectrometer = JNM-ECA500
Field_strength = 11.7473579[T] (500[MH
X_acq_duration = 0.83361792[s]
X_domain = 13C
X_freq = 125.76529768[MHz]
X_offset = 100[ppm]
X_points = 32768
X_prescans = 4
X_resolution = 1.19959034[Hz]
X_sweep = 39.3081761[kHz]
Irr_domain = 1H
Irr_freq = 500.15991521[MHz]
Irr_offset = 5.0[ppm]
Clipped = FALSE
Mod_return = 1
Scans = 242
Total_scans = 242
X_90_width = 9.56[us]
X_acq_time = 0.83361792[s]
X_angle = 30[deg]
X_atn = 9.1[dB]
X_pulse = 3.18666667[us]
Irr_atn_dec = 21[dB]
Irr_atn_noe = 21[dB]
Irr_noise = WALTZ
Decoupling = TRUE
Initial_wait = 1[s]
Noe = TRUE
Noe_time = 2[s]
Recvr_gain = 58
Relaxation_delay = 2[s]
Repetition_time = 2.83361792[s]
Temp_get = 25.6[dC]
abundance
171.0362
136.0878
128.7146
128.3999
77.4080
77.1600
76.9025
66.4580
21.1605
X : parts per Million : 13C

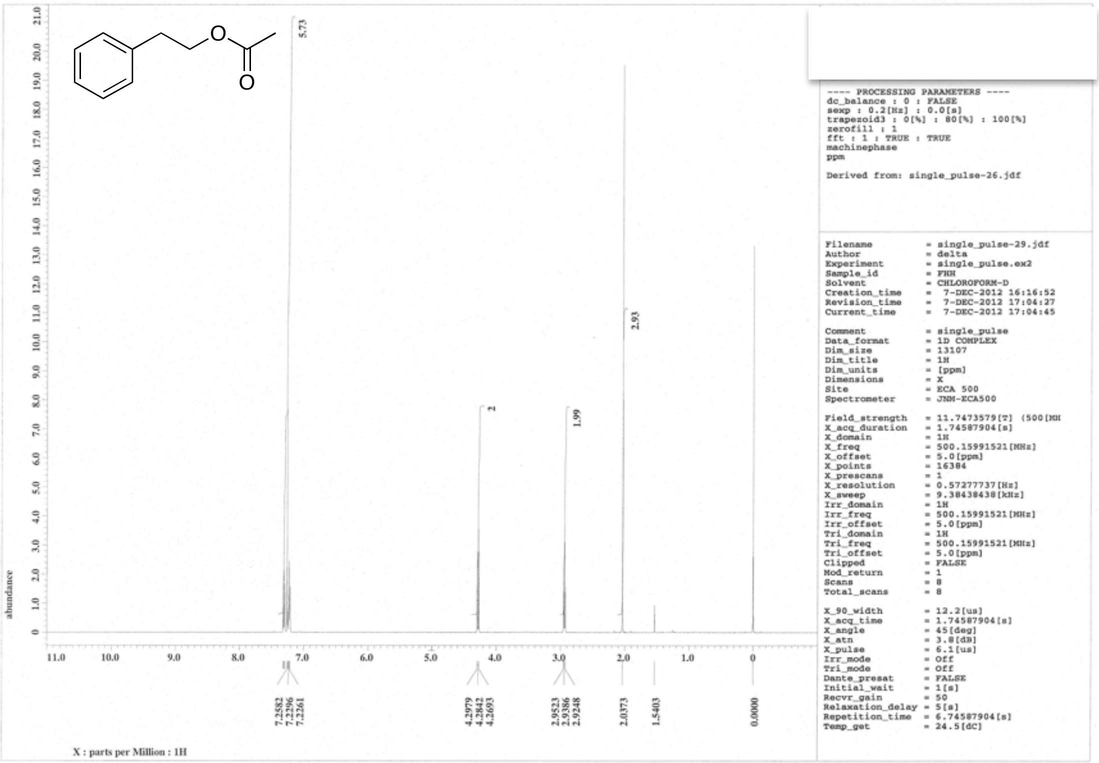
O
O
5.73
2
1.99
2.93
abundance
X : parts per Million : 1H
7.2582
7.2396
7.2261
4.2979
4.2842
4.2693
2.9523
2.9386
2.9248
2.0373
1.5403
0.0000
---- PROCESSING PARAMETERS ----
dc_balance : 0 : FALSE
sexp : 0.2[Hz] : 0.0[s]
trapezoid3 : 0[%] : 80[%] : 100[%]
zerofill : 1
fft : 1 : TRUE : TRUE
machinephase
ppm
Derived from: single_pulse-26.jdf
Filename = single_pulse-29.jdf
Author = delta
Experiment = single_pulse.ex2
Sample_id = FHH
Solvent = CHLOROFORM-D
Creation_time = 7-DEC-2012 16:16:52
Revision_time = 7-DEC-2012 17:04:27
Current_time = 7-DEC-2012 17:04:45
Comment = single_pulse
Data_format = 1D COMPLEX
Dim_size = 13107
Dim_title = 1H
Dim_units = [ppm]
Dimensions = X
Site = ECA 500
Spectrometer = JNM-ECA500
Field_strength = 11.7473579[T] (500[MH
X_acq_duration = 1.74587904[s]
X_domain = 1H
X_freq = 500.15991521[MHz]
X_offset = 5.0[ppm]
X_points = 16384
X_prescans = 1
X_resolution = 0.57277737[Hz]
X_sweep = 9.38438438[kHz]
Irr_domain = 1H
Irr_freq = 500.15991521[MHz]
Irr_offset = 5.0[ppm]
Tri_domain = 1H
Tri_freq = 500.15991521[MHz]
Tri_offset = 5.0[ppm]
Clipped = FALSE
Mod_return = 1
Scans = 8
Total_scans = 8
X_90_width = 12.2[us]
X_acq_time = 1.74587904[s]
X_angle = 45[deg]
X_atn = 3.8[dB]
X_pulse = 6.1[us]
Irr_mode = Off
Tri_mode = Off
Dante_presat = FALSE
Initial_wait = 1[s]
Recvr_gain = 50
Relaxation_delay = 5[s]
Repetition_time = 6.74587904[s]
Temp_get = 24.5[dC]

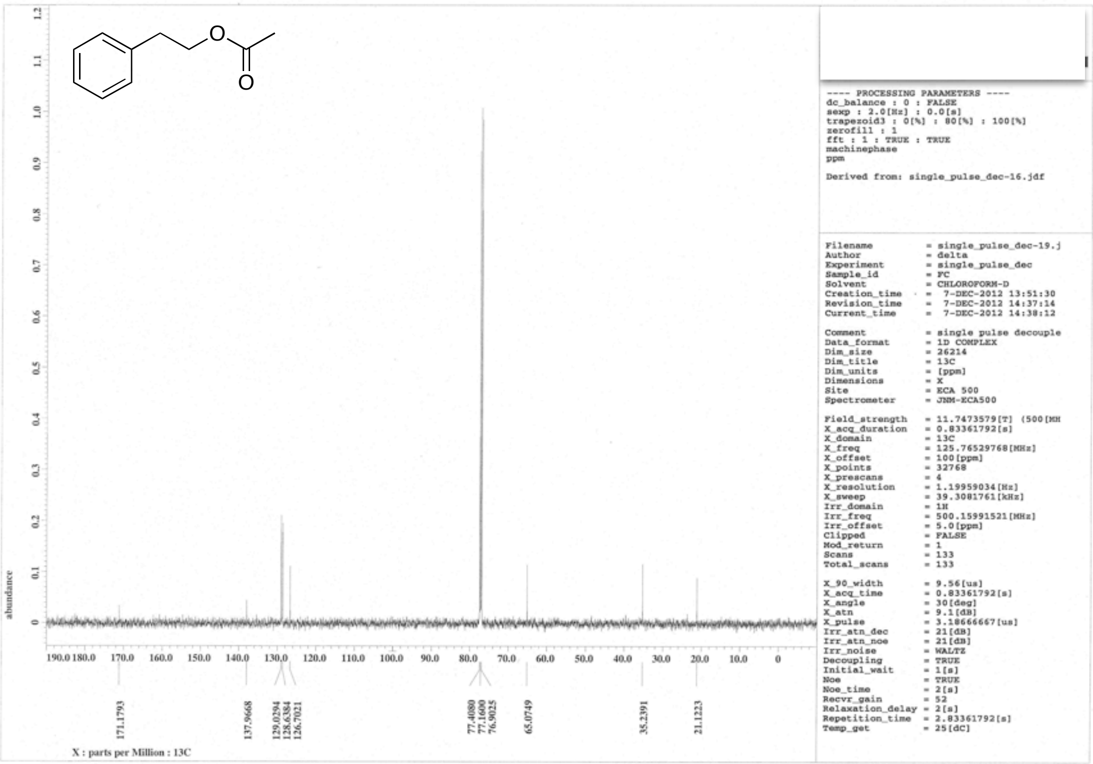
O
O
abundance
X : parts per Million : 13C
171.1793
137.9668
129.0294
128.6384
126.7021
77.4080
77.1600
76.9025
65.0749
35.2391
21.1223
---- PROCESSING PARAMETERS ----
dc_balance : 0 : FALSE
sexp : 2.0[Hz] : 0.0[s]
trapezoid3 : 0[%] : 80[%] : 100[%]
zerofill : 1
fft : 1 : TRUE : TRUE
machinephase
ppm
Derived from: single_pulse_dec-16.jdf
Filename = single_pulse_dec-19.j
Author = delta
Experiment = single_pulse_dec
Sample_id = FC
Solvent = CHLOROFORM-D
Creation_time = 7-DEC-2012 13:51:30
Revision_time = 7-DEC-2012 14:37:14
Current_time = 7-DEC-2012 14:38:12
Comment = single pulse decouple
Data_format = 1D COMPLEX
Dim_size = 26214
Dim_title = 13C
Dim_units = [ppm]
Dimensions = X
Site = ECA 500
Spectrometer = JNM-ECA500
Field_strength = 11.7473579[T] (500[MH
X_acq_duration = 0.83361792[s]
X_domain = 13C
X_freq = 125.76529768[MHz]
X_offset = 100[ppm]
X_points = 32768
X_prescans = 4
X_resolution = 1.19959034[Hz]
X_sweep = 39.3081761[kHz]
Irr_domain = 1H
Irr_freq = 500.15991521[MHz]
Irr_offset = 5.0[ppm]
Clipped = FALSE
Mod_return = 1
Scans = 133
Total_scans = 133
X_90_width = 9.56[us]
X_acq_time = 0.83361792[s]
X_angle = 30[deg]
X_atn = 9.1[dB]
X_pulse = 3.18666667[us]
Irr_atn_dec = 21[dB]
Irr_atn_noe = 21[dB]
Irr_noise = WALTZ
Decoupling = TRUE
Initial_wait = 1[s]
Noe = TRUE
Noe_time = 2[s]
Recvr_gain = 52
Relaxation_delay = 2[s]
Repetition_time = 2.83361792[s]
Temp_get = 25[dC]

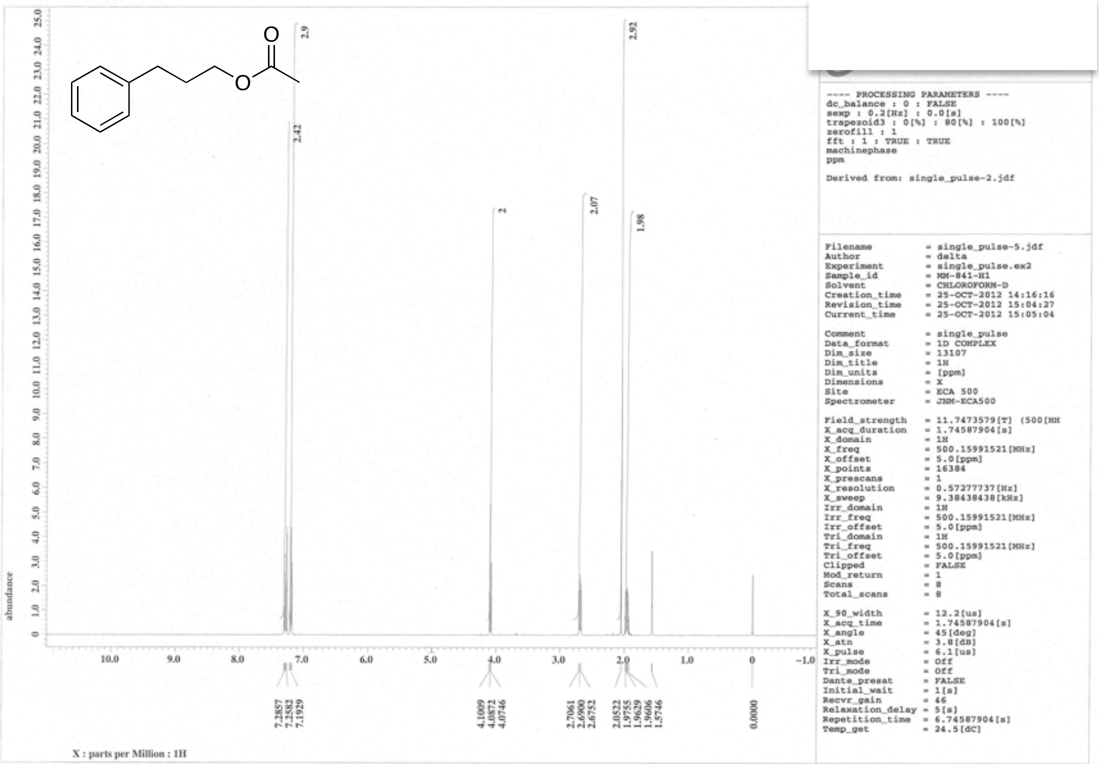
2.9
2.42
2
2.07
2.92
1.98
7.2857
7.2582
7.1929
4.1099
4.0872
4.0746
2.7061
2.6900
2.6752
2.0522
1.9755
1.9629
1.9606
1.5746
0.0000
abundance
X : parts per Million : 1H

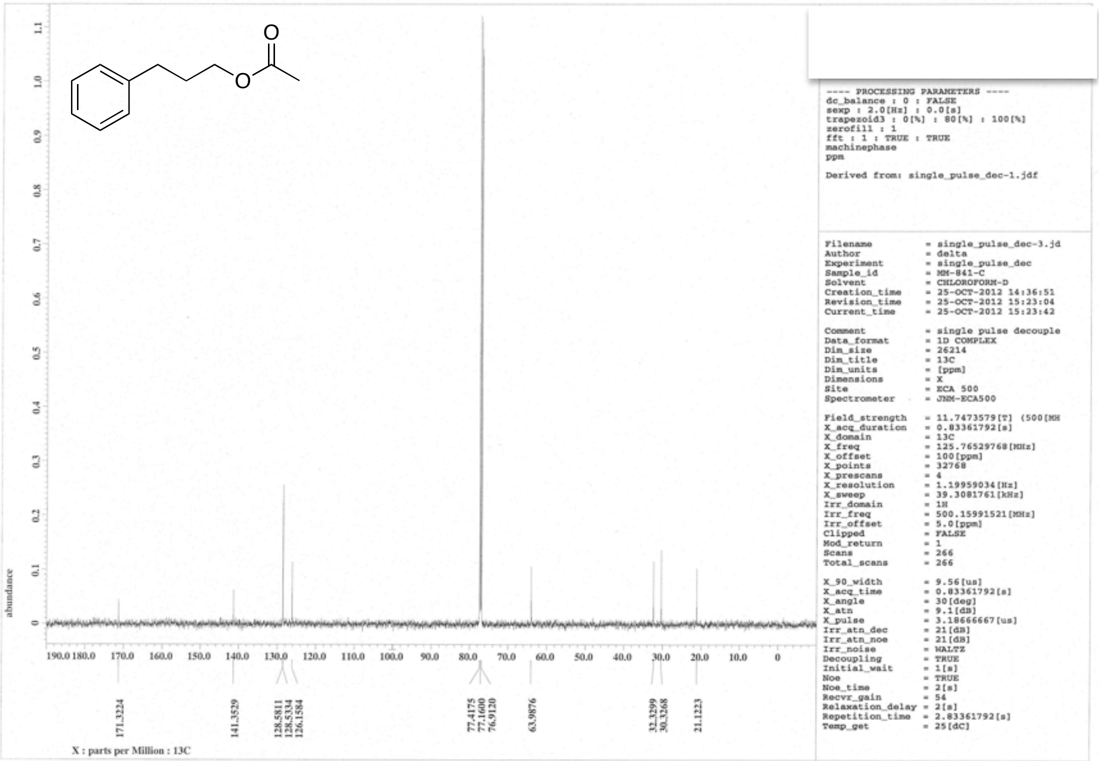
171.3224
141.3529
128.5811
128.5344
126.1584
77.4175
77.1600
76.9120
63.9876
32.3299
30.3268
21.1223
abundance
X : parts per Million : 13C

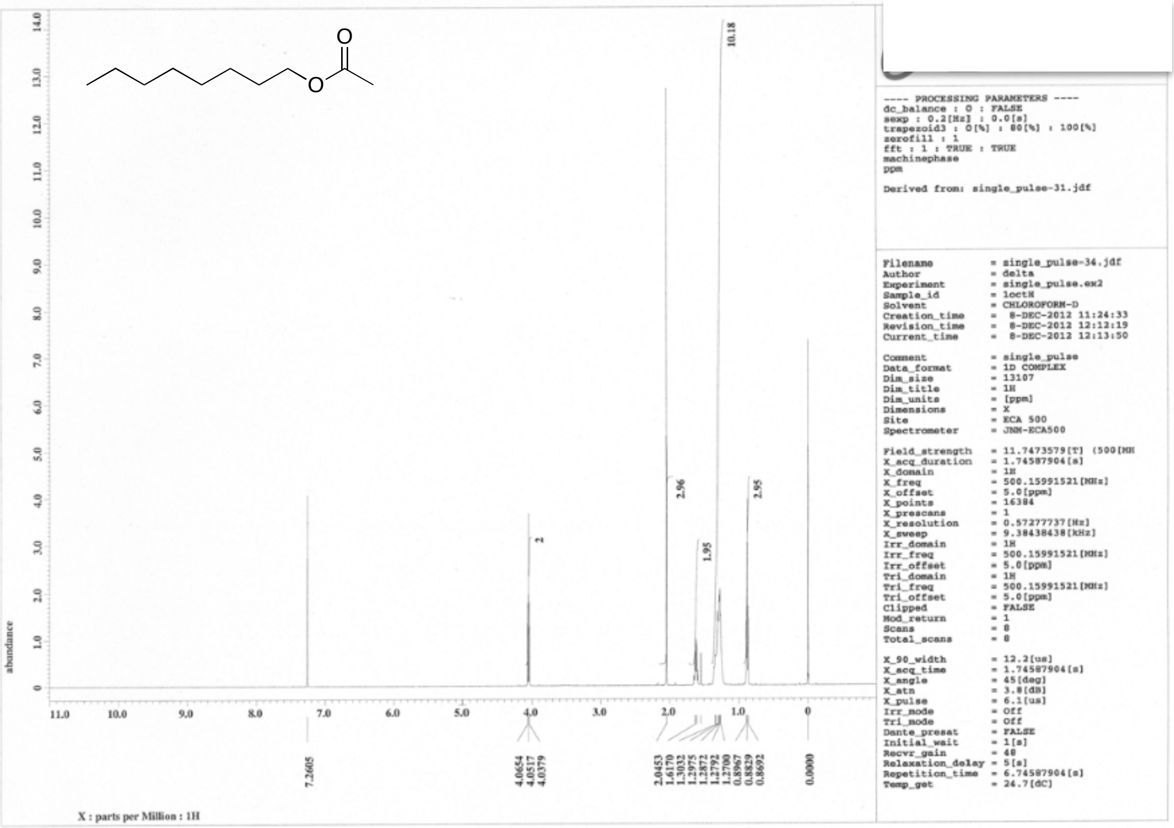
O
O
abundance
X : parts per Million : 1H
7.2605
4.0654
4.0517
4.0379
2.0453
1.6170
1.3032
1.2975
1.2872
1.2792
1.2700
0.8967
0.8829
0.8692
0.0000
2
2.96
1.95
10.18
2.95
---- PROCESSING PARAMETERS ----
dc_balance : 0 : FALSE
sexp : 0.2[Hz] : 0.0[s]
trapezoid3 : 0[%] : 80[%] : 100[%]
zerofill : 1
fft : 1 : TRUE : TRUE
machinephase
ppm
Derived from: single_pulse-31.jdf
Filename = single_pulse-34.jdf
Author = delta
Experiment = single_pulse.ex2
Sample_id = 1octH
Solvent = CHLOROFORM-D
Creation_time = 8-DEC-2012 11:24:33
Revision_time = 8-DEC-2012 12:12:19
Current_time = 8-DEC-2012 12:13:50
Comment = single_pulse
Data_format = 1D COMPLEX
Dim_size = 13107
Dim_title = 1H
Dim_units = [ppm]
Dimensions = X
Site = ECA 500
Spectrometer = JNM-ECA500
Field_strength = 11.7473579[T] (500[MH
X_acq_duration = 1.74587904[s]
X_domain = 1H
X_freq = 500.15991521[MHz]
X_offset = 5.0[ppm]
X_points = 16384
X_prescans = 1
X_resolution = 0.57277737[Hz]
X_sweep = 9.38438438[kHz]
Irr_domain = 1H
Irr_freq = 500.15991521[MHz]
Irr_offset = 5.0[ppm]
Tri_domain = 1H
Tri_freq = 500.15991521[MHz]
Tri_offset = 5.0[ppm]
Clipped = FALSE
Mod_return = 1
Scans = 8
Total_scans = 8
X_90_width = 12.2[us]
X_acq_time = 1.74587904[s]
X_angle = 45[deg]
X_atn = 3.8[dB]
X_pulse = 6.1[us]
Irr_mode = Off
Tri_mode = Off
Dante_presat = FALSE
Initial_wait = 1[s]
Recvr_gain = 48
Relaxation_delay = 5[s]
Repetition_time = 6.74587904[s]
Temp_get = 24.7[dC]

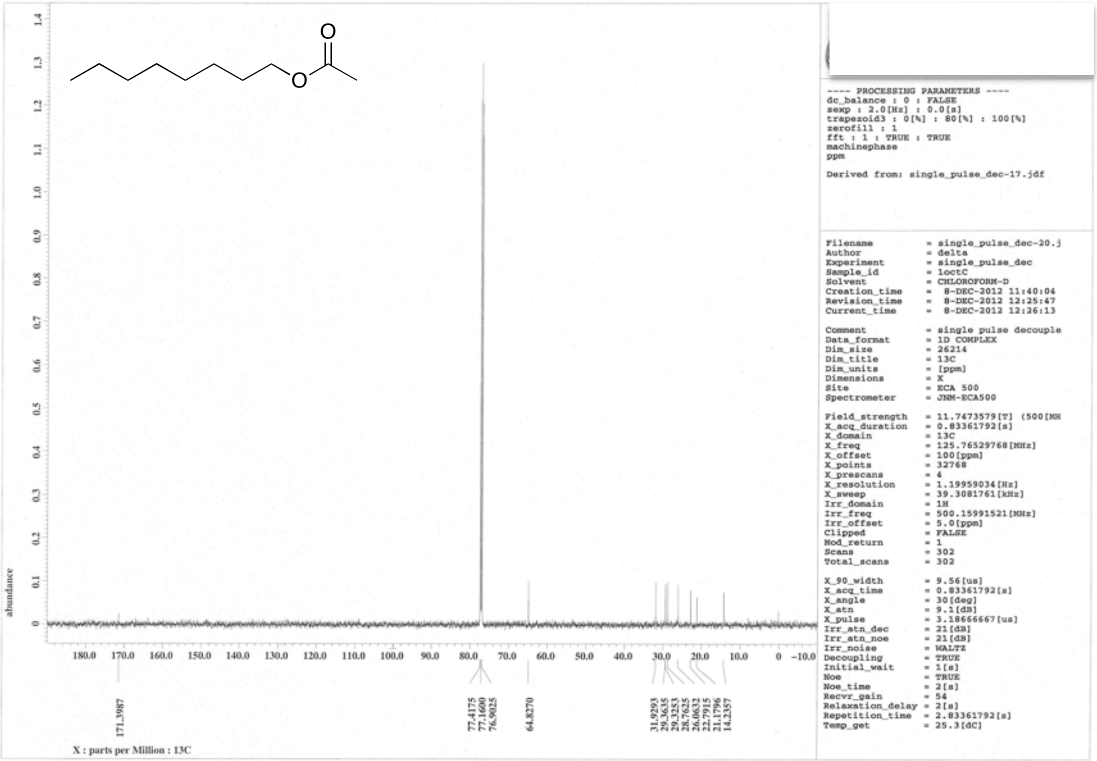
O
O
abundance
X : parts per Million : 13C
171.3987
77.4175
77.1600
76.9025
64.8270
31.9293
29.3635
29.3253
28.7625
26.0632
22.7915
21.1796
14.2357
---- PROCESSING PARAMETERS ----
dc_balance : 0 : FALSE
sexp : 2.0[Hz] : 0.0[s]
trapezoid3 : 0[%] : 80[%] : 100[%]
zerofill : 1
fft : 1 : TRUE : TRUE
machinephase
ppm
Derived from: single_pulse_dec-17.jdf
Filename = single_pulse_dec-20.j
Author = delta
Experiment = single_pulse_dec
Sample_id = 1octC
Solvent = CHLOROFORM-D
Creation_time = 8-DEC-2012 11:40:04
Revision_time = 8-DEC-2012 12:25:47
Current_time = 8-DEC-2012 12:26:13
Comment = single pulse decouple
Data_format = 1D COMPLEX
Dim_size = 26214
Dim_title = 13C
Dim_units = [ppm]
Dimensions = X
Site = ECA 500
Spectrometer = JNM-ECA500
Field_strength = 11.7473579[T] (500[MH
X_acq_duration = 0.83361792[s]
X_domain = 13C
X_freq = 125.76529768[MHz]
X_offset = 100[ppm]
X_points = 32768
X_prescans = 4
X_resolution = 1.19959034[Hz]
X_sweep = 39.3081761[kHz]
Irr_domain = 1H
Irr_freq = 500.15991521[MHz]
Irr_offset = 5.0[ppm]
Clipped = FALSE
Mod_return = 1
Scans = 302
Total_scans = 302
X_90_width = 9.56[us]
X_acq_time = 0.83361792[s]
X_angle = 30[deg]
X_atn = 9.1[dB]
X_pulse = 3.18666667[us]
Irr_atn_dec = 21[dB]
Irr_atn_noe = 21[dB]
Irr_noise = WALTZ
Decoupling = TRUE
Initial_wait = 1[s]
Noe = TRUE
Noe_time = 2[s]
Recvr_gain = 54
Relaxation_delay = 2[s]
Repetition_time = 2.83361792[s]
Temp_get = 25.3[dC]

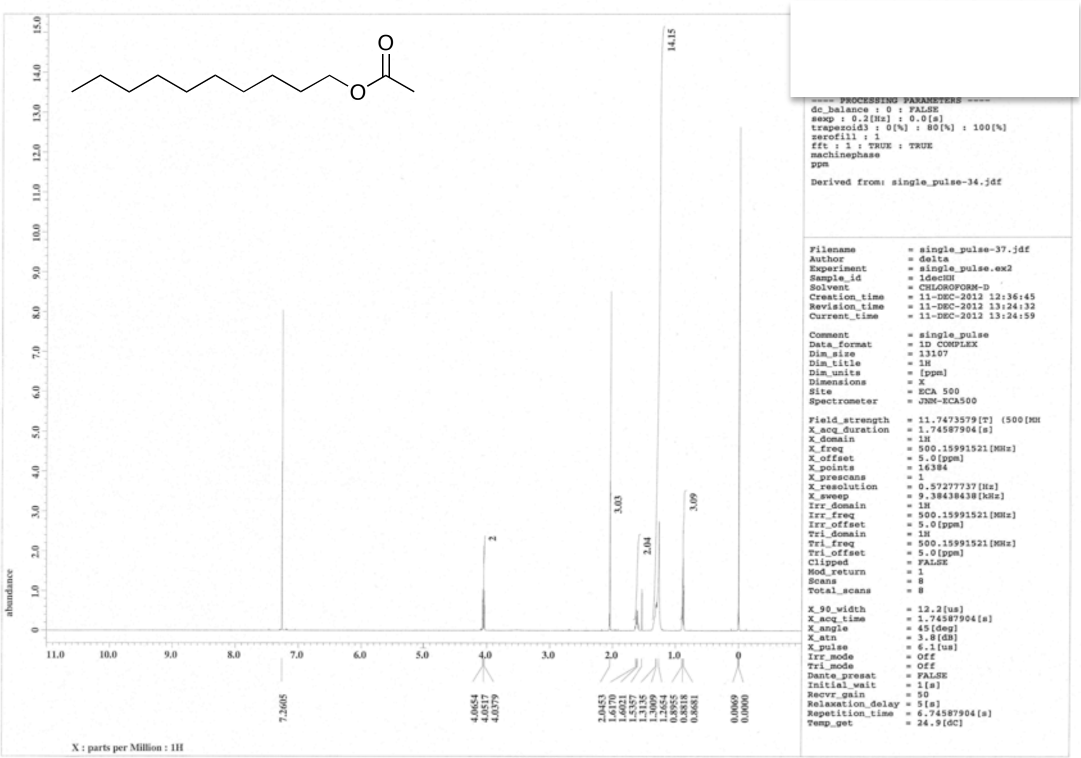

O
abundance
X : parts per Million : 1H
14.15
3.03
3.09
2.04
2
7.2605
4.0654
4.0517
4.0379
2.0453
1.6170
1.6021
1.5357
1.3135
1.3009
1.2654
0.8955
0.8818
0.8681
0.0069
0.0000
---- PROCESSING PARAMETERS ----
dc_balance : 0 : FALSE
sexp : 0.2[Hz] : 0.0[s]
trapezoid3 : 0[%] : 80[%] : 100[%]
zerofill : 1
fft : 1 : TRUE : TRUE
machinephase
ppm
Derived from: single_pulse-34.jdf
Filename = single_pulse-37.jdf
Author = delta
Experiment = single_pulse.ex2
Sample_id = 1decHH
Solvent = CHLOROFORM-D
Creation_time = 11-DEC-2012 12:36:45
Revision_time = 11-DEC-2012 13:24:32
Current_time = 11-DEC-2012 13:24:59
Comment = single_pulse
Data_format = 1D COMPLEX
Dim_size = 13107
Dim_title = 1H
Dim_units = [ppm]
Dimensions = X
Site = ECA 500
Spectrometer = JNM-ECA500
Field_strength = 11.7473579[T] (500[MH
X_acq_duration = 1.74587904[s]
X_domain = 1H
X_freq = 500.15991521[MHz]
X_offset = 5.0[ppm]
X_points = 16384
X_prescans = 1
X_resolution = 0.57277737[Hz]
X_sweep = 9.38438438[kHz]
Irr_domain = 1H
Irr_freq = 500.15991521[MHz]
Irr_offset = 5.0[ppm]
Tri_domain = 1H
Tri_freq = 500.15991521[MHz]
Tri_offset = 5.0[ppm]
Clipped = FALSE
Mod_return = 1
Scans = 8
Total_scans = 8
X_90_width = 12.2[us]
X_acq_time = 1.74587904[s]
X_angle = 45[deg]
X_atn = 3.8[dB]
X_pulse = 6.1[us]
Irr_mode = Off
Tri_mode = Off
Dante_presat = FALSE
Initial_wait = 1[s]
Recvr_gain = 50
Relaxation_delay = 5[s]
Repetition_time = 6.74587904[s]
Temp_get = 24.9[dC]

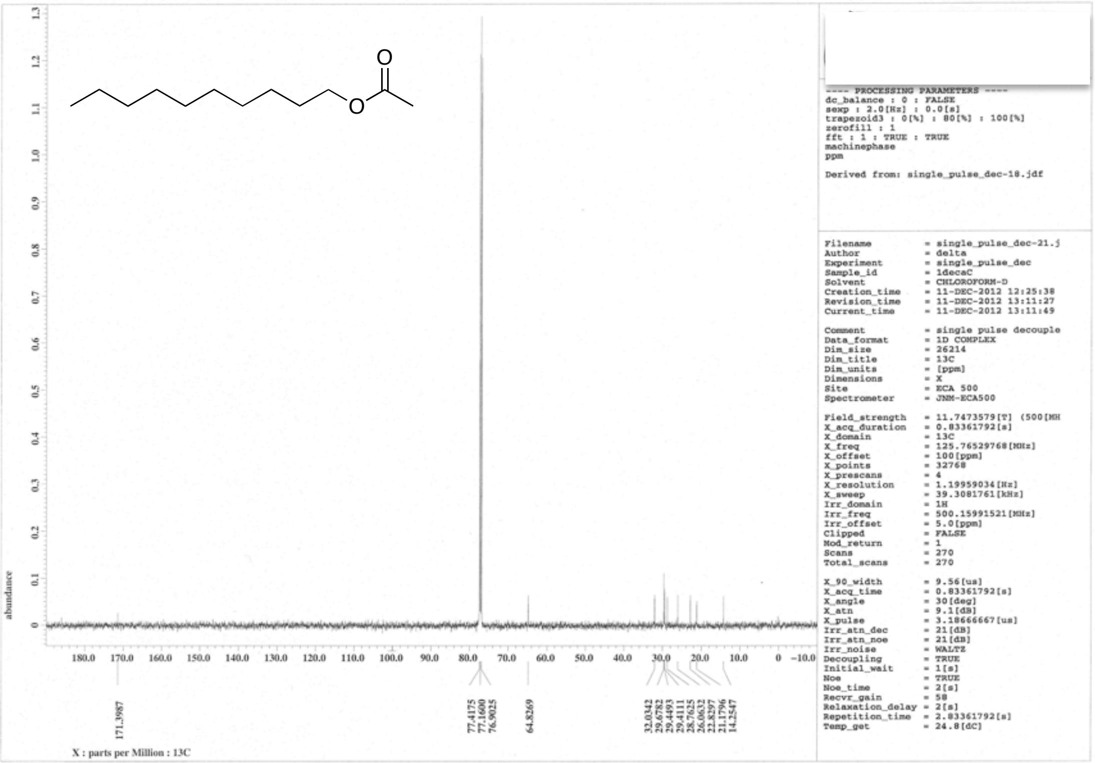

O
abundance
X : parts per Million : 13C
171.3987
77.4175
77.1600
76.9025
64.8269
32.0342
29.6782
29.4493
29.4111
28.7625
26.0632
22.8297
21.1796
14.2547
---- PROCESSING PARAMETERS ----
dc_balance : 0 : FALSE
sexp : 2.0[Hz] : 0.0[s]
trapezoid3 : 0[%] : 80[%] : 100[%]
zerofill : 1
fft : 1 : TRUE : TRUE
machinephase
ppm
Derived from: single_pulse_dec-18.jdf
Filename = single_pulse_dec-21.j
Author = delta
Experiment = single_pulse_dec
Sample_id = 1decaC
Solvent = CHLOROFORM-D
Creation_time = 11-DEC-2012 12:25:38
Revision_time = 11-DEC-2012 13:11:27
Current_time = 11-DEC-2012 13:11:49
Comment = single pulse decouple
Data_format = 1D COMPLEX
Dim_size = 26214
Dim_title = 13C
Dim_units = [ppm]
Dimensions = X
Site = ECA 500
Spectrometer = JNM-ECA500
Field_strength = 11.7473579[T] (500[MH
X_acq_duration = 0.83361792[s]
X_domain = 13C
X_freq = 125.76529768[MHz]
X_offset = 100[ppm]
X_points = 32768
X_prescans = 4
X_resolution = 1.19959034[Hz]
X_sweep = 39.3081761[kHz]
Irr_domain = 1H
Irr_freq = 500.15991521[MHz]
Irr_offset = 5.0[ppm]
Clipped = FALSE
Mod_return = 1
Scans = 270
Total_scans = 270
X_90_width = 9.56[us]
X_acq_time = 0.83361792[s]
X_angle = 30[deg]
X_atn = 9.1[dB]
X_pulse = 3.18666667[us]
Irr_atn_dec = 21[dB]
Irr_atn_noe = 21[dB]
Irr_noise = WALTZ
Decoupling = TRUE
Initial_wait = 1[s]
Noe = TRUE
Noe_time = 2[s]
Recvr_gain = 58
Relaxation_delay = 2[s]
Repetition_time = 2.83361792[s]
Temp_get = 24.8[dC]

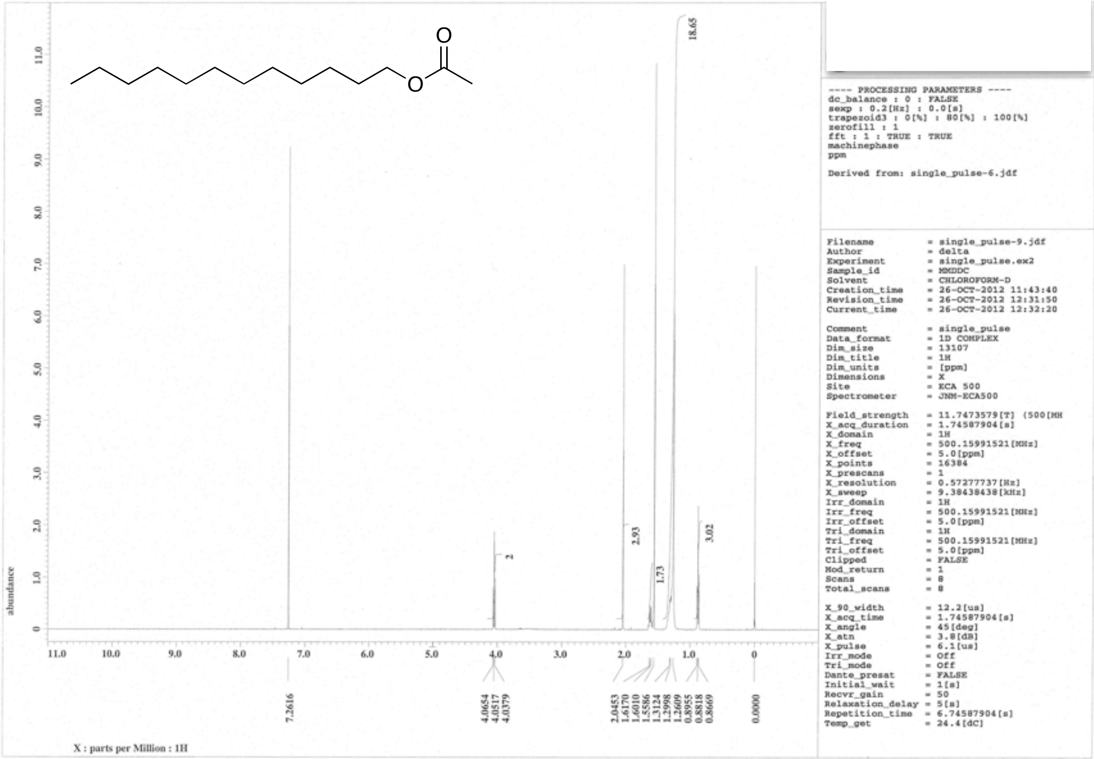
18.65
2
2.93
1.73
3.02
abundance
X : parts per Million : 1H
7.2616
4.0654
4.0517
4.0379
2.0453
1.6170
1.6010
1.5586
1.3124
1.2998
1.2609
0.8955
0.8818
0.8669
0.0000
---- PROCESSING PARAMETERS ----
dc_balance : 0 : FALSE
sexp : 0.2[Hz] : 0.0[s]
trapezoid3 : 0[%] : 80[%] : 100[%]
zerofill : 1
fft : 1 : TRUE : TRUE
machinephase
ppm
Derived from: single_pulse-6.jdf
Filename = single_pulse-9.jdf
Author = delta
Experiment = single_pulse.ex2
Sample_id = MMDDC
Solvent = CHLOROFORM-D
Creation_time = 26-OCT-2012 11:43:40
Revision_time = 26-OCT-2012 12:31:50
Current_time = 26-OCT-2012 12:32:20
Comment = single_pulse
Data_format = 1D COMPLEX
Dim_size = 13107
Dim_title = 1H
Dim_units = [ppm]
Dimensions = X
Site = ECA 500
Spectrometer = JNM-ECA500
Field_strength = 11.7473579[T] (500[MH
X_acq_duration = 1.74587904[s]
X_domain = 1H
X_freq = 500.15991521[MHz]
X_offset = 5.0[ppm]
X_points = 16384
X_prescans = 1
X_resolution = 0.57277737[Hz]
X_sweep = 9.38438438[kHz]
Irr_domain = 1H
Irr_freq = 500.15991521[MHz]
Irr_offset = 5.0[ppm]
Tri_domain = 1H
Tri_freq = 500.15991521[MHz]
Tri_offset = 5.0[ppm]
Clipped = FALSE
Mod_return = 1
Scans = 8
Total_scans = 8
X_90_width = 12.2[us]
X_acq_time = 1.74587904[s]
X_angle = 45[deg]
X_atn = 3.8[dB]
X_pulse = 6.1[us]
Irr_mode = Off
Tri_mode = Off
Dante_presat = FALSE
Initial_wait = 1[s]
Recvr_gain = 50
Relaxation_delay = 5[s]
Repetition_time = 6.74587904[s]
Temp_get = 24.4[dC]

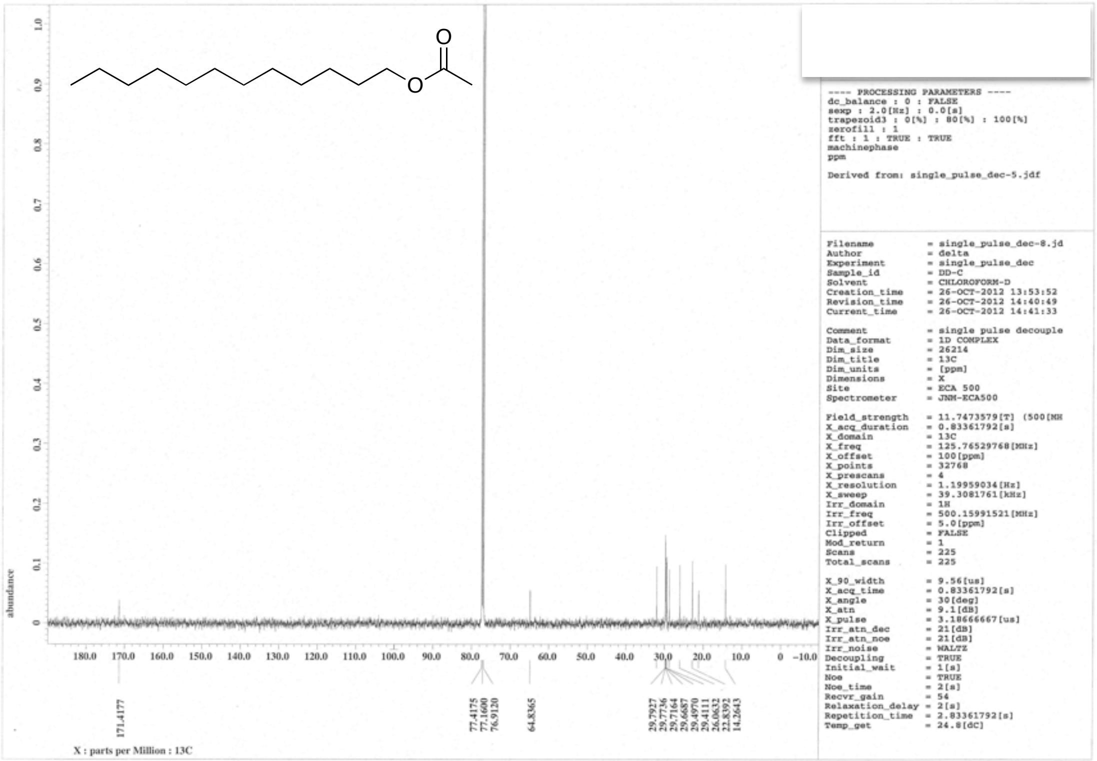
abundance
X : parts per Million : 13C
171.4177
77.4175
77.1600
76.9120
64.8365
29.7927
29.7736
29.7164
29.6687
29.4970
29.4111
26.0632
22.8392
14.2643
---- PROCESSING PARAMETERS ----
dc_balance : 0 : FALSE
sexp : 2.0[Hz] : 0.0[s]
trapezoid3 : 0[%] : 80[%] : 100[%]
zerofill : 1
fft : 1 : TRUE : TRUE
machinephase
ppm
Derived from: single_pulse_dec-5.jdf
Filename = single_pulse_dec-8.jd
Author = delta
Experiment = single_pulse_dec
Sample_id = DD-C
Solvent = CHLOROFORM-D
Creation_time = 26-OCT-2012 13:53:52
Revision_time = 26-OCT-2012 14:40:49
Current_time = 26-OCT-2012 14:41:33
Comment = single pulse decouple
Data_format = 1D COMPLEX
Dim_size = 26214
Dim_title = 13C
Dim_units = [ppm]
Dimensions = X
Site = ECA 500
Spectrometer = JNM-ECA500
Field_strength = 11.7473579[T] (500[MH
X_acq_duration = 0.83361792[s]
X_domain = 13C
X_freq = 125.76529768[MHz]
X_offset = 100[ppm]
X_points = 32768
X_prescans = 4
X_resolution = 1.19959034[Hz]
X_sweep = 39.3081761[kHz]
Irr_domain = 1H
Irr_freq = 500.15991521[MHz]
Irr_offset = 5.0[ppm]
Clipped = FALSE
Mod_return = 1
Scans = 225
Total_scans = 225
X_90_width = 9.56[us]
X_acq_time = 0.83361792[s]
X_angle = 30[deg]
X_atn = 9.1[dB]
X_pulse = 3.18666667[us]
Irr_atn_dec = 21[dB]
Irr_atn_noe = 21[dB]
Irr_noise = WALTZ
Decoupling = TRUE
Initial_wait = 1[s]
Noe = TRUE
Noe_time = 2[s]
Recvr_gain = 54
Relaxation_delay = 2[s]
Repetition_time = 2.83361792[s]
Temp_get = 24.8[dC]

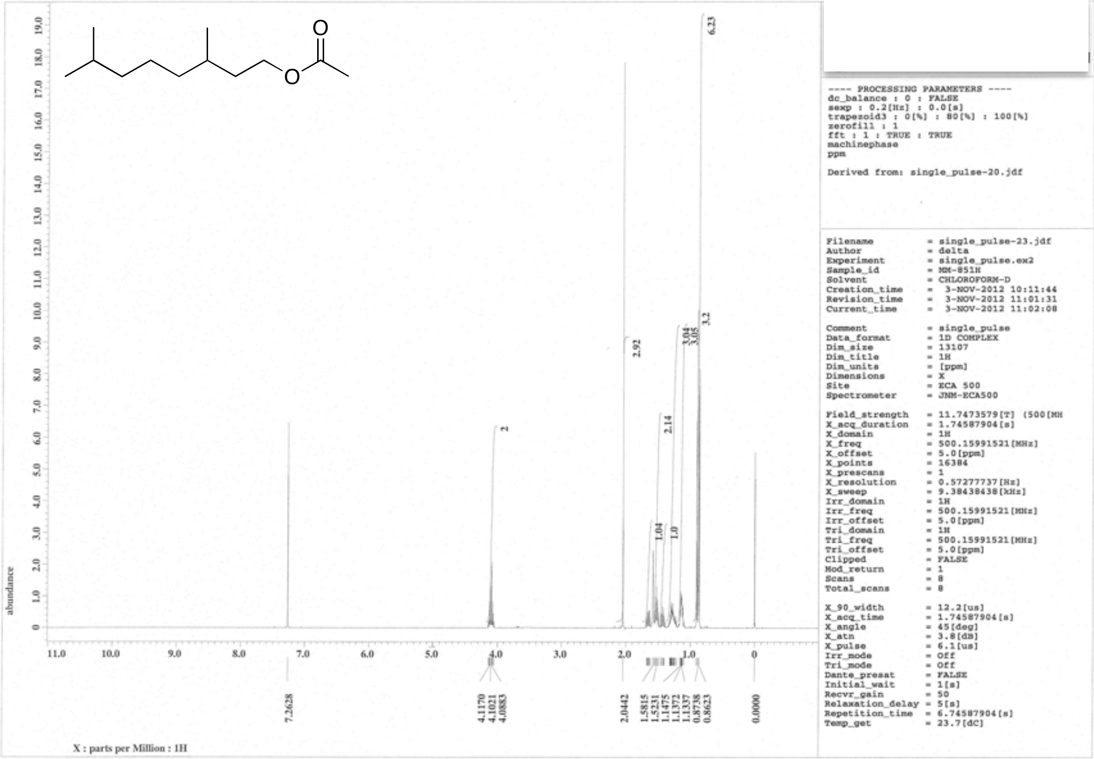
---- PROCESSING PARAMETERS ----
dc_balance : 0 : FALSE
sexp : 0.2[Hz] : 0.0[s]
trapezoid3 : 0[%] : 80[%] : 100[%]
zerofill : 1
fft : 1 : TRUE : TRUE
machinephase
ppm
Derived from: single_pulse-20.jdf
Filename = single_pulse-23.jdf
Author = delta
Experiment = single_pulse.ex2
Sample_id = MM-851H
Solvent = CHLOROFORM-D
Creation_time = 3-NOV-2012 10:11:44
Revision_time = 3-NOV-2012 11:01:31
Current_time = 3-NOV-2012 11:02:08
Comment = single_pulse
Data_format = 1D COMPLEX
Dim_size = 13107
Dim_title = 1H
Dim_units = [ppm]
Dimensions = X
Site = ECA 500
Spectrometer = JNM-ECA500
Field_strength = 11.7473579[T] (500[MH
X_acq_duration = 1.74587904[s]
X_domain = 1H
X_freq = 500.15991521[MHz]
X_offset = 5.0[ppm]
X_points = 16384
X_prescans = 1
X_resolution = 0.57277737[Hz]
X_sweep = 9.38438438[kHz]
Irr_domain = 1H
Irr_freq = 500.15991521[MHz]
Irr_offset = 5.0[ppm]
Tri_domain = 1H
Tri_freq = 500.15991521[MHz]
Tri_offset = 5.0[ppm]
Clipped = FALSE
Mod_return = 1
Scans = 8
Total_scans = 8
X_90_width = 12.2[us]
X_acq_time = 1.74587904[s]
X_angle = 45[deg]
X_atn = 3.8[dB]
X_pulse = 6.1[us]
Irr_mode = Off
Tri_mode = Off
Dante_presat = FALSE
Initial_wait = 1[s]
Recvr_gain = 50
Relaxation_delay = 5[s]
Repetition_time = 6.74587904[s]
Temp_get = 23.7[dC]
abundance
X : parts per Million : 1H
7.2628
4.1170
4.1021
4.0883
2.0442
1.5815
1.5321
1.1475
1.1372
1.1337
0.8738
0.8623
0.0000
2
2.92
2.14
1.04
1.0
3.04
3.05
3.2
6.23

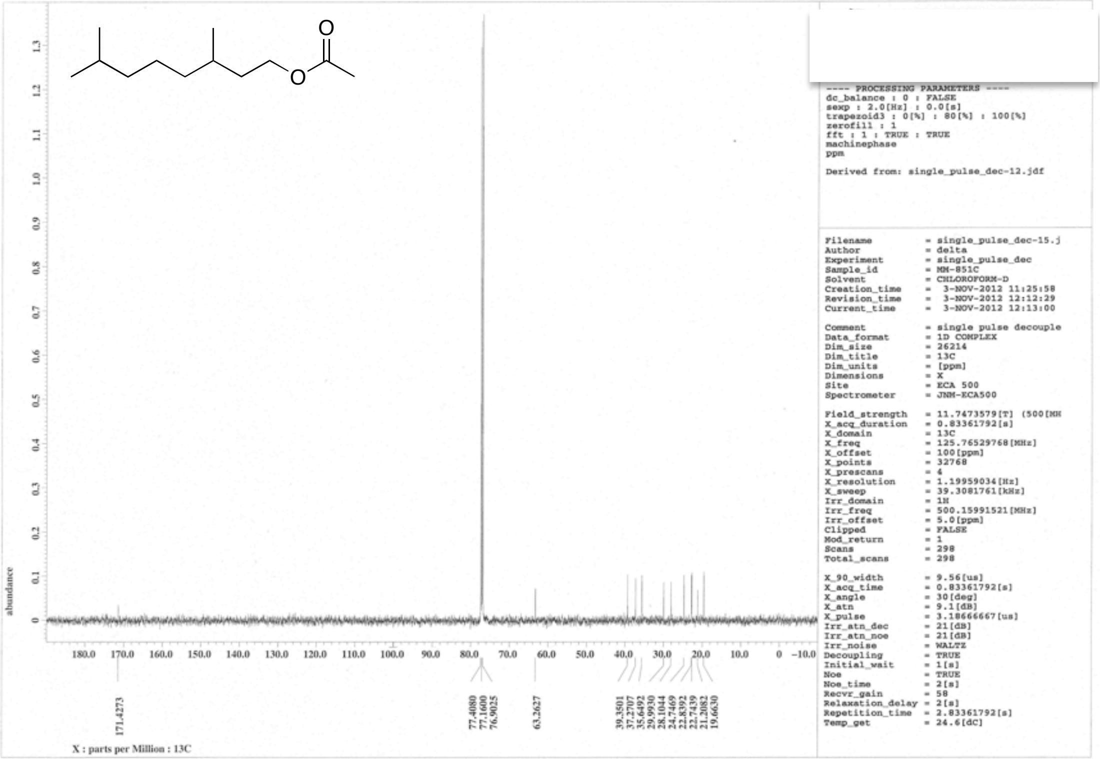
---- PROCESSING PARAMETERS ----
dc_balance : 0 : FALSE
sexp : 2.0[Hz] : 0.0[s]
trapezoid3 : 0[%] : 80[%] : 100[%]
zerofill : 1
fft : 1 : TRUE : TRUE
machinephase
ppm
Derived from: single_pulse_dec-12.jdf
Filename = single_pulse_dec-15.j
Author = delta
Experiment = single_pulse_dec
Sample_id = MM-851C
Solvent = CHLOROFORM-D
Creation_time = 3-NOV-2012 11:25:58
Revision_time = 3-NOV-2012 12:12:29
Current_time = 3-NOV-2012 12:13:00
Comment = single pulse decouple
Data_format = 1D COMPLEX
Dim_size = 26214
Dim_title = 13C
Dim_units = [ppm]
Dimensions = X
Site = ECA 500
Spectrometer = JNM-ECA500
Field_strength = 11.7473579[T] (500[MH
X_acq_duration = 0.83361792[s]
X_domain = 13C
X_freq = 125.76529768[MHz]
X_offset = 100[ppm]
X_points = 32768
X_prescans = 4
X_resolution = 1.19959034[Hz]
X_sweep = 39.3081761[kHz]
Irr_domain = 1H
Irr_freq = 500.15991521[MHz]
Irr_offset = 5.0[ppm]
Clipped = FALSE
Mod_return = 1
Scans = 298
Total_scans = 298
X_90_width = 9.56[us]
X_acq_time = 0.83361792[s]
X_angle = 30[deg]
X_atn = 9.1[dB]
X_pulse = 3.18666667[us]
Irr_atn_dec = 21[dB]
Irr_atn_noe = 21[dB]
Irr_noise = WALTZ
Decoupling = TRUE
Initial_wait = 1[s]
Noe = TRUE
Noe_time = 2[s]
Recvr_gain = 58
Relaxation_delay = 2[s]
Repetition_time = 2.83361792[s]
Temp_get = 24.6[dC]
abundance
X : parts per Million : 13C
171.4273
77.4080
77.1600
76.9025
63.2627
39.3501
37.2707
35.6492
29.9930
28.1044
24.7469
22.8392
22.7439
21.2082
19.6630

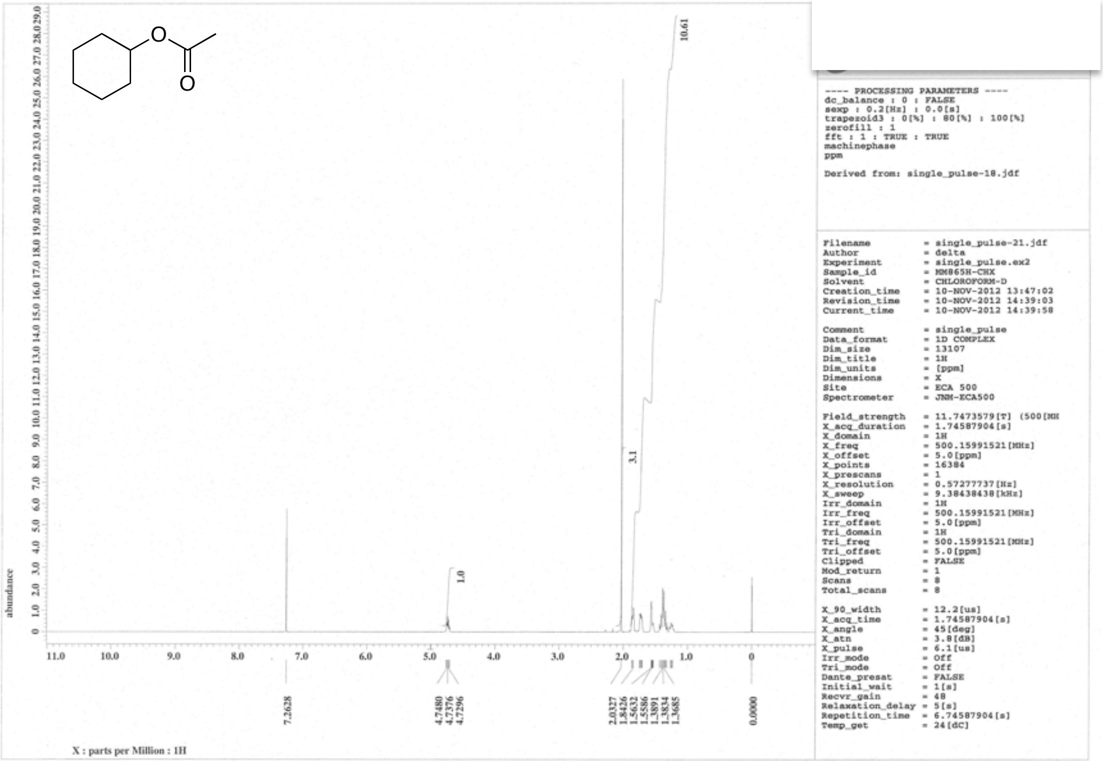
O
O
10.61
3.1
1.0
abundance
X : parts per Million : 1H
7.2628
4.7480
4.7376
4.7296
2.0327
1.8426
1.5632
1.5586
1.3891
1.3834
1.3685
0.0000
---- PROCESSING PARAMETERS ----
dc_balance : 0 : FALSE
sexp : 0.2[Hz] : 0.0[s]
trapezoid3 : 0[%] : 80[%] : 100[%]
zerofill : 1
fft : 1 : TRUE : TRUE
machinephase
ppm
Derived from: single_pulse-18.jdf
Filename = single_pulse-21.jdf
Author = delta
Experiment = single_pulse.ex2
Sample_id = MM865H-CHX
Solvent = CHLOROFORM-D
Creation_time = 10-NOV-2012 13:47:02
Revision_time = 10-NOV-2012 14:39:03
Current_time = 10-NOV-2012 14:39:58
Comment = single_pulse
Data_format = 1D COMPLEX
Dim_size = 13107
Dim_title = 1H
Dim_units = [ppm]
Dimensions = X
Site = ECA 500
Spectrometer = JNM-ECA500
Field_strength = 11.7473579[T] (500[MH
X_acq_duration = 1.74587904[s]
X_domain = 1H
X_freq = 500.15991521[MHz]
X_offset = 5.0[ppm]
X_points = 16384
X_prescans = 1
X_resolution = 0.57277737[Hz]
X_sweep = 9.38438438[kHz]
Irr_domain = 1H
Irr_freq = 500.15991521[MHz]
Irr_offset = 5.0[ppm]
Tri_domain = 1H
Tri_freq = 500.15991521[MHz]
Tri_offset = 5.0[ppm]
Clipped = FALSE
Mod_return = 1
Scans = 8
Total_scans = 8
X_90_width = 12.2[us]
X_acq_time = 1.74587904[s]
X_angle = 45[deg]
X_atn = 3.8[dB]
X_pulse = 6.1[us]
Irr_mode = Off
Tri_mode = Off
Dante_presat = FALSE
Initial_wait = 1[s]
Recvr_gain = 48
Relaxation_delay = 5[s]
Repetition_time = 6.74587904[s]
Temp_get = 24[dC]

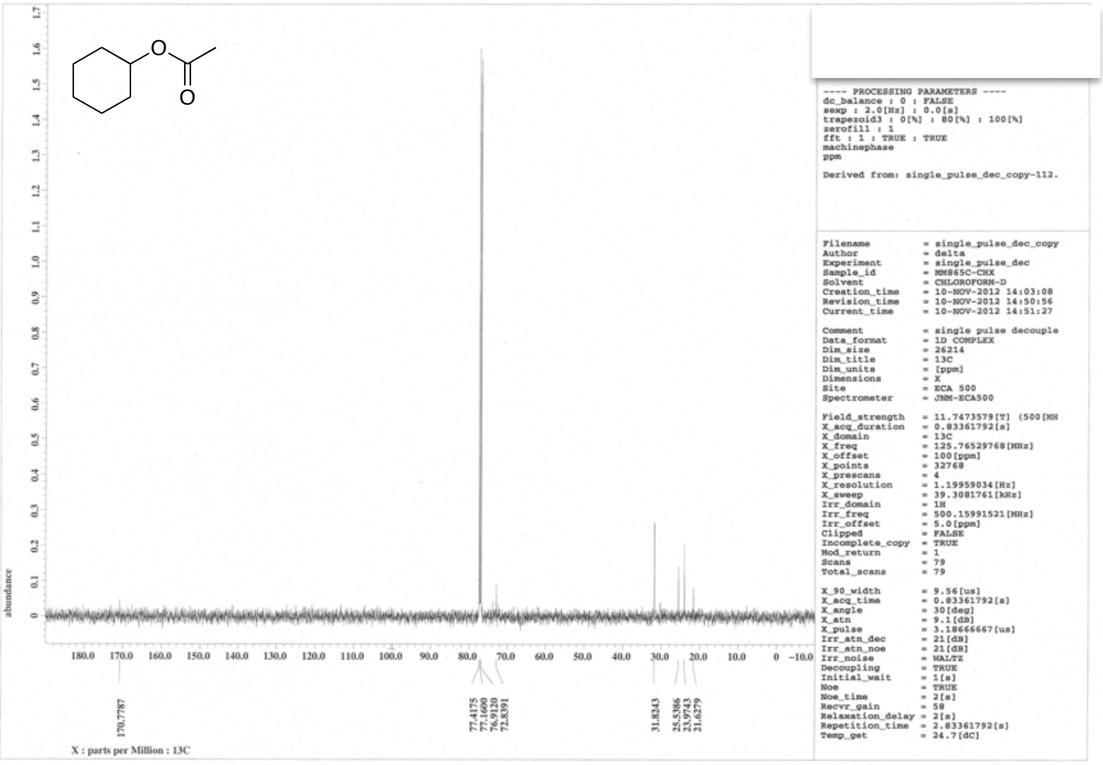
O
O
abundance
X : parts per Million : 13C
170.7787
77.4175
77.1600
76.9120
72.8391
31.8243
25.5386
23.9743
21.6279
---- PROCESSING PARAMETERS ----
dc_balance : 0 : FALSE
sexp : 2.0[Hz] : 0.0[s]
trapezoid3 : 0[%] : 80[%] : 100[%]
zerofill : 1
fft : 1 : TRUE : TRUE
machinephase
ppm
Derived from: single_pulse_dec_copy-112.
Filename = single_pulse_dec_copy
Author = delta
Experiment = single_pulse_dec
Sample_id = MM865C-CHX
Solvent = CHLOROFORM-D
Creation_time = 10-NOV-2012 14:03:08
Revision_time = 10-NOV-2012 14:50:56
Current_time = 10-NOV-2012 14:51:27
Comment = single pulse decouple
Data_format = 1D COMPLEX
Dim_size = 26214
Dim_title = 13C
Dim_units = [ppm]
Dimensions = X
Site = ECA 500
Spectrometer = JNM-ECA500
Field_strength = 11.7473579[T] (500[MH
X_acq_duration = 0.83361792[s]
X_domain = 13C
X_freq = 125.76529768[MHz]
X_offset = 100[ppm]
X_points = 32768
X_prescans = 4
X_resolution = 1.19959034[Hz]
X_sweep = 39.3081761[kHz]
Irr_domain = 1H
Irr_freq = 500.15991521[MHz]
Irr_offset = 5.0[ppm]
Clipped = FALSE
Incomplete_copy = TRUE
Mod_return = 1
Scans = 79
Total_scans = 79
X_90_width = 9.56[us]
X_acq_time = 0.83361792[s]
X_angle = 30[deg]
X_atn = 9.1[dB]
X_pulse = 3.18666667[us]
Irr_atn_dec = 21[dB]
Irr_atn_noe = 21[dB]
Irr_noise = WALTZ
Decoupling = TRUE
Initial_wait = 1[s]
Noe = TRUE
Noe_time = 2[s]
Recvr_gain = 58
Relaxation_delay = 2[s]
Repetition_time = 2.83361792[s]
Temp_get = 24.7[dC]

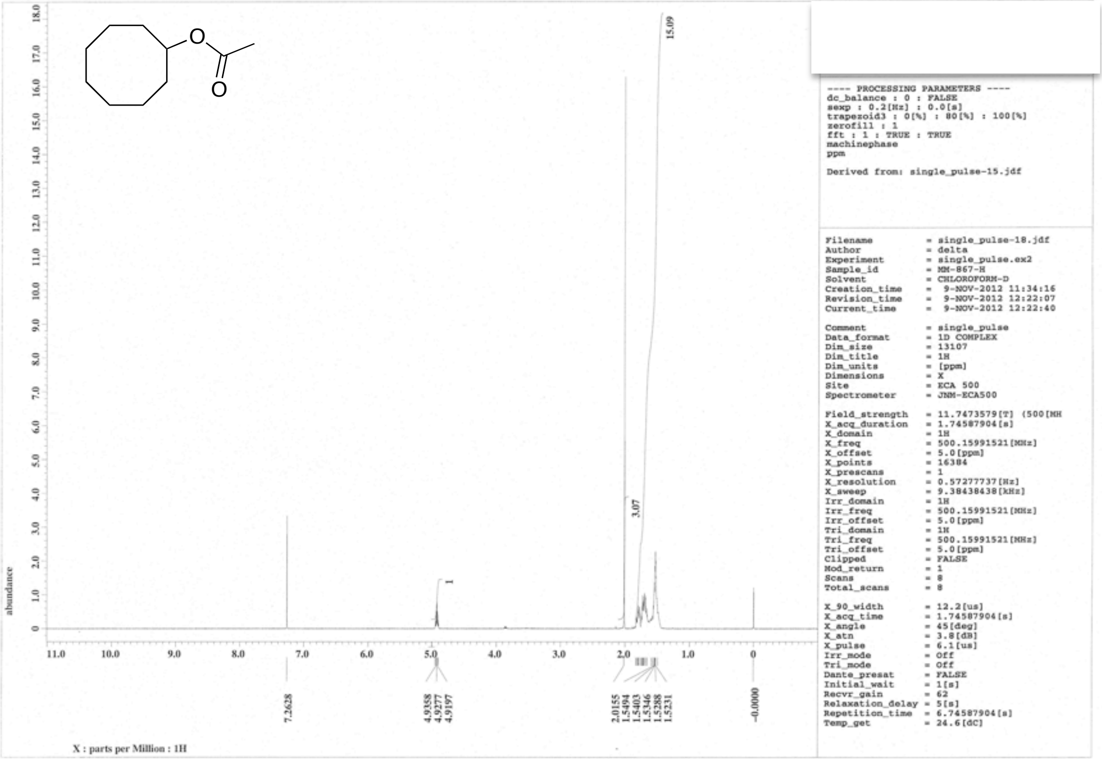
O
O
15.09
3.07
1
---- PROCESSING PARAMETERS ----
dc_balance : 0 : FALSE
sexp : 0.2[Hz] : 0.0[s]
trapezoid3 : 0[%] : 80[%] : 100[%]
zerofill : 1
fft : 1 : TRUE : TRUE
machinephase
ppm
Derived from: single_pulse-15.jdf
Filename = single_pulse-18.jdf
Author = delta
Experiment = single_pulse.ex2
Sample_id = MM-867-H
Solvent = CHLOROFORM-D
Creation_time = 9-NOV-2012 11:34:16
Revision_time = 9-NOV-2012 12:22:07
Current_time = 9-NOV-2012 12:22:40
Comment = single_pulse
Data_format = 1D COMPLEX
Dim_size = 13107
Dim_title = 1H
Dim_units = [ppm]
Dimensions = X
Site = ECA 500
Spectrometer = JNM-ECA500
Field_strength = 11.7473579[T] (500[MH
X_acq_duration = 1.74587904[s]
X_domain = 1H
X_freq = 500.15991521[MHz]
X_offset = 5.0[ppm]
X_points = 16384
X_prescans = 1
X_resolution = 0.57277737[Hz]
X_sweep = 9.38438438[kHz]
Irr_domain = 1H
Irr_freq = 500.15991521[MHz]
Irr_offset = 5.0[ppm]
Tri_domain = 1H
Tri_freq = 500.15991521[MHz]
Tri_offset = 5.0[ppm]
Clipped = FALSE
Mod_return = 1
Scans = 8
Total_scans = 8
X_90_width = 12.2[us]
X_acq_time = 1.74587904[s]
X_angle = 45[deg]
X_atn = 3.8[dB]
X_pulse = 6.1[us]
Irr_mode = Off
Tri_mode = Off
Dante_presat = FALSE
Initial_wait = 1[s]
Recvr_gain = 62
Relaxation_delay = 5[s]
Repetition_time = 6.74587904[s]
Temp_get = 24.6[dC]
abundance
18.0 17.0 16.0 15.0 14.0 13.0 12.0 11.0 10.0 9.0 8.0 7.0 6.0 5.0 4.0 3.0 2.0 1.0 0
11.0 10.0 9.0 8.0 7.0 6.0 5.0 4.0 3.0 2.0 1.0 0
7.2628
4.9358
4.9277
4.9197
2.0155
1.5494
1.5403
1.5346
1.5288
1.5231
-0.0000
X : parts per Million : 1H

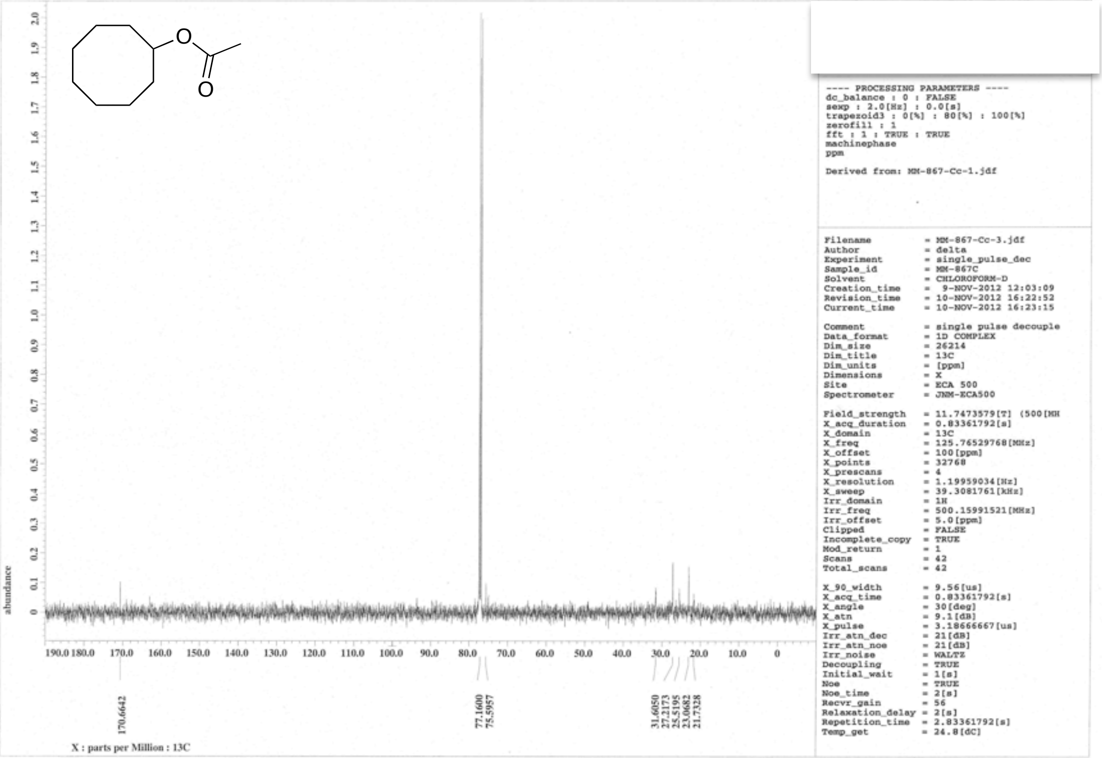
O
O
---- PROCESSING PARAMETERS ----
dc_balance : 0 : FALSE
sexp : 2.0[Hz] : 0.0[s]
trapezoid3 : 0[%] : 80[%] : 100[%]
zerofill : 1
fft : 1 : TRUE : TRUE
machinephase
ppm
Derived from: MM-867-Cc-1.jdf
Filename = MM-867-Cc-3.jdf
Author = delta
Experiment = single_pulse_dec
Sample_id = MM-867C
Solvent = CHLOROFORM-D
Creation_time = 9-NOV-2012 12:03:09
Revision_time = 10-NOV-2012 16:22:52
Current_time = 10-NOV-2012 16:23:15
Comment = single pulse decouple
Data_format = 1D COMPLEX
Dim_size = 26214
Dim_title = 13C
Dim_units = [ppm]
Dimensions = X
Site = ECA 500
Spectrometer = JNM-ECA500
Field_strength = 11.7473579[T] (500[MH
X_acq_duration = 0.83361792[s]
X_domain = 13C
X_freq = 125.76529768[MHz]
X_offset = 100[ppm]
X_points = 32768
X_prescans = 4
X_resolution = 1.19959034[Hz]
X_sweep = 39.3081761[kHz]
Irr_domain = 1H
Irr_freq = 500.15991521[MHz]
Irr_offset = 5.0[ppm]
Clipped = FALSE
Incomplete_copy = TRUE
Mod_return = 1
Scans = 42
Total_scans = 42
X_90_width = 9.56[us]
X_acq_time = 0.83361792[s]
X_angle = 30[deg]
X_atn = 9.1[dB]
X_pulse = 3.18666667[us]
Irr_atn_dec = 21[dB]
Irr_atn_noe = 21[dB]
Irr_noise = WALTZ
Decoupling = TRUE
Initial_wait = 1[s]
Noe = TRUE
Noe_time = 2[s]
Recvr_gain = 56
Relaxation_delay = 2[s]
Repetition_time = 2.83361792[s]
Temp_get = 24.8[dC]
abundance
2.0 1.9 1.8 1.7 1.6 1.5 1.4 1.3 1.2 1.1 1.0 0.9 0.8 0.7 0.6 0.5 0.4 0.3 0.2 0.1 0
190.0 180.0 170.0 160.0 150.0 140.0 130.0 120.0 110.0 100.0 90.0 80.0 70.0 60.0 50.0 40.0 30.0 20.0 10.0 0
170.6642
77.1600
75.5957
31.6050
27.2173
25.5195
23.0682
21.7328
X : parts per Million : 13C

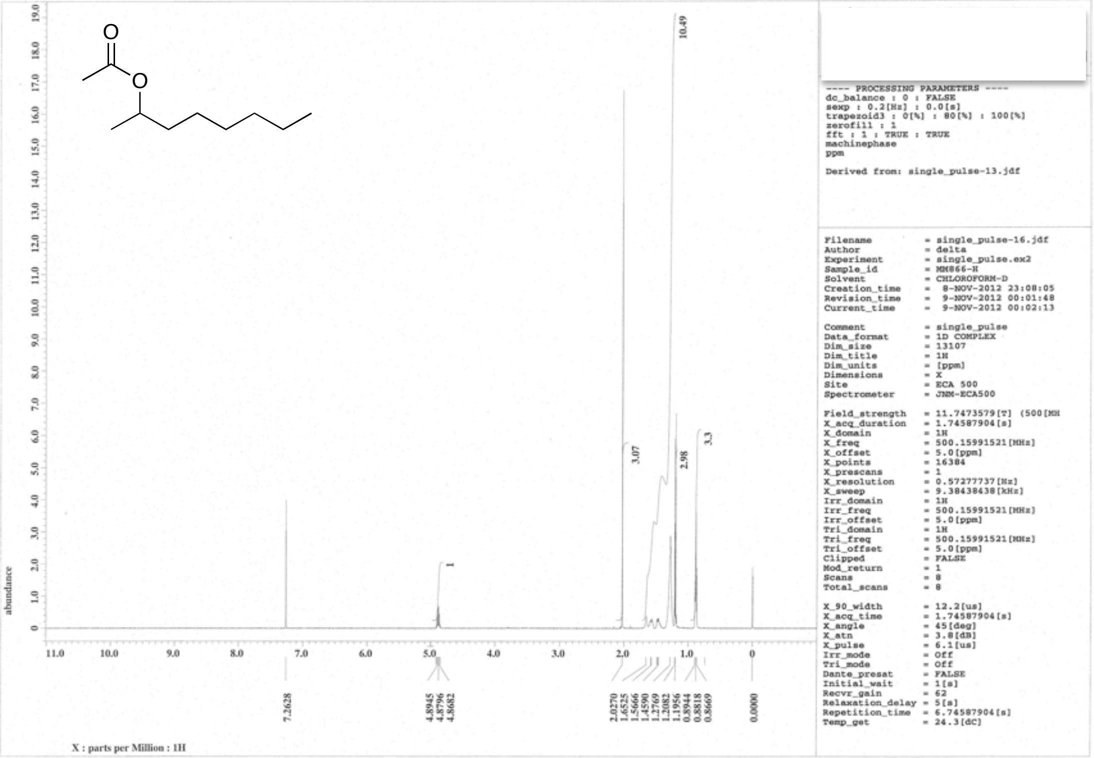

O
O
10.49
3.07
2.98
3.3
1
abundance
X : parts per Million : 1H
7.2628
4.8945
4.8796
4.8682
2.0270
1.6525
1.5666
1.4590
1.2769
1.2082
1.1956
0.8944
0.8818
0.8669
0.0000
---- PROCESSING PARAMETERS ----
dc_balance : 0 : FALSE
sexp : 0.2[Hz] : 0.0[s]
trapezoid3 : 0[%] : 80[%] : 100[%]
zerofill : 1
fft : 1 : TRUE : TRUE
machinephase
ppm
Derived from: single_pulse-13.jdf
Filename = single_pulse-16.jdf
Author = delta
Experiment = single_pulse.ex2
Sample_id = MM866-H
Solvent = CHLOROFORM-D
Creation_time = 8-NOV-2012 23:08:05
Revision_time = 9-NOV-2012 00:01:48
Current_time = 9-NOV-2012 00:02:13
Comment = single_pulse
Data_format = 1D COMPLEX
Dim_size = 13107
Dim_title = 1H
Dim_units = [ppm]
Dimensions = X
Site = ECA 500
Spectrometer = JNM-ECA500
Field_strength = 11.7473579[T] (500[MH
X_acq_duration = 1.74587904[s]
X_domain = 1H
X_freq = 500.15991521[MHz]
X_offset = 5.0[ppm]
X_points = 16384
X_prescans = 1
X_resolution = 0.57277737[Hz]
X_sweep = 9.38438438[kHz]
Irr_domain = 1H
Irr_freq = 500.15991521[MHz]
Irr_offset = 5.0[ppm]
Tri_domain = 1H
Tri_freq = 500.15991521[MHz]
Tri_offset = 5.0[ppm]
Clipped = FALSE
Mod_return = 1
Scans = 8
Total_scans = 8
X_90_width = 12.2[us]
X_acq_time = 1.74587904[s]
X_angle = 45[deg]
X_atn = 3.8[dB]
X_pulse = 6.1[us]
Irr_mode = Off
Tri_mode = Off
Dante_presat = FALSE
Initial_wait = 1[s]
Recvr_gain = 62
Relaxation_delay = 5[s]
Repetition_time = 6.74587904[s]
Temp_get = 24.3[dC]

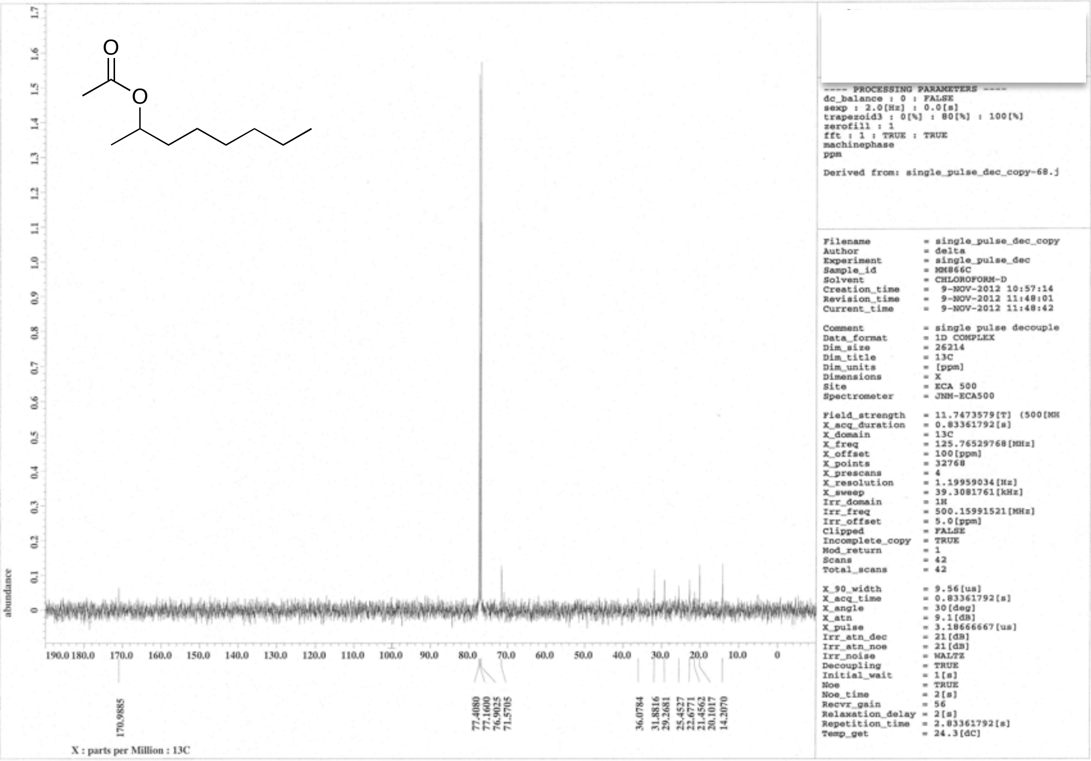

O
O
abundance
X : parts per Million : 13C
170.9885
77.4080
77.1600
76.9025
71.5705
36.0784
31.8816
29.2681
25.4527
22.6771
21.4562
20.1017
14.2070
---- PROCESSING PARAMETERS ----
dc_balance : 0 : FALSE
sexp : 2.0[Hz] : 0.0[s]
trapezoid3 : 0[%] : 80[%] : 100[%]
zerofill : 1
fft : 1 : TRUE : TRUE
machinephase
ppm
Derived from: single_pulse_dec_copy-68.j
Filename = single_pulse_dec_copy
Author = delta
Experiment = single_pulse_dec
Sample_id = MM866C
Solvent = CHLOROFORM-D
Creation_time = 9-NOV-2012 10:57:14
Revision_time = 9-NOV-2012 11:48:01
Current_time = 9-NOV-2012 11:48:42
Comment = single pulse decouple
Data_format = 1D COMPLEX
Dim_size = 26214
Dim_title = 13C
Dim_units = [ppm]
Dimensions = X
Site = ECA 500
Spectrometer = JNM-ECA500
Field_strength = 11.7473579[T] (500[MH
X_acq_duration = 0.83361792[s]
X_domain = 13C
X_freq = 125.76529768[MHz]
X_offset = 100[ppm]
X_points = 32768
X_prescans = 4
X_resolution = 1.19959034[Hz]
X_sweep = 39.3081761[kHz]
Irr_domain = 1H
Irr_freq = 500.15991521[MHz]
Irr_offset = 5.0[ppm]
Clipped = FALSE
Incomplete_copy = TRUE
Mod_return = 1
Scans = 42
Total_scans = 42
X_90_width = 9.56[us]
X_acq_time = 0.83361792[s]
X_angle = 30[deg]
X_atn = 9.1[dB]
X_pulse = 3.18666667[us]
Irr_atn_dec = 21[dB]
Irr_atn_noe = 21[dB]
Irr_noise = WALTZ
Decoupling = TRUE
Initial_wait = 1[s]
Noe = TRUE
Noe_time = 2[s]
Recvr_gain = 56
Relaxation_delay = 2[s]
Repetition_time = 2.83361792[s]
Temp_get = 24.3[dC]

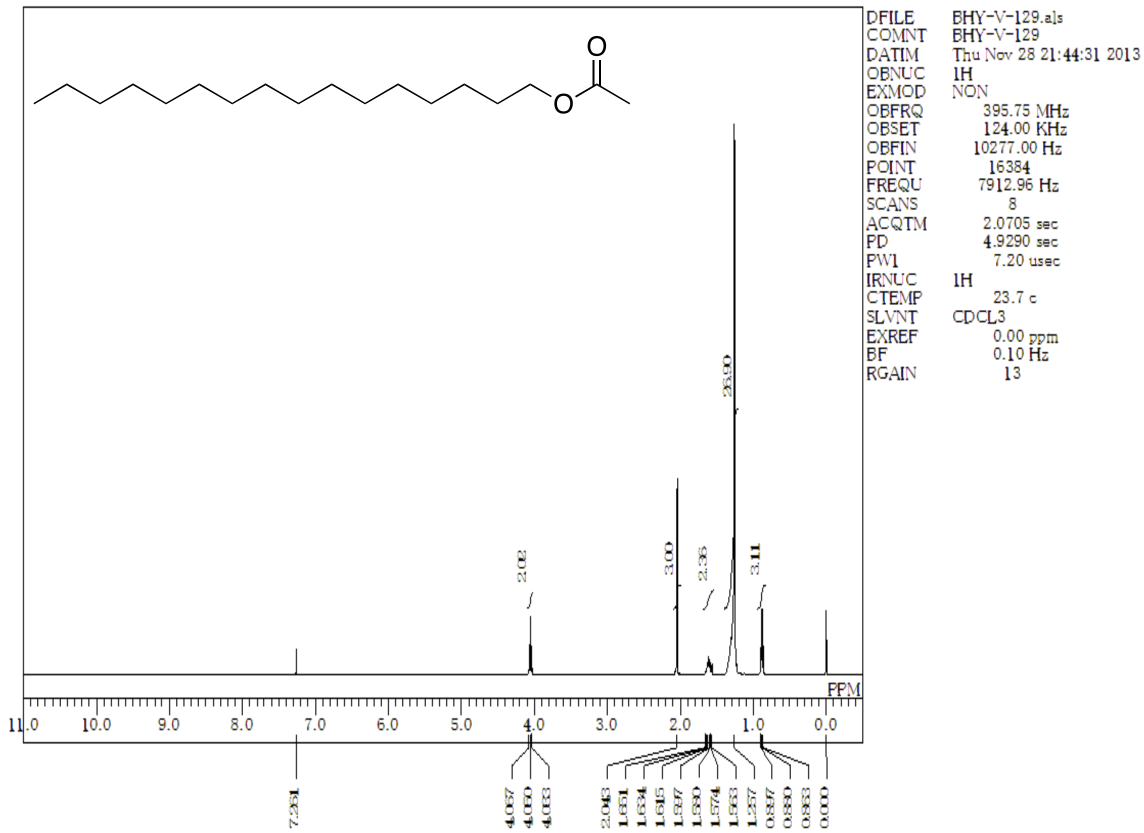
DFILE BHY-V-129.als
COMNT BHY-V-129
DATIM Thu Nov 28 21:44:31 2013
OBNUC 1H
EXMOD NON
OBFRQ 395.75 MHz
OBSET 124.00 KHz
OBFIN 10277.00 Hz
POINT 16384
FREQU 7912.96 Hz
SCANS 8
ACQTM 2.0705 sec
PD 4.9290 sec
PW1 7.20 usec
IRNUC 1H
CTEMP 23.7 c
SLVNT CDCL3
EXREF 0.00 ppm
BF 0.10 Hz
RGAIN 13
PPM

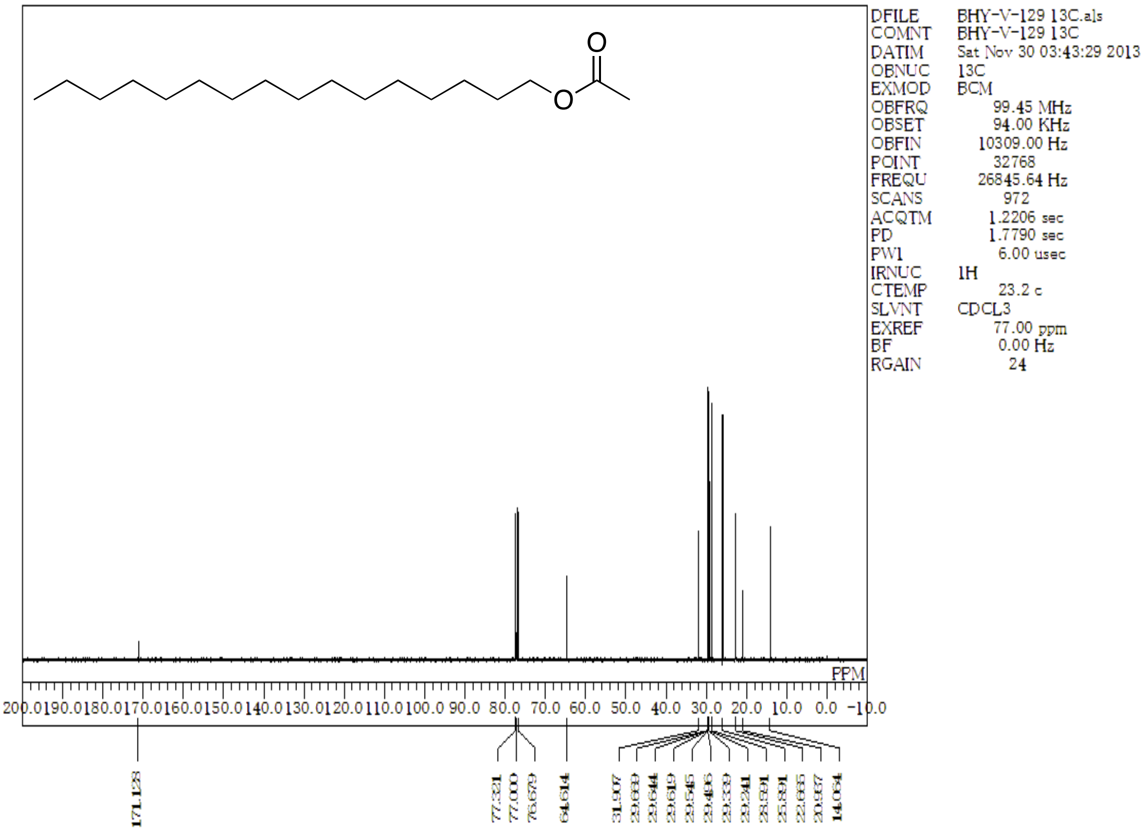
DFILE BHY-V-129 13C.als
COMNT BHY-V-129 13C
DATIM Sat Nov 30 03:43:29 2013
OBNUC 13C
EXMOD BCM
OBFRQ 99.45 MHz
OBSET 94.00 KHz
OBFIN 10309.00 Hz
POINT 32768
FREQU 26845.64 Hz
SCANS 972
ACQTM 1.2206 sec
PD 1.7790 sec
PW1 6.00 usec
IRNUC 1H
CTEMP 23.2 c
SLVNT CDCL3
EXREF 77.00 ppm
BF 0.00 Hz
RGAIN 24
PPM

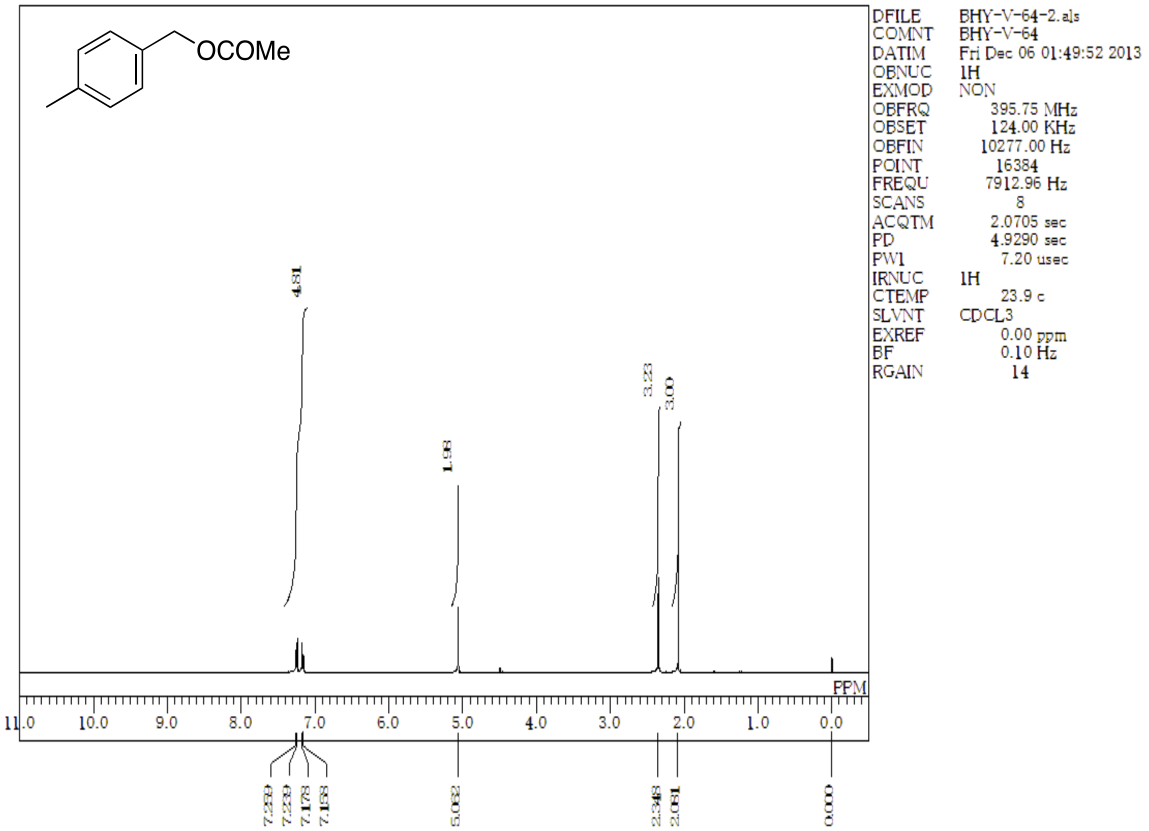
OCOMe
DFILE BHY-V-64-2.als
COMNT BHY-V-64
DATIM Fri Dec 06 01:49:52 2013
OBNUC 1H
EXMOD NON
OBFRQ 395.75 MHz
OBSET 124.00 KHz
OBFIN 10277.00 Hz
POINT 16384
FREQU 7912.96 Hz
SCANS 8
ACQTM 2.0705 sec
PD 4.9290 sec
PW1 7.20 usec
IRNUC 1H
CTEMP 23.9 c
SLVNT CDCL3
EXREF 0.00 ppm
BF 0.10 Hz
RGAIN 14
4.81
1.98
3.23
3.00
PPM
7.259
7.239
7.178
7.158
5.062
2.348
2.081
0.000

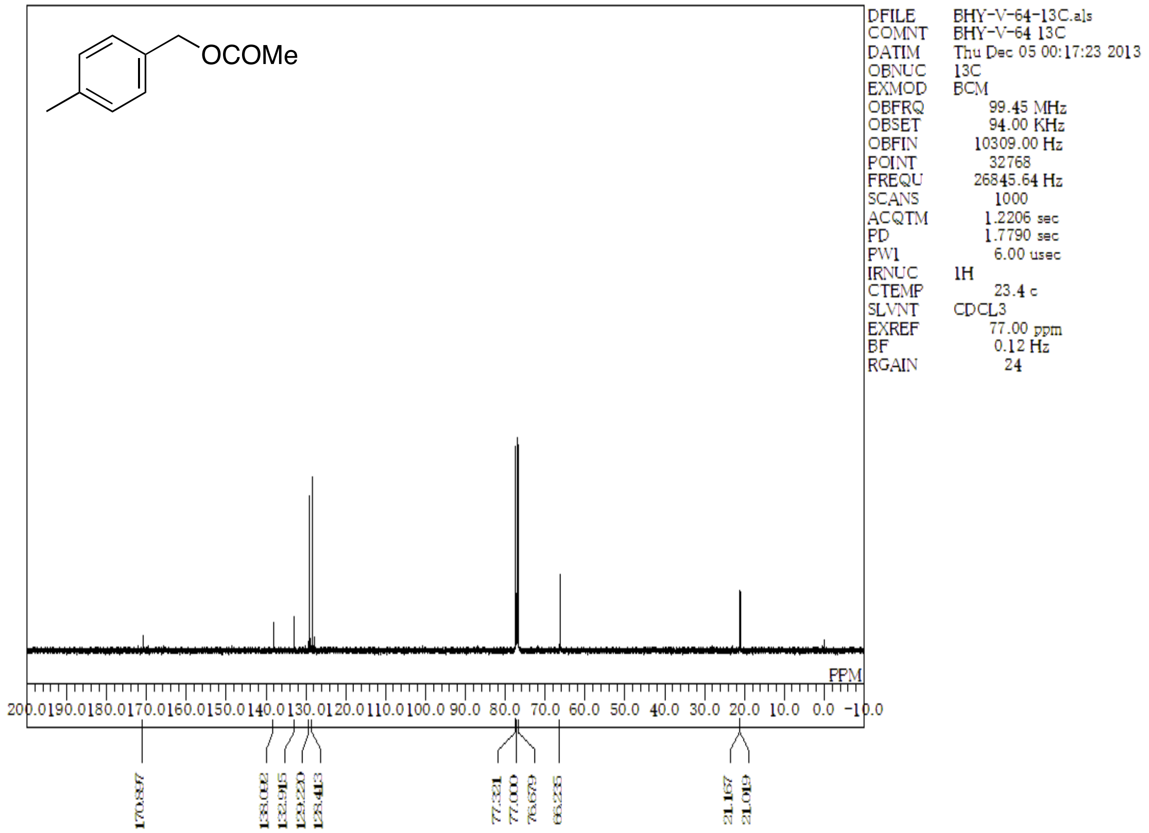
OCOMe
DFILE BHY-V-64-13C.als
COMNT BHY-V-64 13C
DATIM Thu Dec 05 00:17:23 2013
OBNUC 13C
EXMOD BCM
OBFRQ 99.45 MHz
OBSET 94.00 KHz
OBFIN 10309.00 Hz
POINT 32768
FREQU 26845.64 Hz
SCANS 1000
ACQTM 1.2206 sec
PD 1.7790 sec
PW1 6.00 usec
IRNUC 1H
CTEMP 23.4 c
SLVNT CDCL3
EXREF 77.00 ppm
BF 0.12 Hz
RGAIN 24
PPM
170.897
138.002
132.915
129.220
128.413
77.321
77.000
76.679
66.235
21.167
21.019

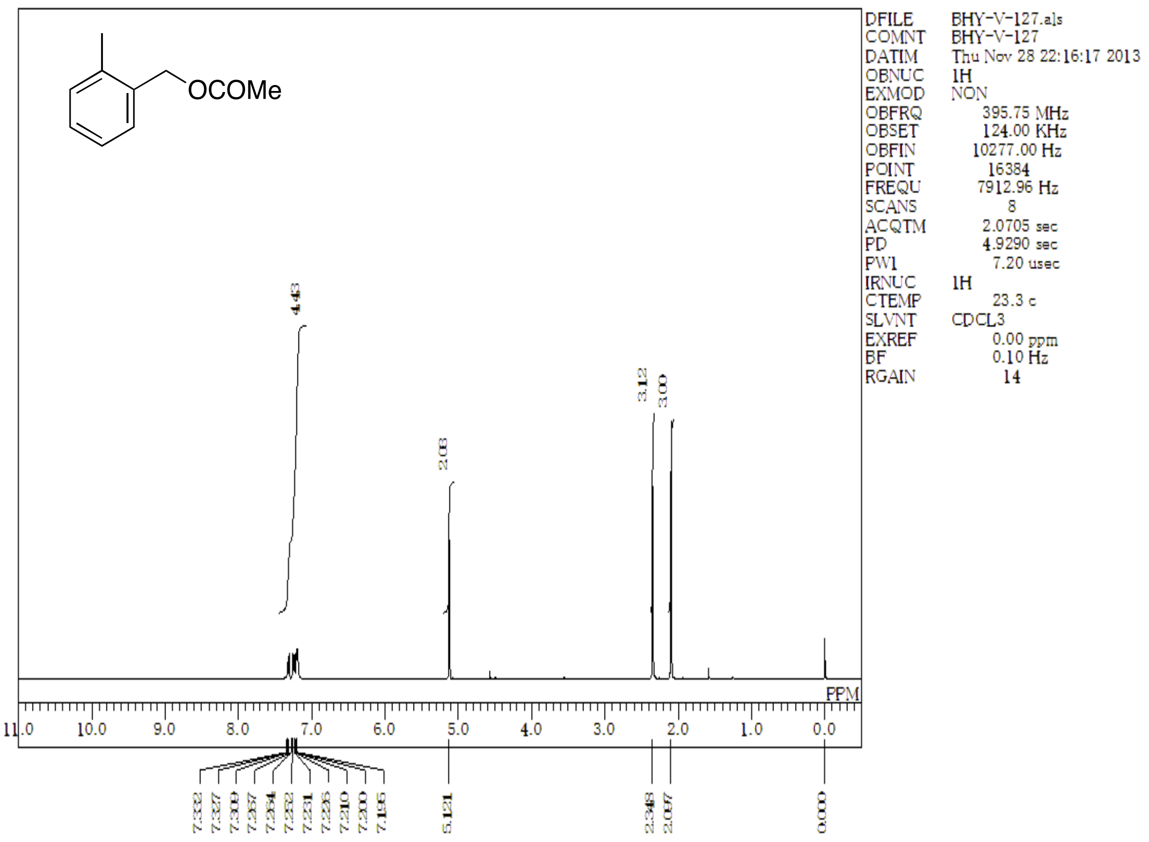
OCOMe
DFILE BHY-V-127.als
COMNT BHY-V-127
DATIM Thu Nov 28 22:16:17 2013
OBNUC 1H
EXMOD NON
OBFRQ 395.75 MHz
OBSET 124.00 KHz
OBFIN 10277.00 Hz
POINT 16384
FREQU 7912.96 Hz
SCANS 8
ACQTM 2.0705 sec
PD 4.9290 sec
PW1 7.20 usec
IRNUC 1H
CTEMP 23.3 c
SLVNT CDCL3
EXREF 0.00 ppm
BF 0.10 Hz
RGAIN 14
PPM

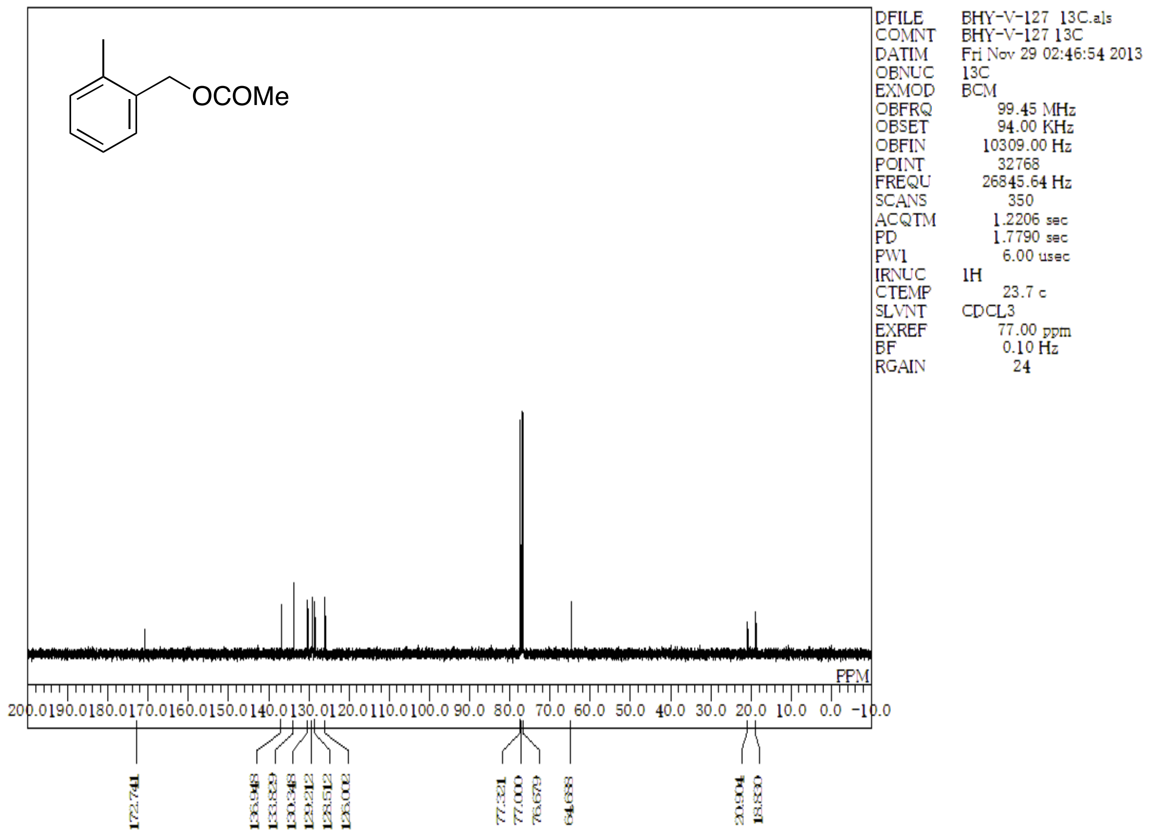
OCOMe
DFILE BHY-V-127 13C.als
COMNT BHY-V-127 13C
DATIM Fri Nov 29 02:46:54 2013
OBNUC 13C
EXMOD BCM
OBFRQ 99.45 MHz
OBSET 94.00 KHz
OBFIN 10309.00 Hz
POINT 32768
FREQU 26845.64 Hz
SCANS 350
ACQTM 1.2206 sec
PD 1.7790 sec
PW1 6.00 usec
IRNUC 1H
CTEMP 23.7 c
SLVNT CDCL3
EXREF 77.00 ppm
BF 0.10 Hz
RGAIN 24
PPM

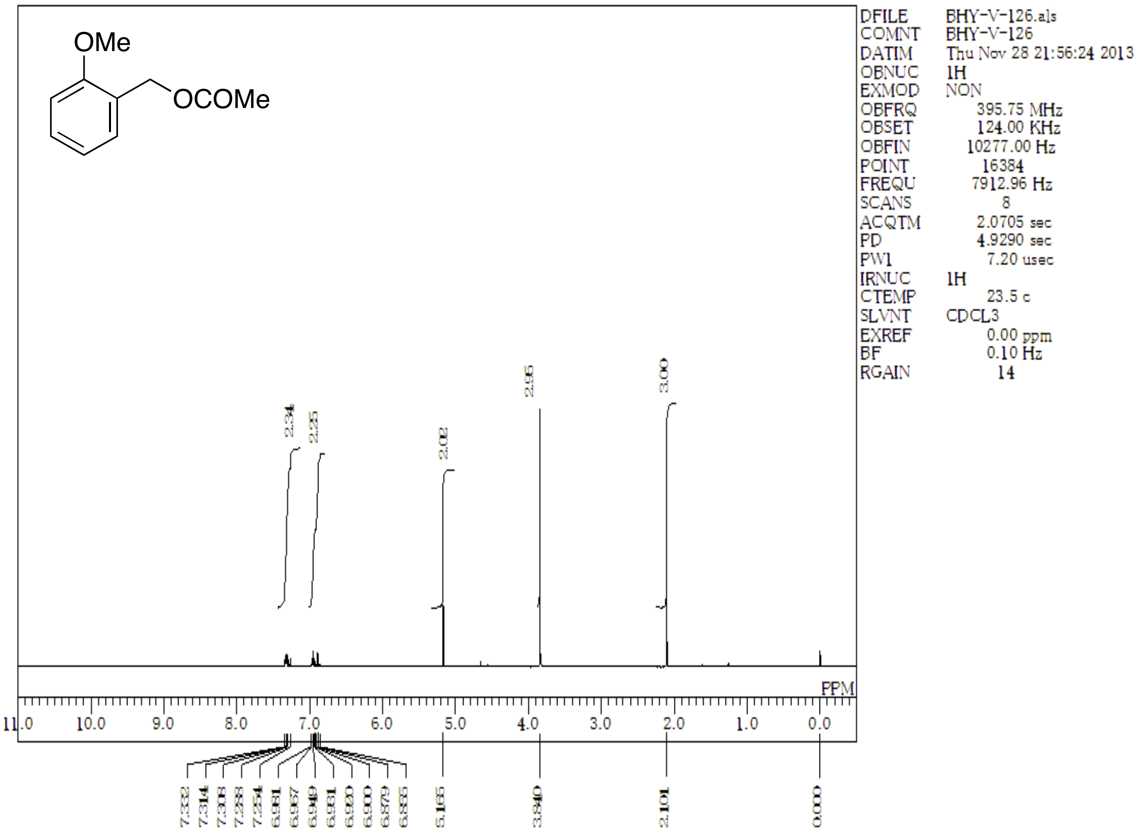
OMe
OCOMe
DFILE BHY-V-126.als
COMNT BHY-V-126
DATIM Thu Nov 28 21:56:24 2013
OBNUC 1H
EXMOD NON
OBFRQ 395.75 MHz
OBSET 124.00 KHz
OBFIN 10277.00 Hz
POINT 16384
FREQU 7912.96 Hz
SCANS 8
ACQTM 2.0705 sec
PD 4.9290 sec
PW1 7.20 usec
IRNUC 1H
CTEMP 23.5 c
SLVNT CDCL3
EXREF 0.00 ppm
BF 0.10 Hz
RGAIN 14
PPM

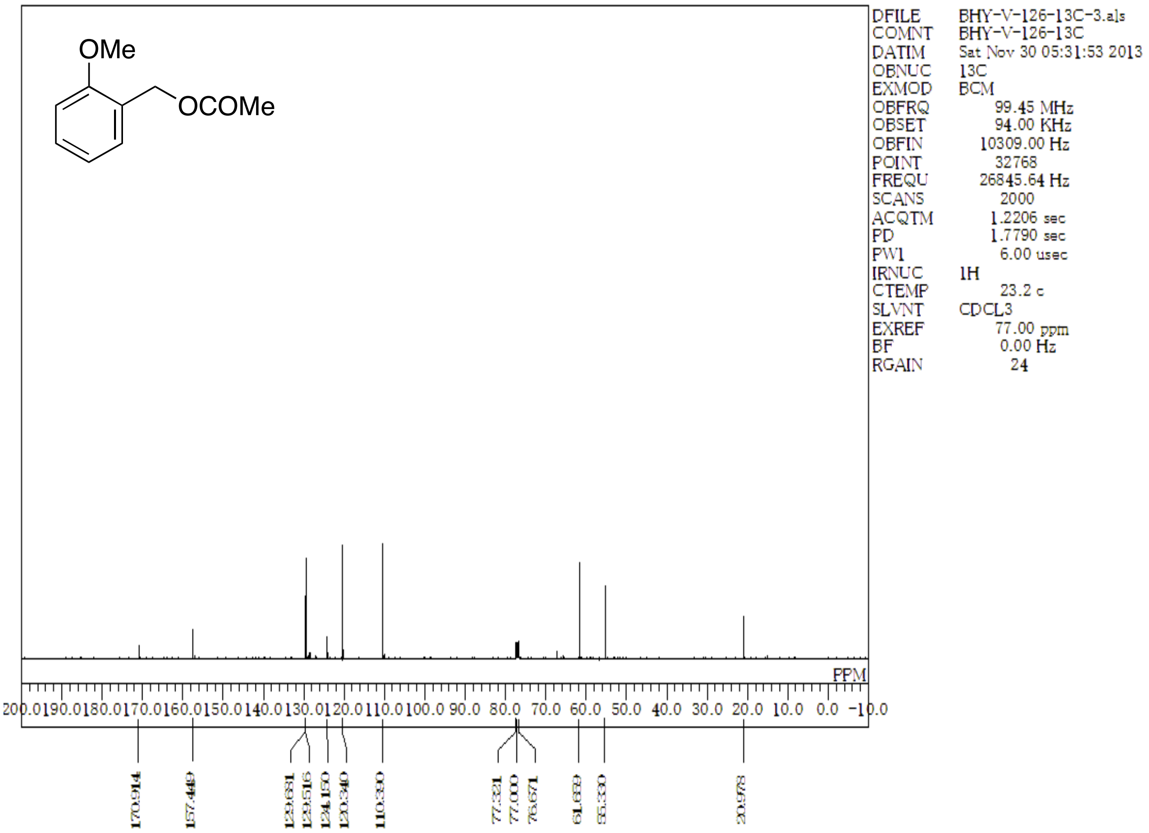
OMe
OCOMe
DFILE BHY-V-126-13C-3.als
COMNT BHY-V-126-13C
DATIM Sat Nov 30 05:31:53 2013
OBNUC 13C
EXMOD BCM
OBFRQ 99.45 MHz
OBSET 94.00 KHz
OBFIN 10309.00 Hz
POINT 32768
FREQU 26845.64 Hz
SCANS 2000
ACQTM 1.2206 sec
PD 1.7790 sec
PW1 6.00 usec
IRNUC 1H
CTEMP 23.2 c
SLVNT CDCL3
EXREF 77.00 ppm
BF 0.00 Hz
RGAIN 24
PPM

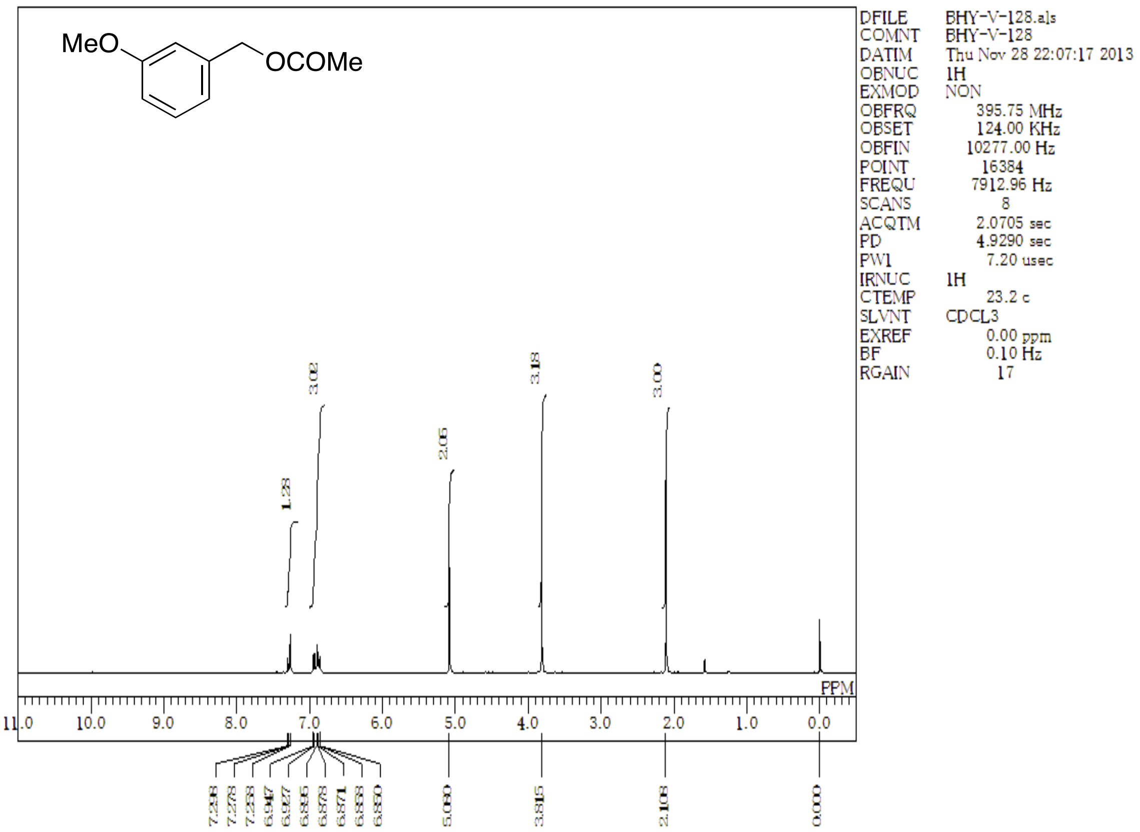
MeO
OCOMe
DFILE BHY-V-128.als
COMNT BHY-V-128
DATIM Thu Nov 28 22:07:17 2013
OBNUC 1H
EXMOD NON
OBFRQ 395.75 MHz
OBSET 124.00 KHz
OBFIN 10277.00 Hz
POINT 16384
FREQU 7912.96 Hz
SCANS 8
ACQTM 2.0705 sec
PD 4.9290 sec
PW1 7.20 usec
IRNUC 1H
CTEMP 23.2 c
SLVNT CDCL3
EXREF 0.00 ppm
BF 0.10 Hz
RGAIN 17
PPM
5.080
3.815
2.108
0.000

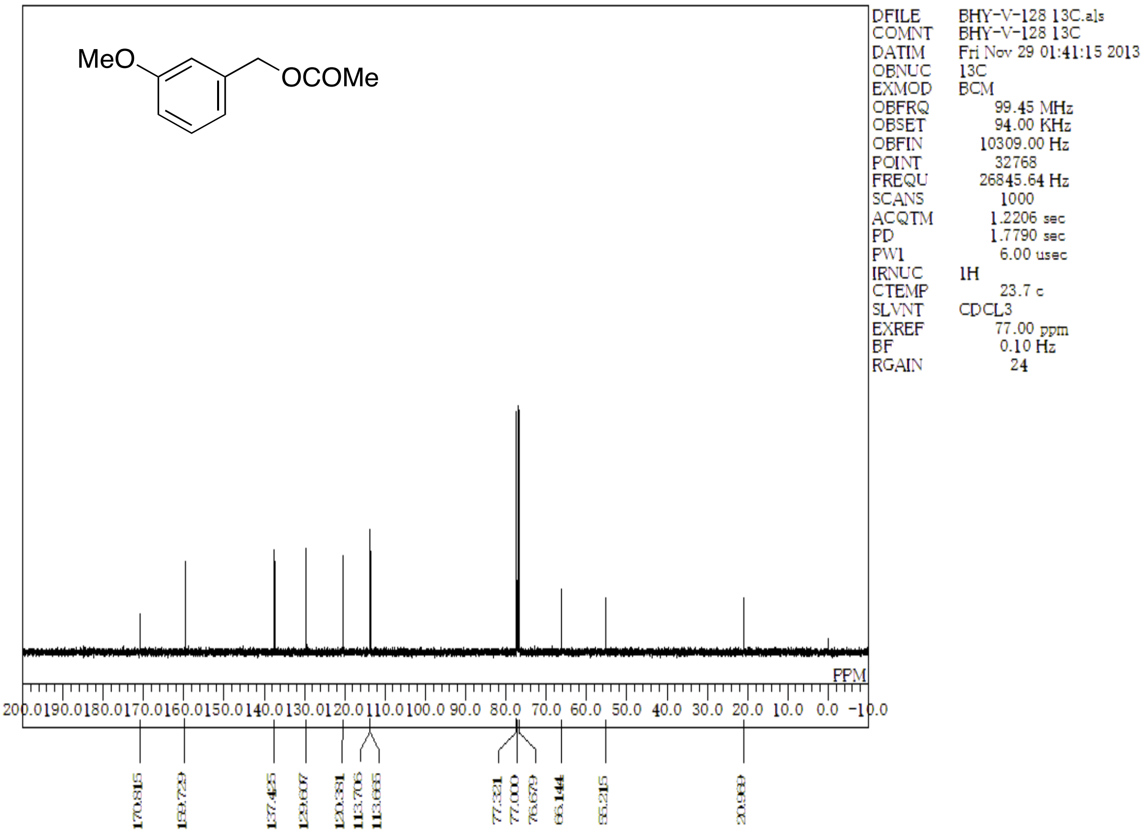
MeO
OCOMe
DFILE BHY-V-128 13C.als
COMNT BHY-V-128 13C
DATIM Fri Nov 29 01:41:15 2013
OBNUC 13C
EXMOD BCM
OBFRQ 99.45 MHz
OBSET 94.00 KHz
OBFIN 10309.00 Hz
POINT 32768
FREQU 26845.64 Hz
SCANS 1000
ACQTM 1.2206 sec
PD 1.7790 sec
PW1 6.00 usec
IRNUC 1H
CTEMP 23.7 c
SLVNT CDCL3
EXREF 77.00 ppm
BF 0.10 Hz
RGAIN 24
PPM
170.815
159.729
137.425
129.607
120.381
113.706
113.685
77.321
77.000
76.679
66.144
55.215
20.989

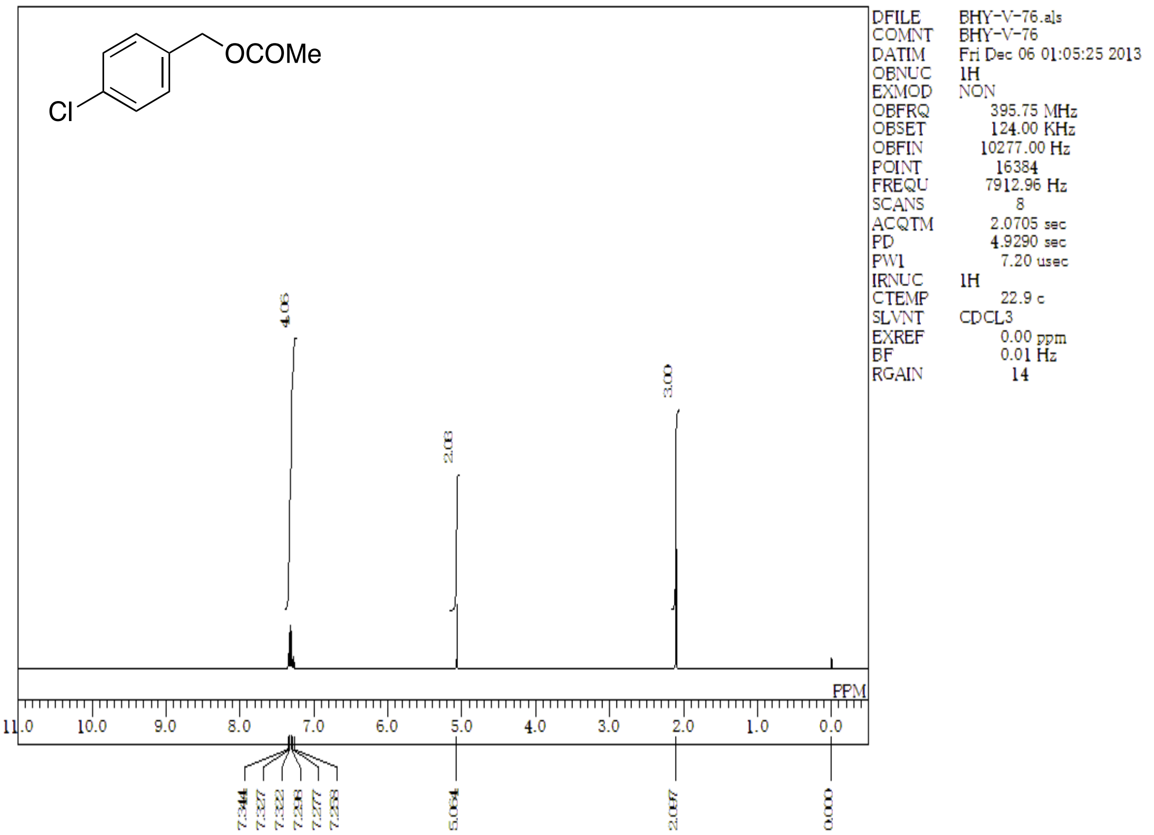
OCOMe
Cl
DFILE BHY-V-76.als
COMNT BHY-V-76
DATIM Fri Dec 06 01:05:25 2013
OBNUC 1H
EXMOD NON
OBFRQ 395.75 MHz
OBSET 124.00 KHz
OBFIN 10277.00 Hz
POINT 16384
FREQU 7912.96 Hz
SCANS 8
ACQTM 2.0705 sec
PD 4.9290 sec
PW1 7.20 usec
IRNUC 1H
CTEMP 22.9 c
SLVNT CDCL3
EXREF 0.00 ppm
BF 0.01 Hz
RGAIN 14
PPM
7.344
7.327
7.322
7.298
7.277
7.258
5.064
2.087
0.000

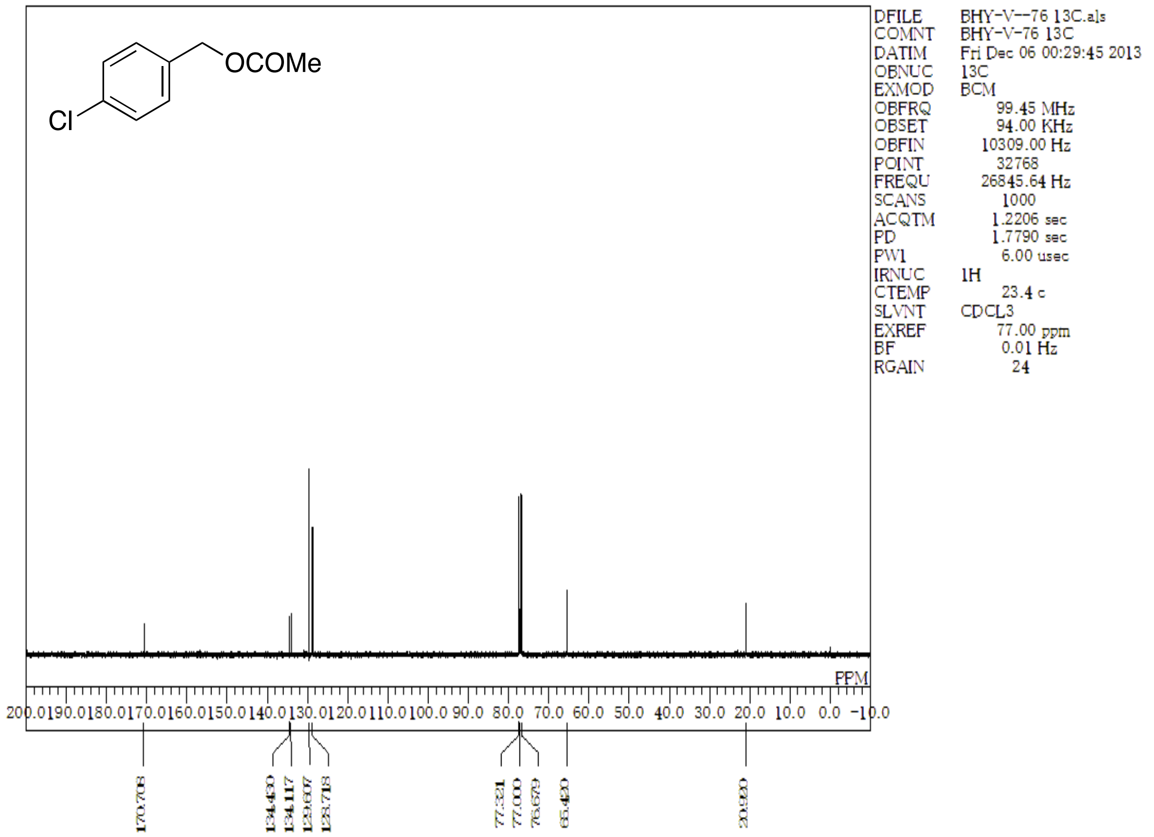
OCOMe
Cl
DFILE BHY-V--76 13C.als
COMNT BHY-V-76 13C
DATIM Fri Dec 06 00:29:45 2013
OBNUC 13C
EXMOD BCM
OBFRQ 99.45 MHz
OBSET 94.00 KHz
OBFIN 10309.00 Hz
POINT 32768
FREQU 26845.64 Hz
SCANS 1000
ACQTM 1.2206 sec
PD 1.7790 sec
PW1 6.00 usec
IRNUC 1H
CTEMP 23.4 c
SLVNT CDCL3
EXREF 77.00 ppm
BF 0.01 Hz
RGAIN 24
PPM
170.708
134.430
134.117
129.607
128.718
77.321
77.000
76.679
65.420
20.920

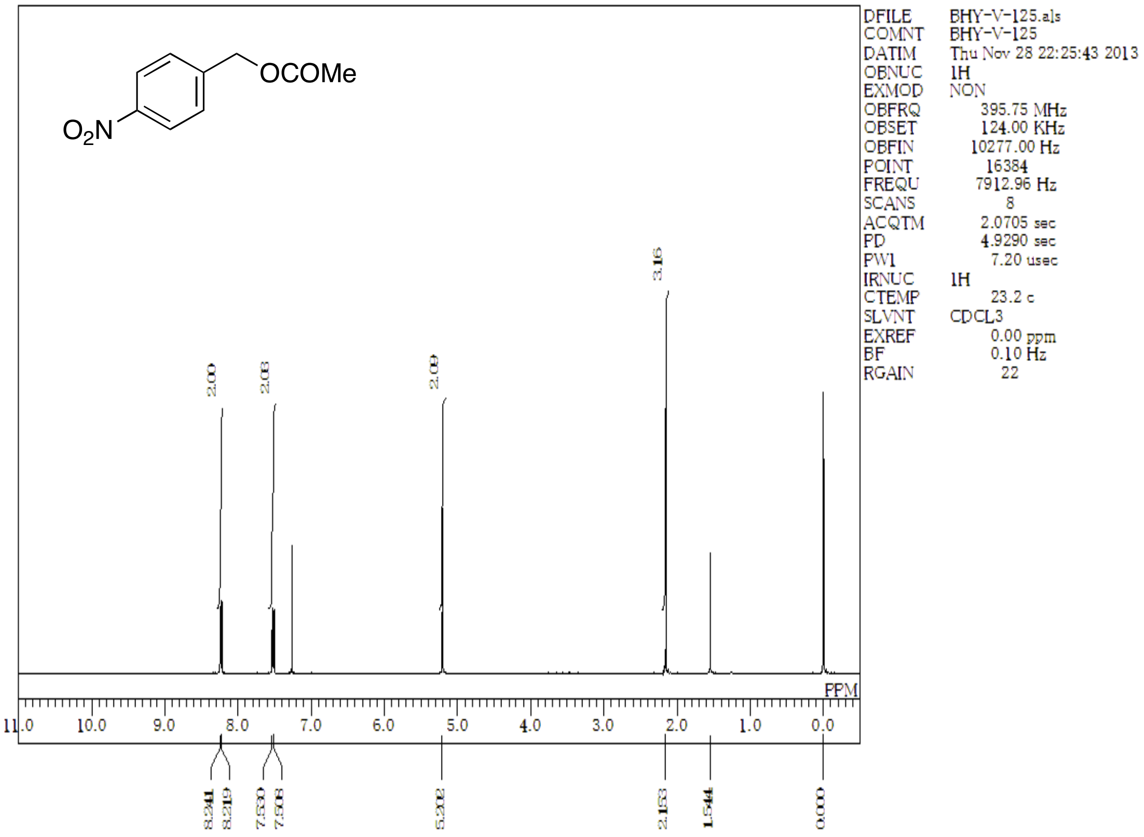
OCOMe
O2N
DFILE BHY-V-125.als
COMNT BHY-V-125
DATIM Thu Nov 28 22:25:43 2013
OBNUC 1H
EXMOD NON
OBFRQ 395.75 MHz
OBSET 124.00 KHz
OBFIN 10277.00 Hz
POINT 16384
FREQU 7912.96 Hz
SCANS 8
ACQTM 2.0705 sec
PD 4.9290 sec
PW1 7.20 usec
IRNUC 1H
CTEMP 23.2 c
SLVNT CDCL3
EXREF 0.00 ppm
BF 0.10 Hz
RGAIN 22
PPM

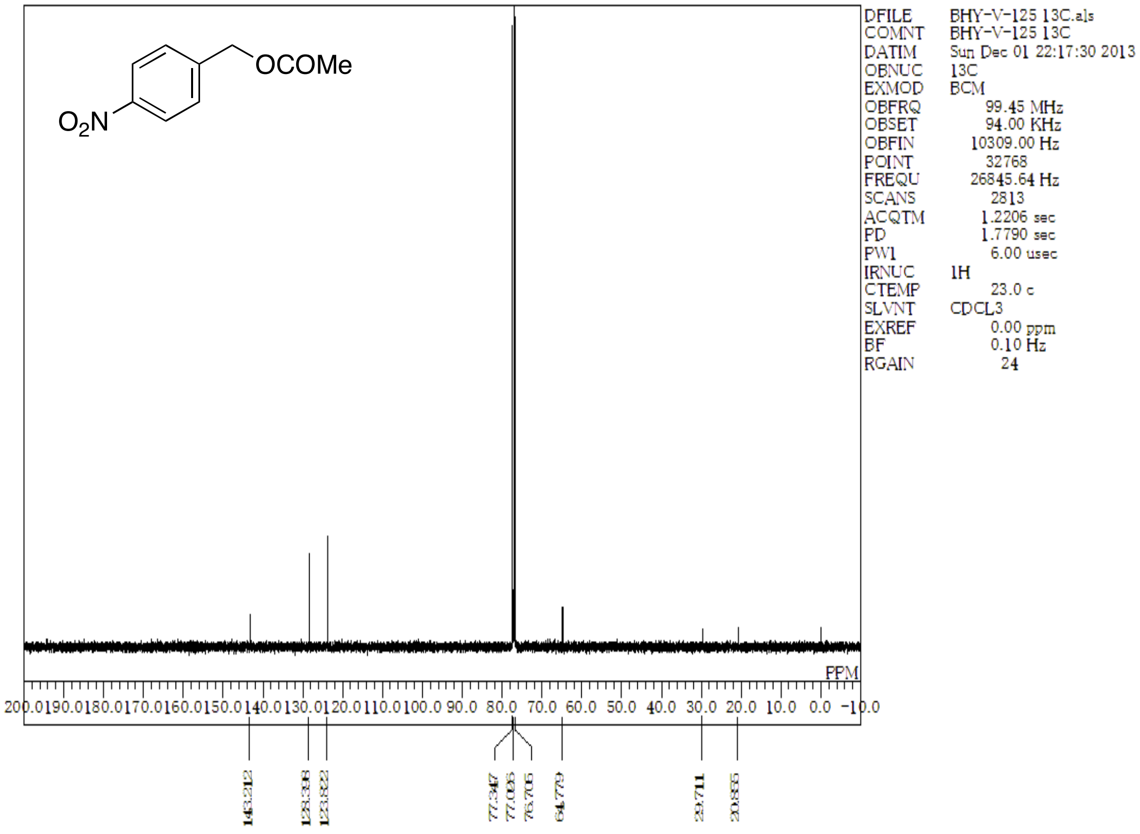
OCOMe
O2N
DFILE BHY-V-125 13C.als
COMNT BHY-V-125 13C
DATIM Sun Dec 01 22:17:30 2013
OBNUC 13C
EXMOD BCM
OBFRQ 99.45 MHz
OBSET 94.00 KHz
OBFIN 10309.00 Hz
POINT 32768
FREQU 26845.64 Hz
SCANS 2813
ACQTM 1.2206 sec
PD 1.7790 sec
PW1 6.00 usec
IRNUC 1H
CTEMP 23.0 c
SLVNT CDCL3
EXREF 0.00 ppm
BF 0.10 Hz
RGAIN 24
PPM

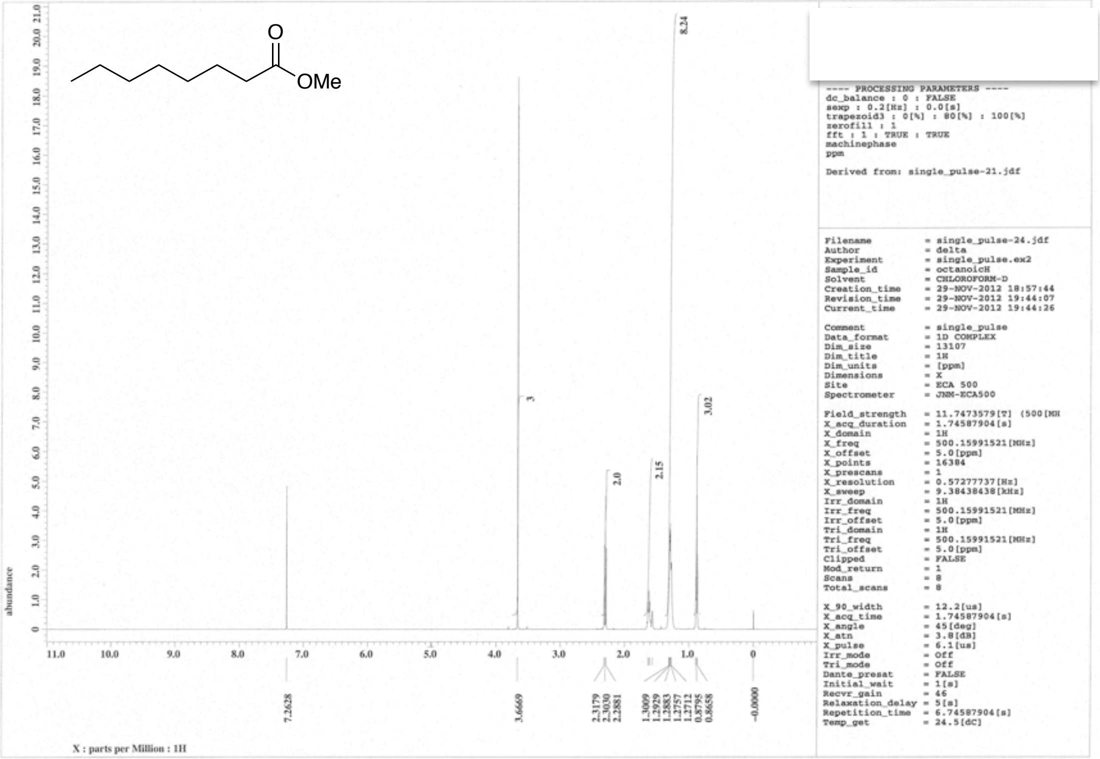
OMe
O
---- PROCESSING PARAMETERS ----
dc_balance : 0 : FALSE
sexp : 0.2[Hz] : 0.0[s]
trapezoid3 : 0[%] : 80[%] : 100[%]
zerofill : 1
fft : 1 : TRUE : TRUE
machinephase
ppm
Derived from: single_pulse-21.jdf
Filename = single_pulse-24.jdf
Author = delta
Experiment = single_pulse.ex2
Sample_id = octanoicH
Solvent = CHLOROFORM-D
Creation_time = 29-NOV-2012 18:57:44
Revision_time = 29-NOV-2012 19:44:07
Current_time = 29-NOV-2012 19:44:26
Comment = single_pulse
Data_format = 1D COMPLEX
Dim_size = 13107
Dim_title = 1H
Dim_units = [ppm]
Dimensions = X
Site = ECA 500
Spectrometer = JNM-ECA500
Field_strength = 11.7473579[T] (500[MH
X_acq_duration = 1.74587904[s]
X_domain = 1H
X_freq = 500.15991521[MHz]
X_offset = 5.0[ppm]
X_points = 16384
X_prescans = 1
X_resolution = 0.57277737[Hz]
X_sweep = 9.38438438[kHz]
Irr_domain = 1H
Irr_freq = 500.15991521[MHz]
Irr_offset = 5.0[ppm]
Tri_domain = 1H
Tri_freq = 500.15991521[MHz]
Tri_offset = 5.0[ppm]
Clipped = FALSE
Mod_return = 1
Scans = 8
Total_scans = 8
X_90_width = 12.2[us]
X_acq_time = 1.74587904[s]
X_angle = 45[deg]
X_atn = 3.8[dB]
X_pulse = 6.1[us]
Irr_mode = Off
Tri_mode = Off
Dante_presat = FALSE
Initial_wait = 1[s]
Recvr_gain = 46
Relaxation_delay = 5[s]
Repetition_time = 6.74587904[s]
Temp_get = 24.5[dC]
8.24
3
3.02
2.0
2.15
7.2628
3.6669
2.3179
2.3030
2.2881
1.3009
1.2929
1.2883
1.2757
1.2712
0.8795
0.8658
-0.0000
abundance
X : parts per Million : 1H

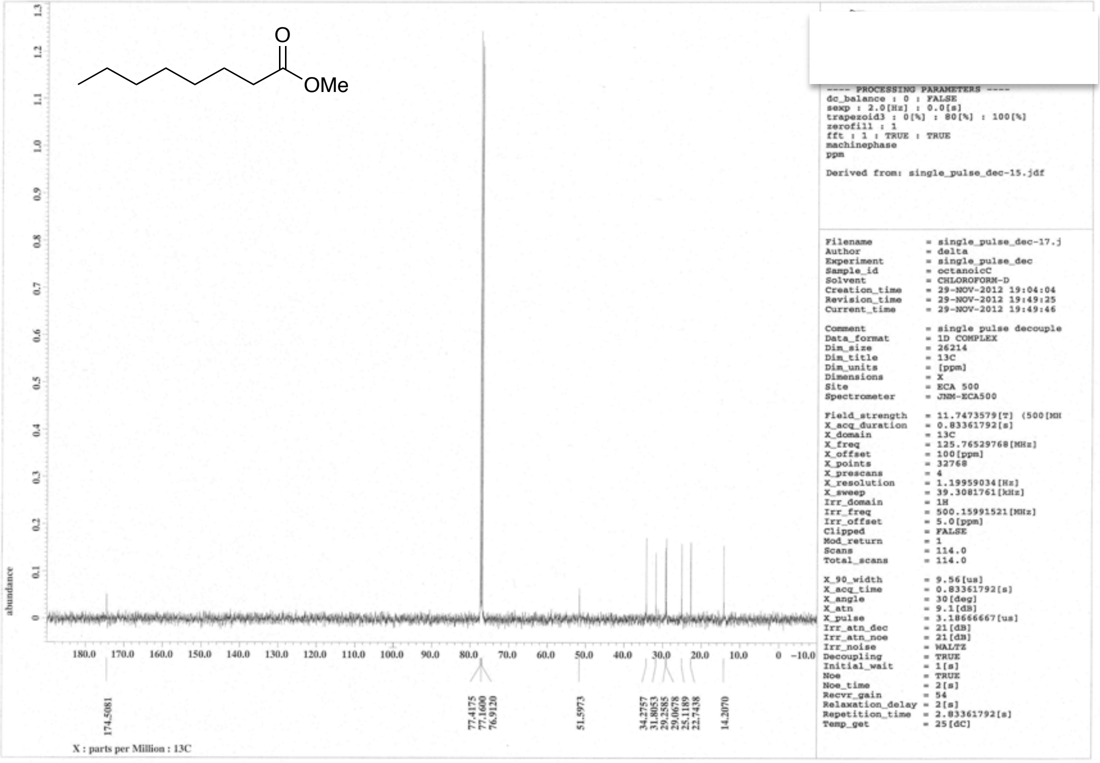
OMe
O
---- PROCESSING PARAMETERS ----
dc_balance : 0 : FALSE
sexp : 2.0[Hz] : 0.0[s]
trapezoid3 : 0[%] : 80[%] : 100[%]
zerofill : 1
fft : 1 : TRUE : TRUE
machinephase
ppm
Derived from: single_pulse_dec-15.jdf
Filename = single_pulse_dec-17.j
Author = delta
Experiment = single_pulse_dec
Sample_id = octanoicC
Solvent = CHLOROFORM-D
Creation_time = 29-NOV-2012 19:04:04
Revision_time = 29-NOV-2012 19:49:25
Current_time = 29-NOV-2012 19:49:46
Comment = single pulse decouple
Data_format = 1D COMPLEX
Dim_size = 26214
Dim_title = 13C
Dim_units = [ppm]
Dimensions = X
Site = ECA 500
Spectrometer = JNM-ECA500
Field_strength = 11.7473579[T] (500[MH
X_acq_duration = 0.83361792[s]
X_domain = 13C
X_freq = 125.76529768[MHz]
X_offset = 100[ppm]
X_points = 32768
X_prescans = 4
X_resolution = 1.19959034[Hz]
X_sweep = 39.3081761[kHz]
Irr_domain = 1H
Irr_freq = 500.15991521[MHz]
Irr_offset = 5.0[ppm]
Clipped = FALSE
Mod_return = 1
Scans = 114.0
Total_scans = 114.0
X_90_width = 9.56[us]
X_acq_time = 0.83361792[s]
X_angle = 30[deg]
X_atn = 9.1[dB]
X_pulse = 3.18666667[us]
Irr_atn_dec = 21[dB]
Irr_atn_noe = 21[dB]
Irr_noise = WALTZ
Decoupling = TRUE
Initial_wait = 1[s]
Noe = TRUE
Noe_time = 2[s]
Recvr_gain = 54
Relaxation_delay = 2[s]
Repetition_time = 2.83361792[s]
Temp_get = 25[dC]
174.5081
77.4175
77.1600
76.9120
51.5973
34.2757
31.8053
29.2585
29.0678
25.1189
22.7438
14.2070
abundance
X : parts per Million : 13C

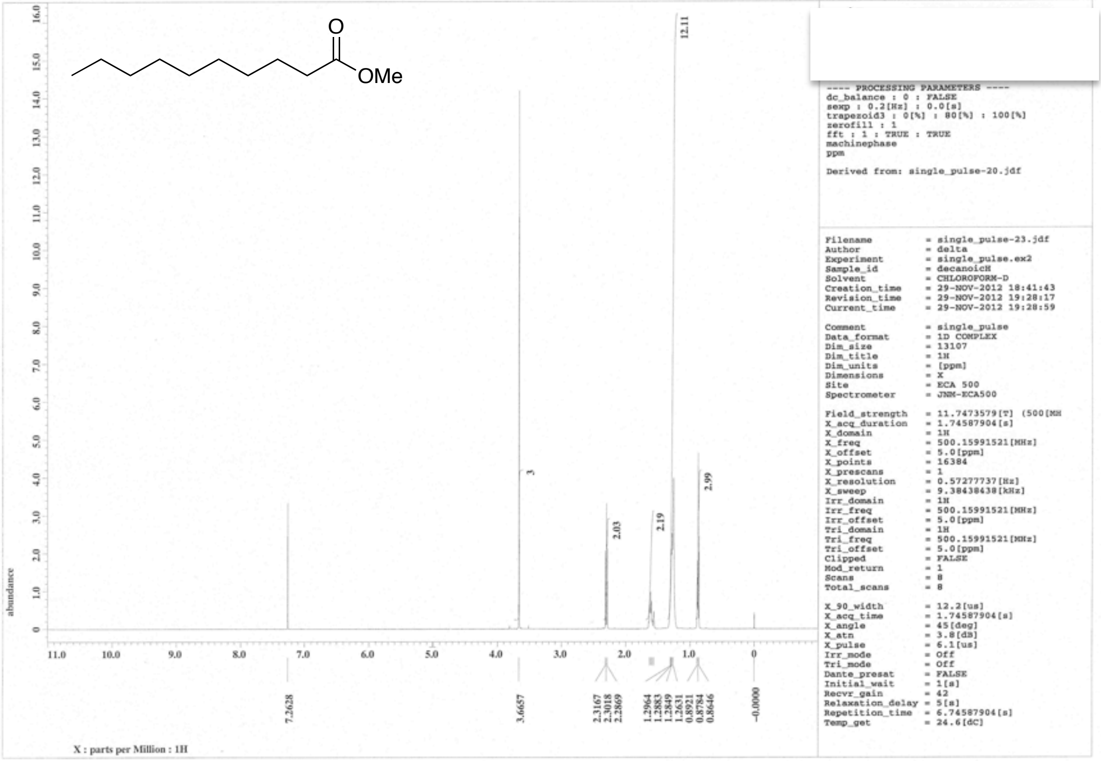
O
OMe
12.11
3
2.03
2.19
2.99
abundance
X : parts per Million : 1H
7.2628
3.6657
2.3167
2.3018
2.2869
1.2964
1.2883
1.2849
1.2631
0.8921
0.8784
0.8646
-0.0000
---- PROCESSING PARAMETERS ----
dc_balance : 0 : FALSE
sexp : 0.2[Hz] : 0.0[s]
trapezoid3 : 0[%] : 80[%] : 100[%]
zerofill : 1
fft : 1 : TRUE : TRUE
machinephase
ppm
Derived from: single_pulse-20.jdf
Filename = single_pulse-23.jdf
Author = delta
Experiment = single_pulse.ex2
Sample_id = decanoicH
Solvent = CHLOROFORM-D
Creation_time = 29-NOV-2012 18:41:43
Revision_time = 29-NOV-2012 19:28:17
Current_time = 29-NOV-2012 19:28:59
Comment = single_pulse
Data_format = 1D COMPLEX
Dim_size = 13107
Dim_title = 1H
Dim_units = [ppm]
Dimensions = X
Site = ECA 500
Spectrometer = JNM-ECA500
Field_strength = 11.7473579[T] (500[MH
X_acq_duration = 1.74587904[s]
X_domain = 1H
X_freq = 500.15991521[MHz]
X_offset = 5.0[ppm]
X_points = 16384
X_prescans = 1
X_resolution = 0.57277737[Hz]
X_sweep = 9.38438438[kHz]
Irr_domain = 1H
Irr_freq = 500.15991521[MHz]
Irr_offset = 5.0[ppm]
Tri_domain = 1H
Tri_freq = 500.15991521[MHz]
Tri_offset = 5.0[ppm]
Clipped = FALSE
Mod_return = 1
Scans = 8
Total_scans = 8
X_90_width = 12.2[us]
X_acq_time = 1.74587904[s]
X_angle = 45[deg]
X_atn = 3.8[dB]
X_pulse = 6.1[us]
Irr_mode = Off
Tri_mode = Off
Dante_presat = FALSE
Initial_wait = 1[s]
Recvr_gain = 42
Relaxation_delay = 5[s]
Repetition_time = 6.74587904[s]
Temp_get = 24.6[dC]

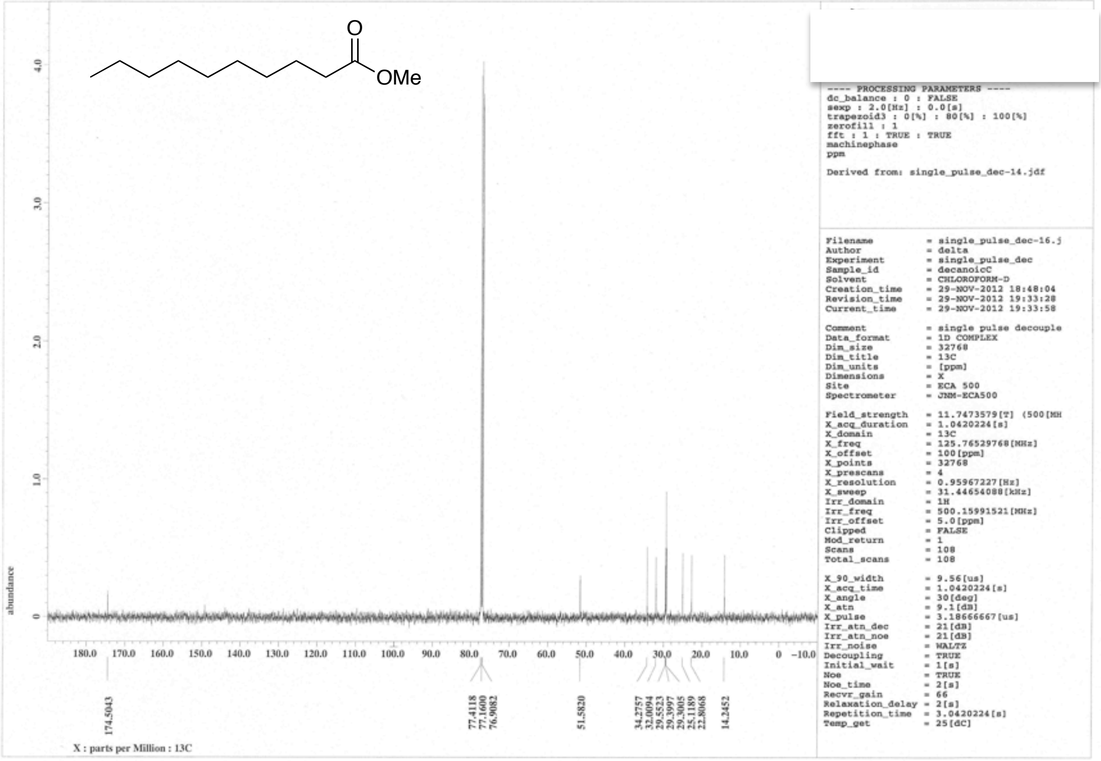
O
OMe
abundance
X : parts per Million : 13C
174.5043
77.4118
77.1600
76.9082
51.5820
34.2757
32.0094
29.5523
29.3997
29.3005
25.1189
22.8068
14.2452
---- PROCESSING PARAMETERS ----
dc_balance : 0 : FALSE
sexp : 2.0[Hz] : 0.0[s]
trapezoid3 : 0[%] : 80[%] : 100[%]
zerofill : 1
fft : 1 : TRUE : TRUE
machinephase
ppm
Derived from: single_pulse_dec-14.jdf
Filename = single_pulse_dec-16.j
Author = delta
Experiment = single_pulse_dec
Sample_id = decanoicC
Solvent = CHLOROFORM-D
Creation_time = 29-NOV-2012 18:48:04
Revision_time = 29-NOV-2012 19:33:28
Current_time = 29-NOV-2012 19:33:58
Comment = single pulse decouple
Data_format = 1D COMPLEX
Dim_size = 32768
Dim_title = 13C
Dim_units = [ppm]
Dimensions = X
Site = ECA 500
Spectrometer = JNM-ECA500
Field_strength = 11.7473579[T] (500[MH
X_acq_duration = 1.0420224[s]
X_domain = 13C
X_freq = 125.76529768[MHz]
X_offset = 100[ppm]
X_points = 32768
X_prescans = 4
X_resolution = 0.95967227[Hz]
X_sweep = 31.44654088[kHz]
Irr_domain = 1H
Irr_freq = 500.15991521[MHz]
Irr_offset = 5.0[ppm]
Clipped = FALSE
Mod_return = 1
Scans = 108
Total_scans = 108
X_90_width = 9.56[us]
X_acq_time = 1.0420224[s]
X_angle = 30[deg]
X_atn = 9.1[dB]
X_pulse = 3.18666667[us]
Irr_atn_dec = 21[dB]
Irr_atn_noe = 21[dB]
Irr_noise = WALTZ
Decoupling = TRUE
Initial_wait = 1[s]
Noe = TRUE
Noe_time = 2[s]
Recvr_gain = 66
Relaxation_delay = 2[s]
Repetition_time = 3.0420224[s]
Temp_get = 25[dC]

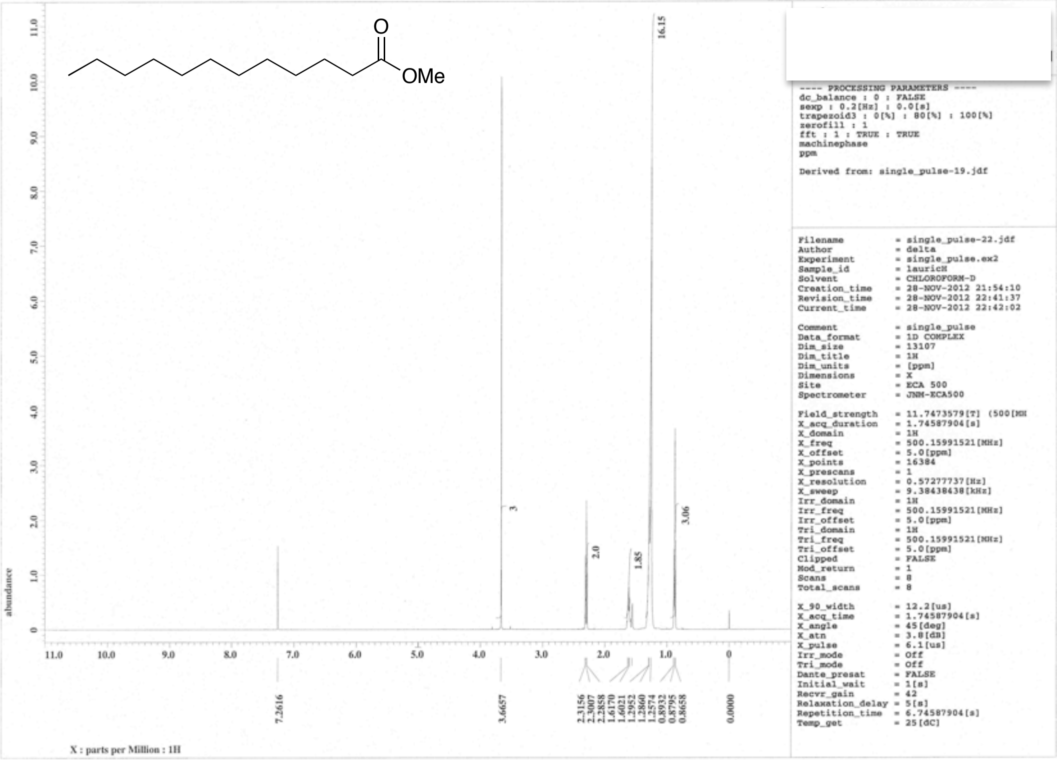

OMe
---- PROCESSING PARAMETERS ----
dc_balance : 0 : FALSE
sexp : 0.2[Hz] : 0.0[s]
trapezoid3 : 0[%] : 80[%] : 100[%]
zerofill : 1
fft : 1 : TRUE : TRUE
machinephase
ppm
Derived from: single_pulse-19.jdf
Filename = single_pulse-22.jdf
Author = delta
Experiment = single_pulse.ex2
Sample_id = lauricH
Solvent = CHLOROFORM-D
Creation_time = 28-NOV-2012 21:54:10
Revision_time = 28-NOV-2012 22:41:37
Current_time = 28-NOV-2012 22:42:02
Comment = single_pulse
Data_format = 1D COMPLEX
Dim_size = 13107
Dim_title = 1H
Dim_units = [ppm]
Dimensions = X
Site = ECA 500
Spectrometer = JNM-ECA500
Field_strength = 11.7473579[T] (500[MH
X_acq_duration = 1.74587904[s]
X_domain = 1H
X_freq = 500.15991521[MHz]
X_offset = 5.0[ppm]
X_points = 16384
X_prescans = 1
X_resolution = 0.57277737[Hz]
X_sweep = 9.38438438[kHz]
Irr_domain = 1H
Irr_freq = 500.15991521[MHz]
Irr_offset = 5.0[ppm]
Tri_domain = 1H
Tri_freq = 500.15991521[MHz]
Tri_offset = 5.0[ppm]
Clipped = FALSE
Mod_return = 1
Scans = 8
Total_scans = 8
X_90_width = 12.2[us]
X_acq_time = 1.74587904[s]
X_angle = 45[deg]
X_atn = 3.8[dB]
X_pulse = 6.1[us]
Irr_mode = Off
Tri_mode = Off
Dante_presat = FALSE
Initial_wait = 1[s]
Recvr_gain = 42
Relaxation_delay = 5[s]
Repetition_time = 6.74587904[s]
Temp_get = 25[dC]
16.15
3
2.0
1.85
3.06
7.2616
3.6657
2.3156
2.3007
2.2858
1.6170
1.6021
1.2952
1.2860
1.2574
0.8932
0.8795
0.8658
0.0000
abundance
X : parts per Million : 1H

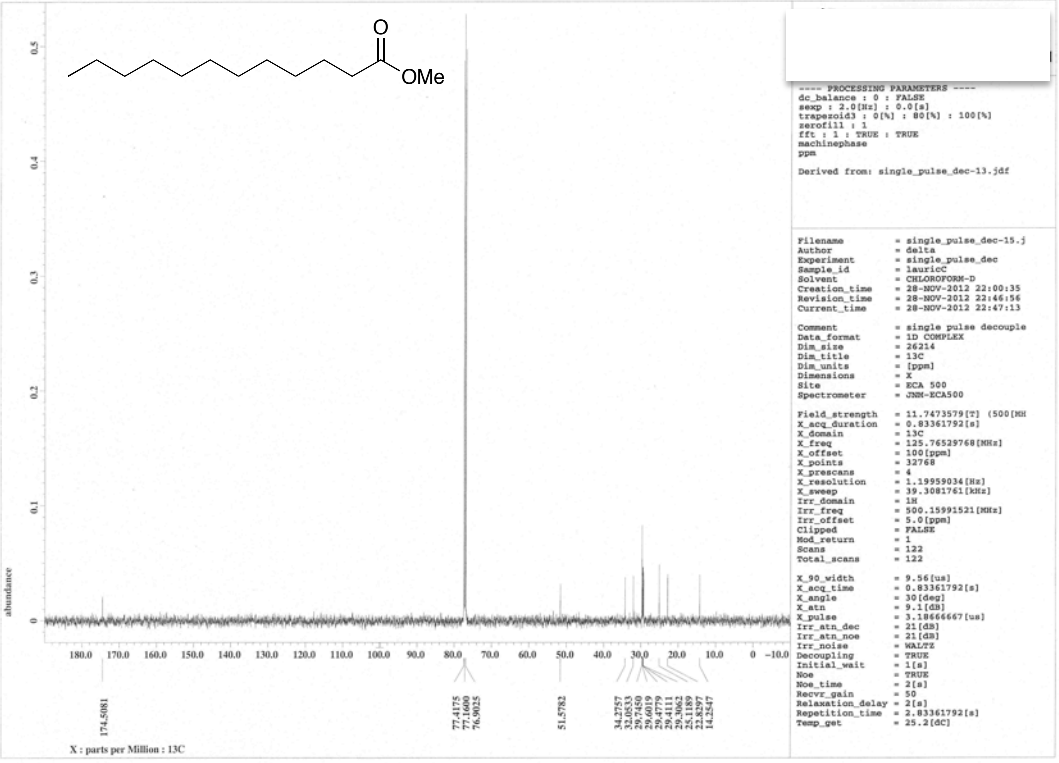

OMe
---- PROCESSING PARAMETERS ----
dc_balance : 0 : FALSE
sexp : 2.0[Hz] : 0.0[s]
trapezoid3 : 0[%] : 80[%] : 100[%]
zerofill : 1
fft : 1 : TRUE : TRUE
machinephase
ppm
Derived from: single_pulse_dec-13.jdf
Filename = single_pulse_dec-15.j
Author = delta
Experiment = single_pulse_dec
Sample_id = lauricC
Solvent = CHLOROFORM-D
Creation_time = 28-NOV-2012 22:00:35
Revision_time = 28-NOV-2012 22:46:56
Current_time = 28-NOV-2012 22:47:13
Comment = single pulse decouple
Data_format = 1D COMPLEX
Dim_size = 26214
Dim_title = 13C
Dim_units = [ppm]
Dimensions = X
Site = ECA 500
Spectrometer = JNM-ECA500
Field_strength = 11.7473579[T] (500[MH
X_acq_duration = 0.83361792[s]
X_domain = 13C
X_freq = 125.76529768[MHz]
X_offset = 100[ppm]
X_points = 32768
X_prescans = 4
X_resolution = 1.19959034[Hz]
X_sweep = 39.3081761[kHz]
Irr_domain = 1H
Irr_freq = 500.15991521[MHz]
Irr_offset = 5.0[ppm]
Clipped = FALSE
Mod_return = 1
Scans = 122
Total_scans = 122
X_90_width = 9.56[us]
X_acq_time = 0.83361792[s]
X_angle = 30[deg]
X_atn = 9.1[dB]
X_pulse = 3.18666667[us]
Irr_atn_dec = 21[dB]
Irr_atn_noe = 21[dB]
Irr_noise = WALTZ
Decoupling = TRUE
Initial_wait = 1[s]
Noe = TRUE
Noe_time = 2[s]
Recvr_gain = 50
Relaxation_delay = 2[s]
Repetition_time = 2.83361792[s]
Temp_get = 25.2[dC]
174.5081
77.4175
77.1600
76.9025
51.5782
34.2757
32.0533
29.7450
29.6019
29.4779
29.4111
29.3062
25.1189
22.8297
14.2547
abundance
X : parts per Million : 13C

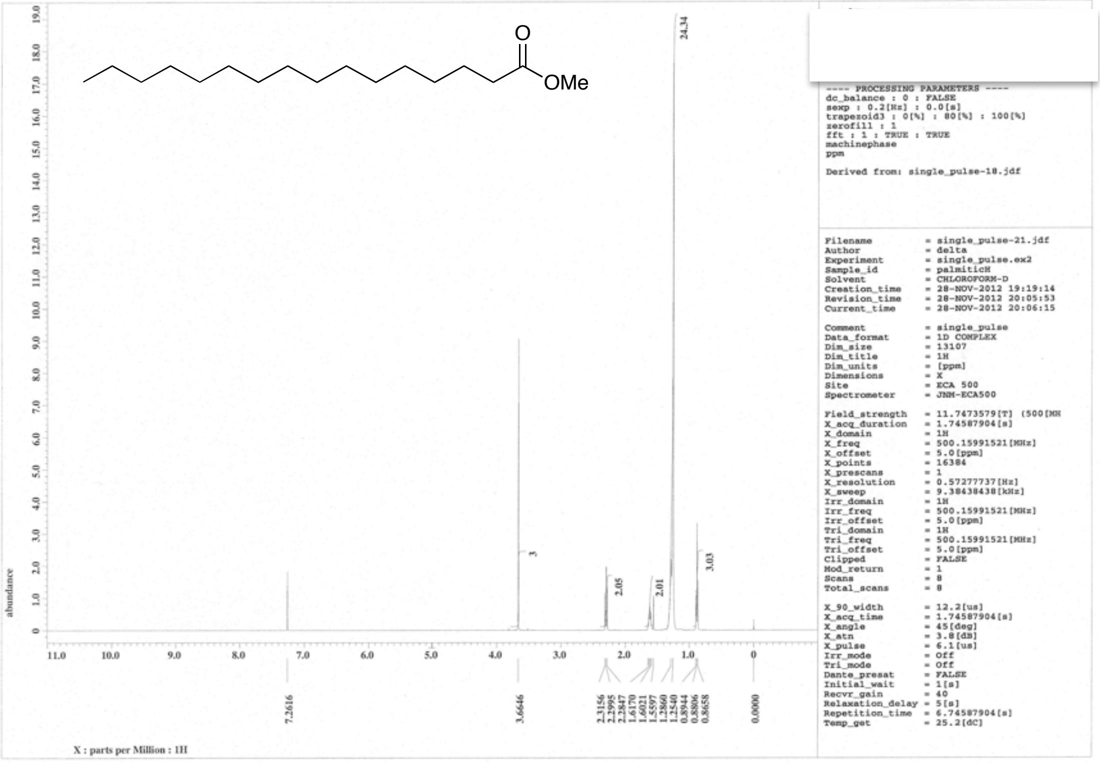
O
OMe
24.34
3
2.05
2.01
3.03
7.2616
3.6646
2.3156
2.2995
2.2847
1.6170
1.6021
1.5597
1.2860
1.2540
0.8944
0.8806
0.8658
0.0000
X : parts per Million : 1H
abundance
---- PROCESSING PARAMETERS ----
dc_balance : 0 : FALSE
sexp : 0.2[Hz] : 0.0[s]
trapezoid3 : 0[%] : 80[%] : 100[%]
zerofill : 1
fft : 1 : TRUE : TRUE
machinephase
ppm
Derived from: single_pulse-18.jdf
Filename = single_pulse-21.jdf
Author = delta
Experiment = single_pulse.ex2
Sample_id = palmiticH
Solvent = CHLOROFORM-D
Creation_time = 28-NOV-2012 19:19:14
Revision_time = 28-NOV-2012 20:05:53
Current_time = 28-NOV-2012 20:06:15
Comment = single_pulse
Data_format = 1D COMPLEX
Dim_size = 13107
Dim_title = 1H
Dim_units = [ppm]
Dimensions = X
Site = ECA 500
Spectrometer = JNM-ECA500
Field_strength = 11.7473579[T] (500[MH
X_acq_duration = 1.74587904[s]
X_domain = 1H
X_freq = 500.15991521[MHz]
X_offset = 5.0[ppm]
X_points = 16384
X_prescans = 1
X_resolution = 0.57277737[Hz]
X_sweep = 9.38438438[kHz]
Irr_domain = 1H
Irr_freq = 500.15991521[MHz]
Irr_offset = 5.0[ppm]
Tri_domain = 1H
Tri_freq = 500.15991521[MHz]
Tri_offset = 5.0[ppm]
Clipped = FALSE
Mod_return = 1
Scans = 8
Total_scans = 8
X_90_width = 12.2[us]
X_acq_time = 1.74587904[s]
X_angle = 45[deg]
X_atn = 3.8[dB]
X_pulse = 6.1[us]
Irr_mode = Off
Tri_mode = Off
Dante_presat = FALSE
Initial_wait = 1[s]
Recvr_gain = 40
Relaxation_delay = 5[s]
Repetition_time = 6.74587904[s]
Temp_get = 25.2[dC]

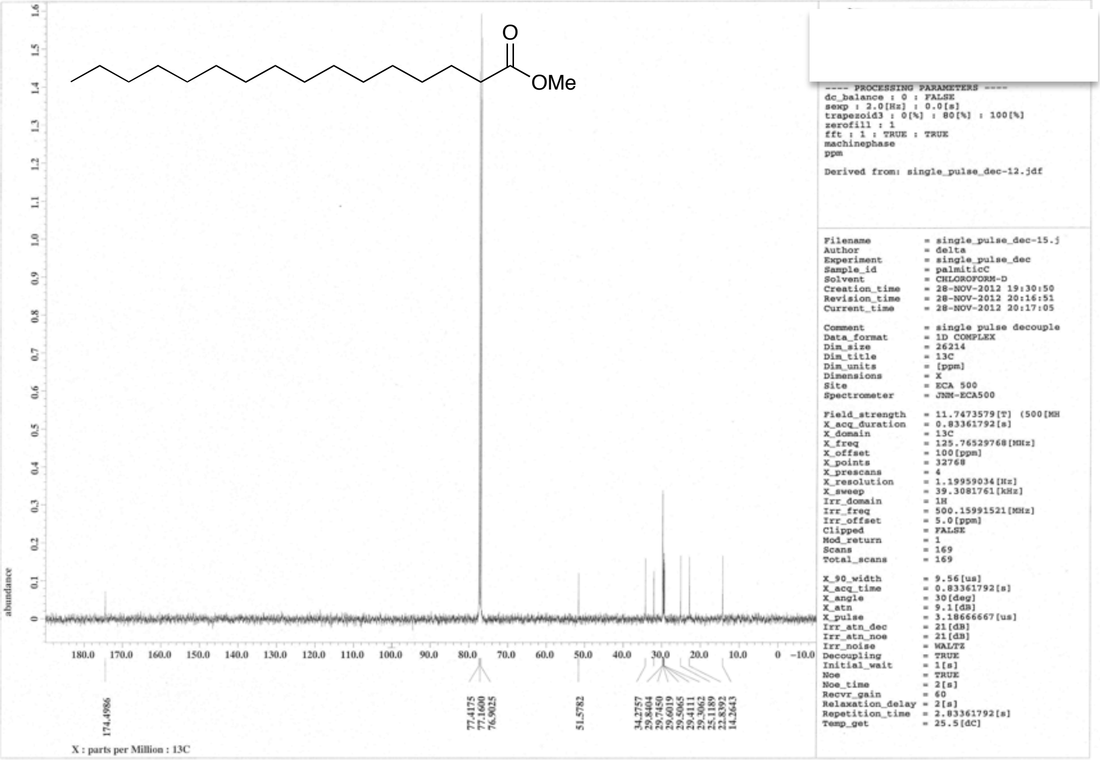
O
OMe
174.4986
77.4175
77.1600
76.9025
51.5782
34.2757
29.8404
29.7450
29.6019
29.5065
29.4111
29.3062
25.1189
22.8392
14.2643
X : parts per Million : 13C
abundance
---- PROCESSING PARAMETERS ----
dc_balance : 0 : FALSE
sexp : 2.0[Hz] : 0.0[s]
trapezoid3 : 0[%] : 80[%] : 100[%]
zerofill : 1
fft : 1 : TRUE : TRUE
machinephase
ppm
Derived from: single_pulse_dec-12.jdf
Filename = single_pulse_dec-15.j
Author = delta
Experiment = single_pulse_dec
Sample_id = palmiticC
Solvent = CHLOROFORM-D
Creation_time = 28-NOV-2012 19:30:50
Revision_time = 28-NOV-2012 20:16:51
Current_time = 28-NOV-2012 20:17:05
Comment = single pulse decouple
Data_format = 1D COMPLEX
Dim_size = 26214
Dim_title = 13C
Dim_units = [ppm]
Dimensions = X
Site = ECA 500
Spectrometer = JNM-ECA500
Field_strength = 11.7473579[T] (500[MH
X_acq_duration = 0.83361792[s]
X_domain = 13C
X_freq = 125.76529768[MHz]
X_offset = 100[ppm]
X_points = 32768
X_prescans = 4
X_resolution = 1.19959034[Hz]
X_sweep = 39.3081761[kHz]
Irr_domain = 1H
Irr_freq = 500.15991521[MHz]
Irr_offset = 5.0[ppm]
Clipped = FALSE
Mod_return = 1
Scans = 169
Total_scans = 169
X_90_width = 9.56[us]
X_acq_time = 0.83361792[s]
X_angle = 30[deg]
X_atn = 9.1[dB]
X_pulse = 3.18666667[us]
Irr_atn_dec = 21[dB]
Irr_atn_noe = 21[dB]
Irr_noise = WALTZ
Decoupling = TRUE
Initial_wait = 1[s]
Noe = TRUE
Noe_time = 2[s]
Recvr_gain = 60
Relaxation_delay = 2[s]
Repetition_time = 2.83361792[s]
Temp_get = 25.5[dC]

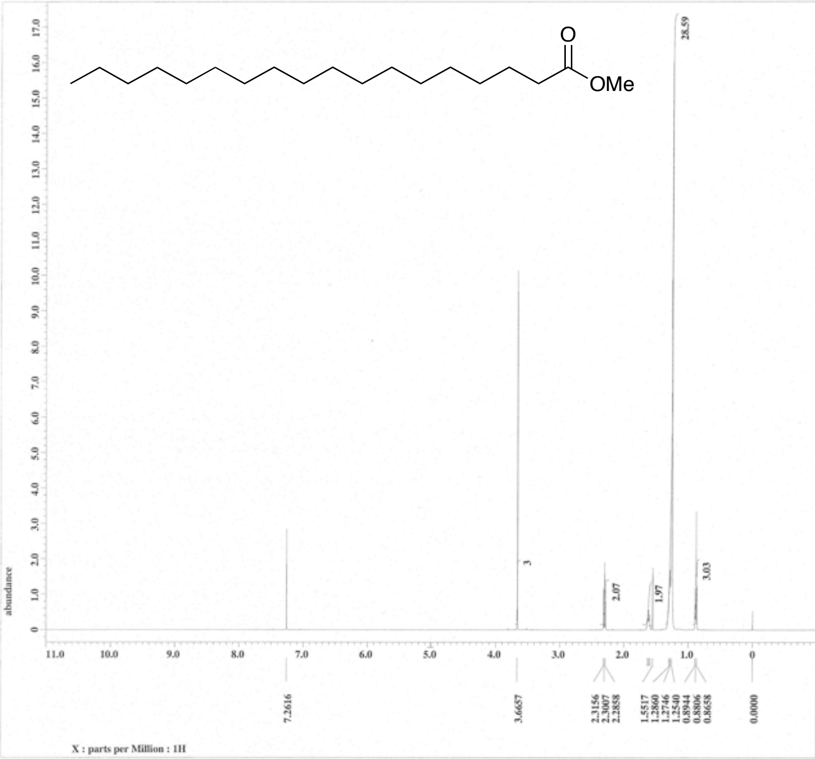


```
---- PROCESSING PARAMETERS ----
dc_balance : 0 : FALSE
sexp : 0.2[Hz] : 0.0[s]
trapezoid3 : 0[%] : 80[%] : 100[%]
zerofill : 1
fft : 1 : TRUE : TRUE
machinephase
ppm

Derived from: single_pulse-17.jdf

Filename          = single_pulse-20.jdf
Author            = delta
Experiment        = single_pulse.ex2
Sample_id         = stearicH
Solvent           = CHLOROFORM-D
Creation_time     = 28-NOV-2012 18:55:51
Revision_time     = 28-NOV-2012 19:43:05
Current_time      = 28-NOV-2012 19:43:52

Comment           = single_pulse
Data_format       = 1D COMPLEX
Dim_size          = 13107
Dim_title         = 1H
Dim_units         = [ppm]
Dimensions        = X
Site              = ECA 500
Spectrometer      = JNM-ECA500

Field_strength    = 11.7473579[T] (500[MH
X_acq_duration    = 1.74587904[s]
X_domain          = 1H
X_freq            = 500.15991521[MHz]
X_offset          = 5.0[ppm]
X_points          = 16384
X_prescans        = 1
X_resolution      = 0.57277737[Hz]
X_sweep           = 9.38438438[kHz]
Irr_domain        = 1H
Irr_freq          = 500.15991521[MHz]
Irr_offset        = 5.0[ppm]
Tri_domain        = 1H
Tri_freq          = 500.15991521[MHz]
Tri_offset        = 5.0[ppm]
Clipped           = FALSE
Mod_return        = 1
Scans             = 8
Total_scans       = 8

X_90_width        = 12.2[us]
X_acq_time        = 1.74587904[s]
X_angle           = 45[deg]
X_atn             = 3.8[dB]
X_pulse           = 6.1[us]
Irr_mode          = Off
Tri_mode          = Off
Dante_presat      = FALSE
Initial_wait      = 1[s]
Recvr_gain        = 42
Relaxation_delay  = 5[s]
Repetition_time   = 6.74587904[s]
Temp_get          = 25.1[dC]
```

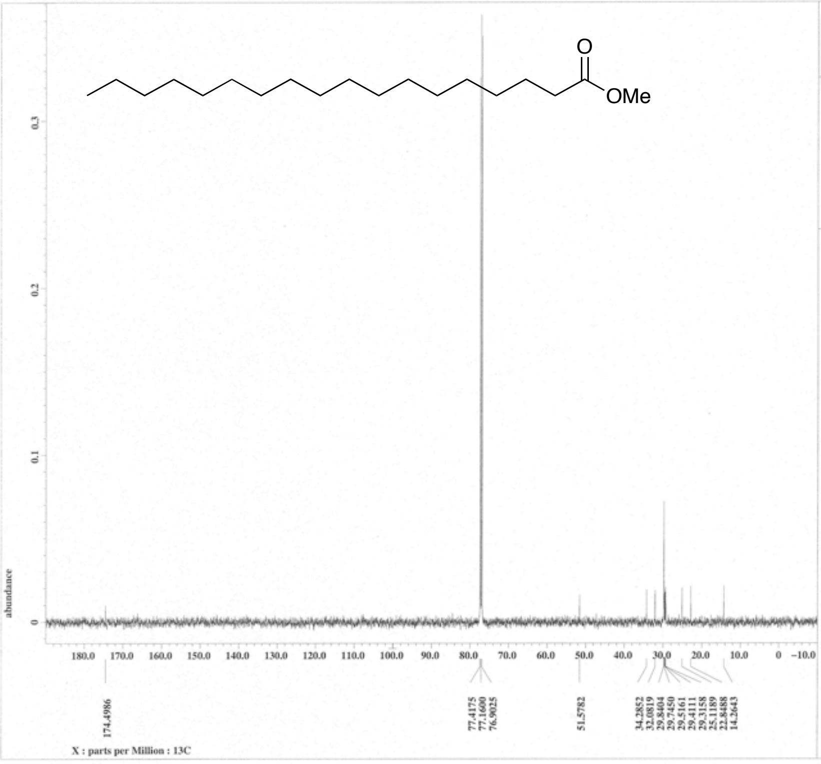


```
---- PROCESSING PARAMETERS ----
dc_balance : 0 : FALSE
sexp : 2.0[Hz] : 0.0[s]
trapezoid3 : 0[%] : 80[%] : 100[%]
zerofill : 1
fft : 1 : TRUE : TRUE
machinephase
ppm

Derived from: single_pulse_dec-11.jdf

Filename          = single_pulse_dec-14.j
Author            = delta
Experiment        = single_pulse_dec
Sample_id         = stearicC
Solvent           = CHLOROFORM-D
Creation_time     = 28-NOV-2012 19:08:46
Revision_time     = 28-NOV-2012 19:54:46
Current_time      = 28-NOV-2012 19:55:18

Comment           = single pulse decouple
Data_format       = 1D COMPLEX
Dim_size          = 26214
Dim_title         = 13C
Dim_units         = [ppm]
Dimensions        = X
Site              = ECA 500
Spectrometer      = JNM-ECA500

Field_strength    = 11.7473579[T] (500[MH
X_acq_duration    = 0.83361792[s]
X_domain          = 13C
X_freq            = 125.76529768[MHz]
X_offset          = 100[ppm]
X_points          = 32768
X_prescans        = 4
X_resolution      = 1.19959034[Hz]
X_sweep           = 39.3081761[kHz]
Irr_domain        = 1H
Irr_freq          = 500.15991521[MHz]
Irr_offset        = 5.0[ppm]
Clipped           = FALSE
Mod_return        = 1
Scans             = 246
Total_scans       = 246

X_90_width        = 9.56[us]
X_acq_time        = 0.83361792[s]
X_angle           = 30[deg]
X_atn             = 9.1[dB]
X_pulse           = 3.18666667[us]
Irr_atn_dec       = 21[dB]
Irr_atn_noe       = 21[dB]
Irr_noise         = WALTZ
Decoupling        = TRUE
Initial_wait      = 1[s]
Noe               = TRUE
Noe_time          = 2[s]
Recvr_gain        = 46
Relaxation_delay  = 2[s]
Repetition_time   = 2.83361792[s]
Temp_get          = 25.3[dC]
```

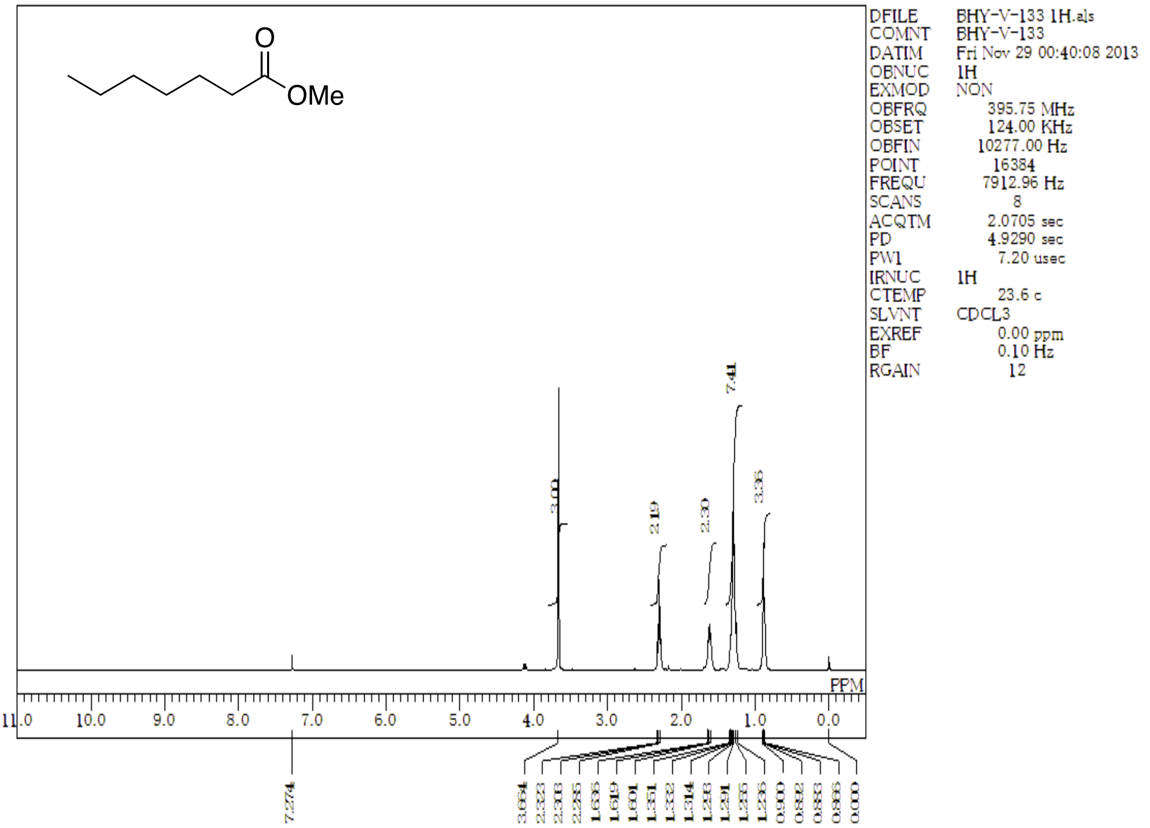
O
OMe
DFILE BHY-V-133 1H.als
COMNT BHY-V-133
DATIM Fri Nov 29 00:40:08 2013
OBNUC 1H
EXMOD NON
OBFRQ 395.75 MHz
OBSET 124.00 KHz
OBFIN 10277.00 Hz
POINT 16384
FREQU 7912.96 Hz
SCANS 8
ACQTM 2.0705 sec
PD 4.9290 sec
PW1 7.20 usec
IRNUC 1H
CTEMP 23.6 c
SLVNT CDCL3
EXREF 0.00 ppm
BF 0.10 Hz
RGAIN 12
3.00
2.19
2.30
7.41
3.36
PPM
11.0 10.0 9.0 8.0 7.0 6.0 5.0 4.0 3.0 2.0 1.0 0.0
7.274
3.664 2.323 2.308 2.285 1.636 1.619 1.601 1.351 1.332 1.314 1.298 1.291 1.265 1.236 0.900 0.882 0.883 0.866 0.000

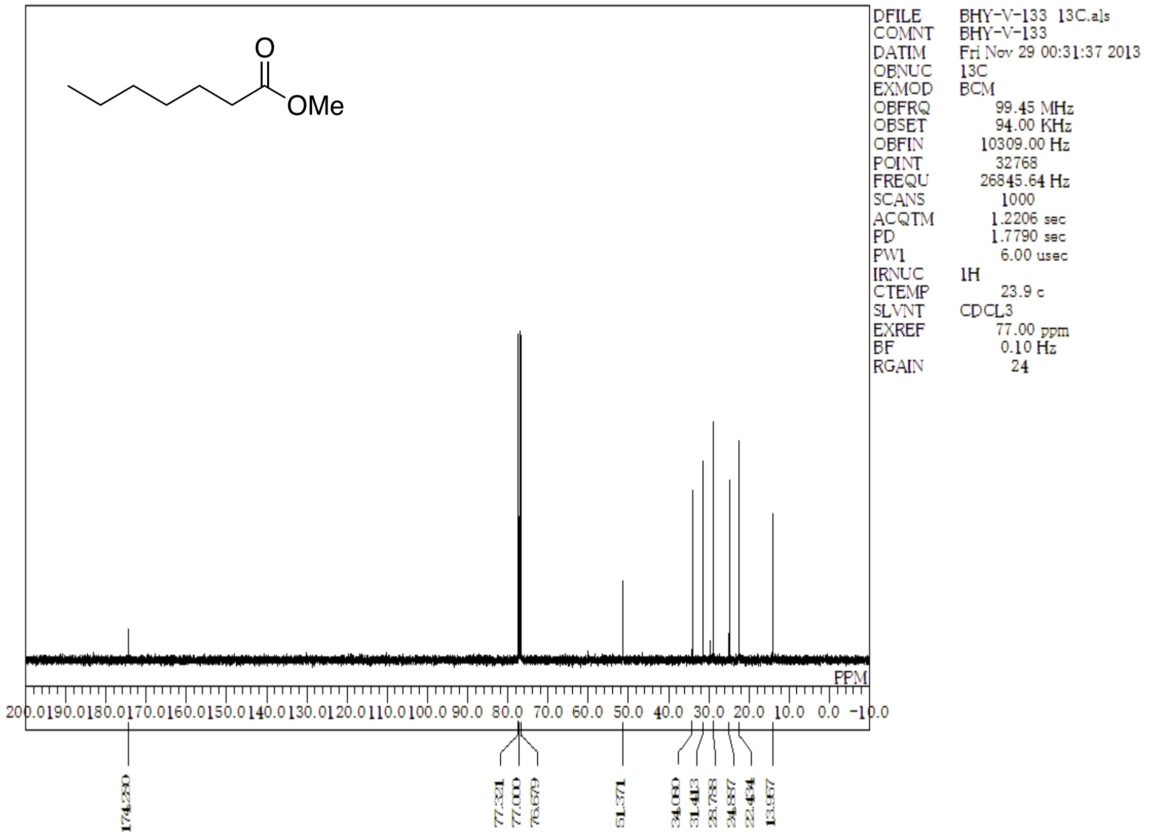
O
OMe
DFILE BHY-V-133 13C.als
COMNT BHY-V-133
DATIM Fri Nov 29 00:31:37 2013
OBNUC 13C
EXMOD BCM
OBFRQ 99.45 MHz
OBSET 94.00 KHz
OBFIN 10309.00 Hz
POINT 32768
FREQU 26845.64 Hz
SCANS 1000
ACQTM 1.2206 sec
PD 1.7790 sec
PW1 6.00 usec
IRNUC 1H
CTEMP 23.9 c
SLVNT CDCL3
EXREF 77.00 ppm
BF 0.10 Hz
RGAIN 24
PPM
200.0 190.0 180.0 170.0 160.0 150.0 140.0 130.0 120.0 110.0 100.0 90.0 80.0 70.0 60.0 50.0 40.0 30.0 20.0 10.0 0.0 −10.0
174.230
77.321 77.000 76.679
51.371
34.080 31.413 28.788 24.887 22.434 13.967

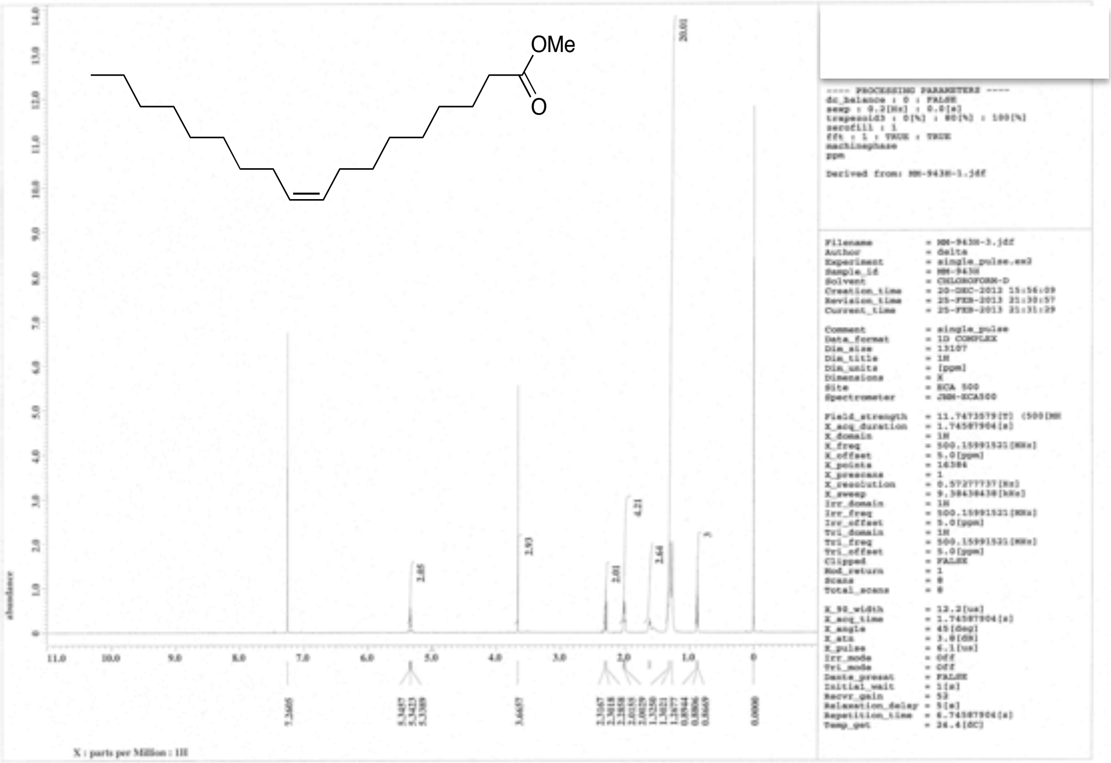
OMe
O
Derived from: MM-943H-1.jdf
X : parts per Million : 1H
abundance
7.2605
3.6657
0.0000

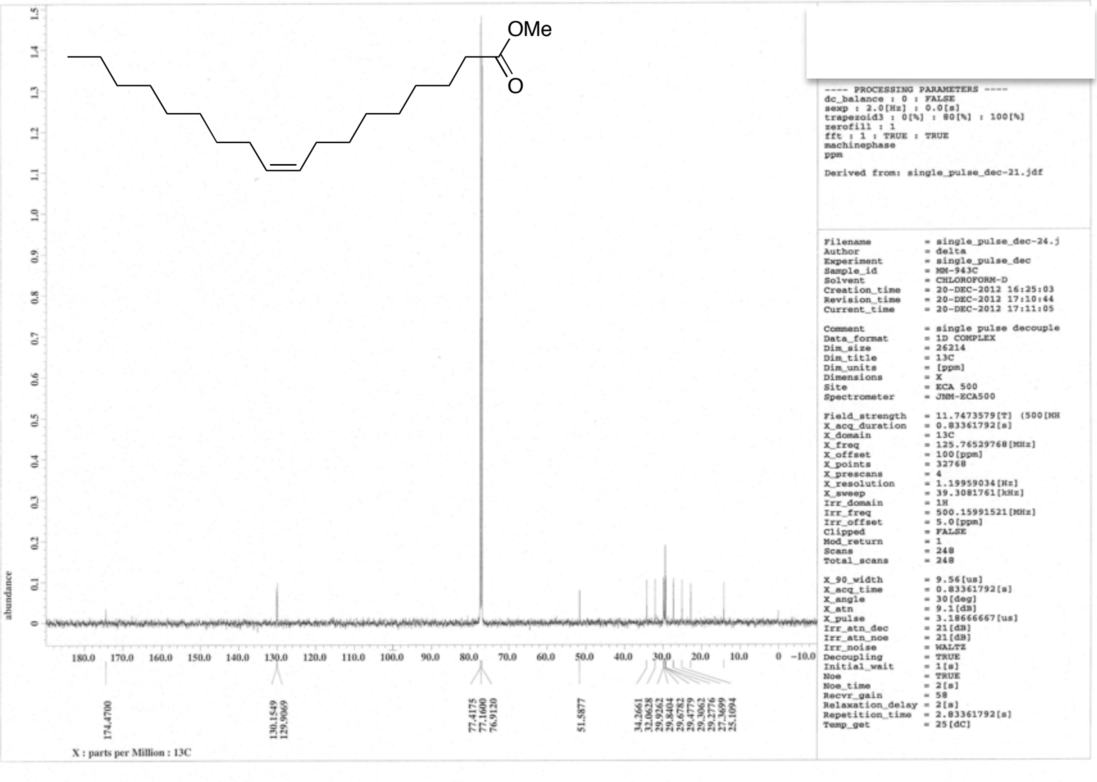
OMe
O
---- PROCESSING PARAMETERS ----
dc_balance : 0 : FALSE
sexp : 2.0[Hz] : 0.0[s]
trapezoid3 : 0[%] : 80[%] : 100[%]
zerofill : 1
fft : 1 : TRUE : TRUE
machinephase
ppm
Derived from: single_pulse_dec-21.jdf
Filename = single_pulse_dec-24.j
Author = delta
Experiment = single_pulse_dec
Sample_id = MM-943C
Solvent = CHLOROFORM-D
Creation_time = 20-DEC-2012 16:25:03
Revision_time = 20-DEC-2012 17:10:44
Current_time = 20-DEC-2012 17:11:05
Comment = single pulse decouple
Data_format = 1D COMPLEX
Dim_size = 26214
Dim_title = 13C
Dim_units = [ppm]
Dimensions = X
Site = ECA 500
Spectrometer = JNM-ECA500
Field_strength = 11.7473579[T] (500[MH
X_acq_duration = 0.83361792[s]
X_domain = 13C
X_freq = 125.76529768[MHz]
X_offset = 100[ppm]
X_points = 32768
X_prescans = 4
X_resolution = 1.19959034[Hz]
X_sweep = 39.3081761[kHz]
Irr_domain = 1H
Irr_freq = 500.15991521[MHz]
Irr_offset = 5.0[ppm]
Clipped = FALSE
Mod_return = 1
Scans = 248
Total_scans = 248
X_90_width = 9.56[us]
X_acq_time = 0.83361792[s]
X_angle = 30[deg]
X_atn = 9.1[dB]
X_pulse = 3.18666667[us]
Irr_atn_dec = 21[dB]
Irr_atn_noe = 21[dB]
Irr_noise = WALTZ
Decoupling = TRUE
Initial_wait = 1[s]
Noe = TRUE
Noe_time = 2[s]
Recvr_gain = 58
Relaxation_delay = 2[s]
Repetition_time = 2.83361792[s]
Temp_get = 25[dC]
X : parts per Million : 13C
abundance
174.4700
130.1549
129.9069
77.4175
77.1600
76.9120
51.5877
34.2661
32.0628
29.9262
29.8404
29.6782
29.4779
29.3062
29.2776
27.3699
25.1094

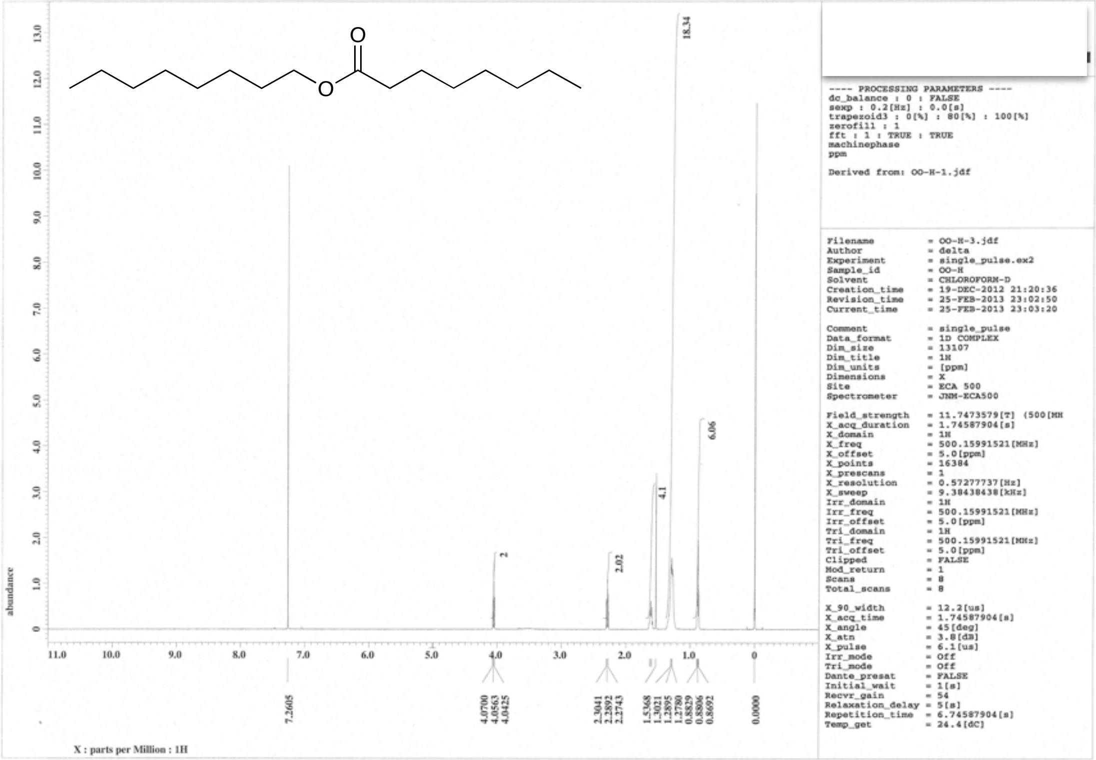
---- PROCESSING PARAMETERS ----
dc_balance : 0 : FALSE
sexp : 0.2[Hz] : 0.0[s]
trapezoid3 : 0[%] : 80[%] : 100[%]
zerofill : 1
fft : 1 : TRUE : TRUE
machinephase
ppm
Derived from: OO-H-1.jdf
Filename = OO-H-3.jdf
Author = delta
Experiment = single_pulse.ex2
Sample_id = OO-H
Solvent = CHLOROFORM-D
Creation_time = 19-DEC-2012 21:20:36
Revision_time = 25-FEB-2013 23:02:50
Current_time = 25-FEB-2013 23:03:20
Comment = single_pulse
Data_format = 1D COMPLEX
Dim_size = 13107
Dim_title = 1H
Dim_units = [ppm]
Dimensions = X
Site = ECA 500
Spectrometer = JNM-ECA500
Field_strength = 11.7473579[T] (500[MH
X_acq_duration = 1.74587904[s]
X_domain = 1H
X_freq = 500.15991521[MHz]
X_offset = 5.0[ppm]
X_points = 16384
X_prescans = 1
X_resolution = 0.57277737[Hz]
X_sweep = 9.38438438[kHz]
Irr_domain = 1H
Irr_freq = 500.15991521[MHz]
Irr_offset = 5.0[ppm]
Tri_domain = 1H
Tri_freq = 500.15991521[MHz]
Tri_offset = 5.0[ppm]
Clipped = FALSE
Mod_return = 1
Scans = 8
Total_scans = 8
X_90_width = 12.2[us]
X_acq_time = 1.74587904[s]
X_angle = 45[deg]
X_atn = 3.8[dB]
X_pulse = 6.1[us]
Irr_mode = Off
Tri_mode = Off
Dante_presat = FALSE
Initial_wait = 1[s]
Recvr_gain = 54
Relaxation_delay = 5[s]
Repetition_time = 6.74587904[s]
Temp_get = 24.4[dC]
O
O
18.34
6.06
4.1
2
2.02
7.2605
4.0700
4.0563
4.0425
2.3041
2.2892
2.2743
1.5368
1.3021
1.2895
1.2780
0.8829
0.8806
0.8692
0.0000
abundance
X : parts per Million : 1H

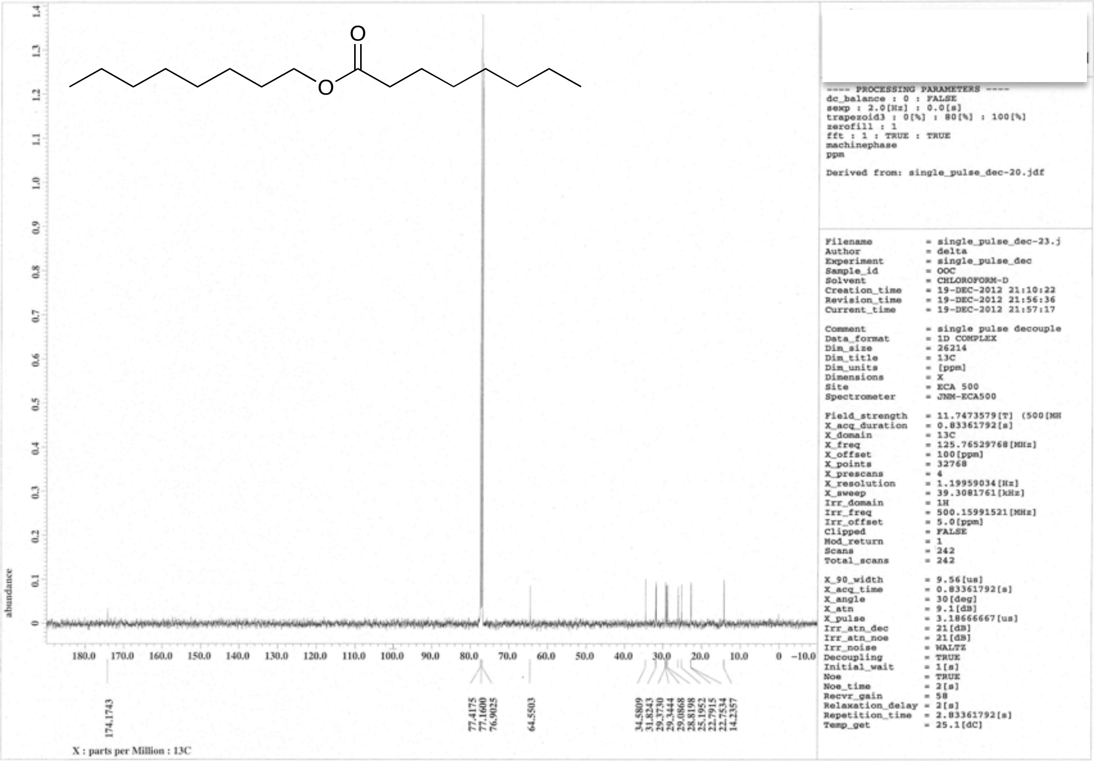
---- PROCESSING PARAMETERS ----
dc_balance : 0 : FALSE
sexp : 2.0[Hz] : 0.0[s]
trapezoid3 : 0[%] : 80[%] : 100[%]
zerofill : 1
fft : 1 : TRUE : TRUE
machinephase
ppm
Derived from: single_pulse_dec-20.jdf
Filename = single_pulse_dec-23.j
Author = delta
Experiment = single_pulse_dec
Sample_id = OOC
Solvent = CHLOROFORM-D
Creation_time = 19-DEC-2012 21:10:22
Revision_time = 19-DEC-2012 21:56:36
Current_time = 19-DEC-2012 21:57:17
Comment = single pulse decouple
Data_format = 1D COMPLEX
Dim_size = 26214
Dim_title = 13C
Dim_units = [ppm]
Dimensions = X
Site = ECA 500
Spectrometer = JNM-ECA500
Field_strength = 11.7473579[T] (500[MH
X_acq_duration = 0.83361792[s]
X_domain = 13C
X_freq = 125.76529768[MHz]
X_offset = 100[ppm]
X_points = 32768
X_prescans = 4
X_resolution = 1.19959034[Hz]
X_sweep = 39.3081761[kHz]
Irr_domain = 1H
Irr_freq = 500.15991521[MHz]
Irr_offset = 5.0[ppm]
Clipped = FALSE
Mod_return = 1
Scans = 242
Total_scans = 242
X_90_width = 9.56[us]
X_acq_time = 0.83361792[s]
X_angle = 30[deg]
X_atn = 9.1[dB]
X_pulse = 3.18666667[us]
Irr_atn_dec = 21[dB]
Irr_atn_noe = 21[dB]
Irr_noise = WALTZ
Decoupling = TRUE
Initial_wait = 1[s]
Noe = TRUE
Noe_time = 2[s]
Recvr_gain = 58
Relaxation_delay = 2[s]
Repetition_time = 2.83361792[s]
Temp_get = 25.1[dC]
O
O
174.1743
77.4175
77.1600
76.9025
64.5503
34.5809
31.8243
29.3730
29.3444
29.0868
28.8198
25.1952
22.7915
22.7534
14.2357
abundance
X : parts per Million : 13C

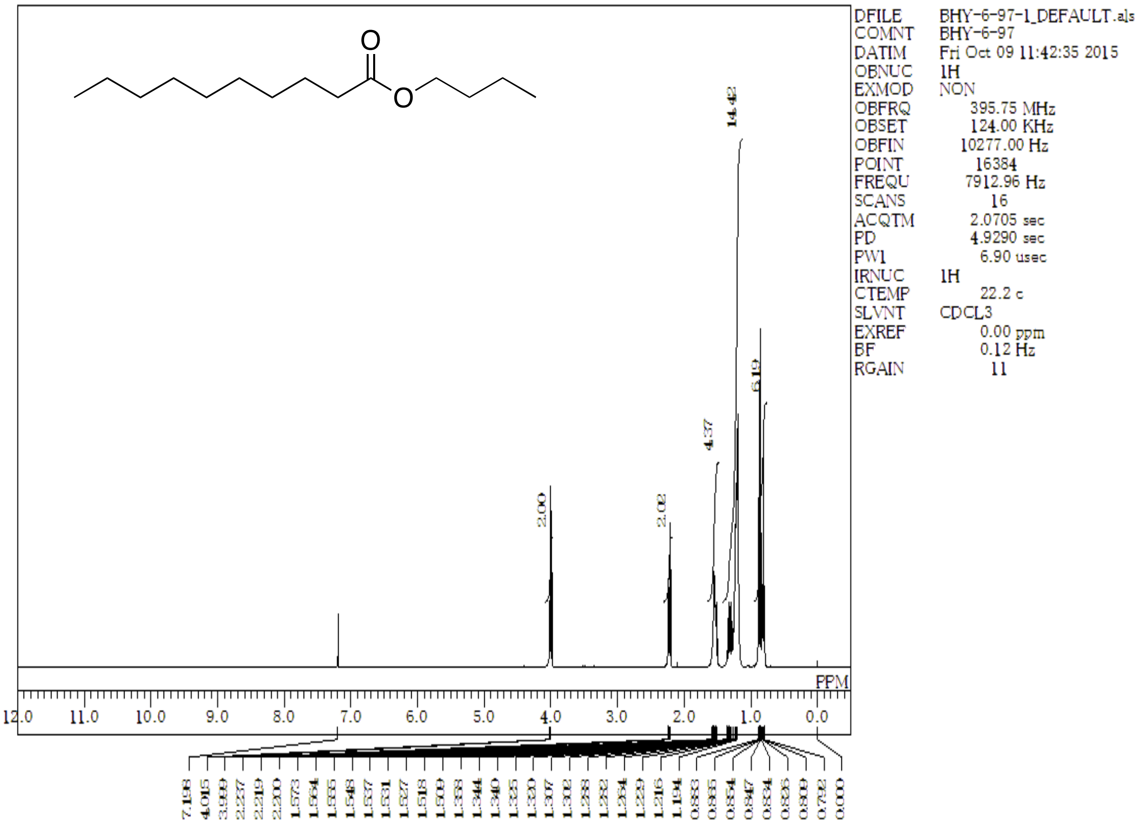
DFILE BHY-6-97-1_DEFAULT.als
COMNT BHY-6-97
DATIM Fri Oct 09 11:42:35 2015
OBNUC 1H
EXMOD NON
OBFRQ 395.75 MHz
OBSET 124.00 KHz
OBFIN 10277.00 Hz
POINT 16384
FREQU 7912.96 Hz
SCANS 16
ACQTM 2.0705 sec
PD 4.9290 sec
PW1 6.90 usec
IRNUC 1H
CTEMP 22.2 c
SLVNT CDCL3
EXREF 0.00 ppm
BF 0.12 Hz
RGAIN 11
PPM

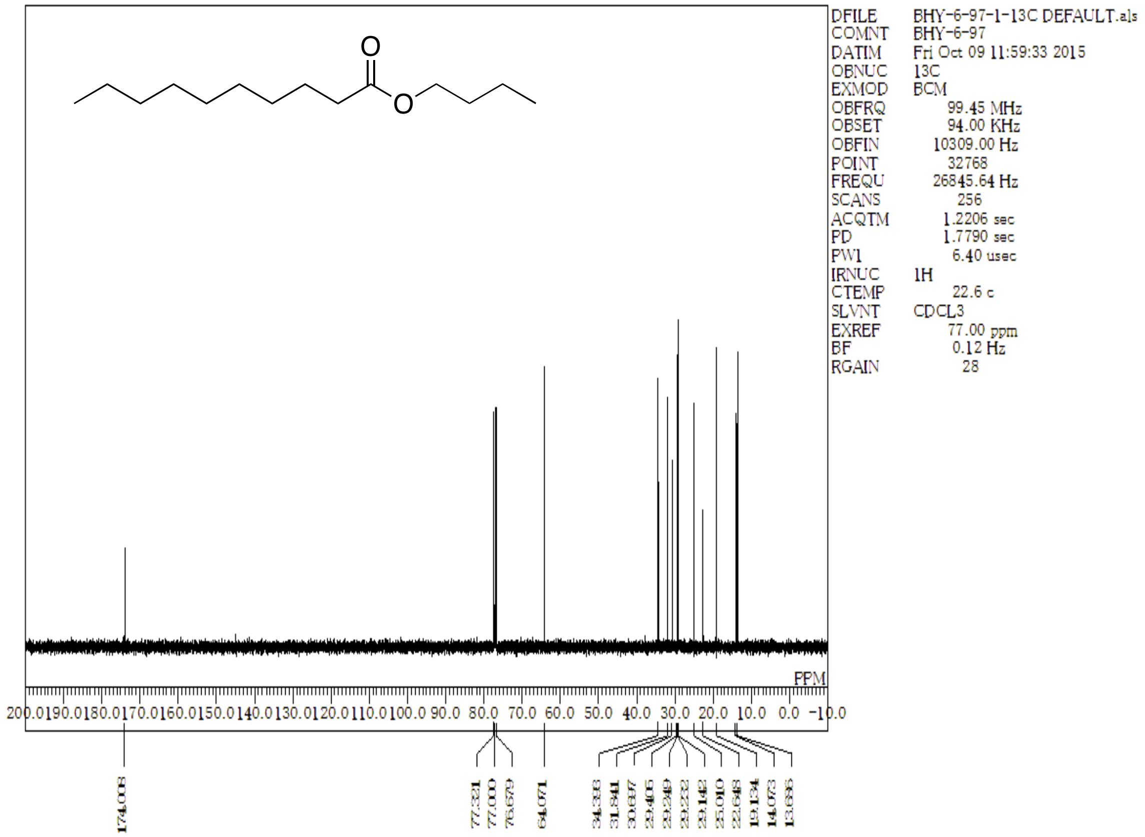
DFILE BHY-6-97-1-13C DEFAULT.als
COMNT BHY-6-97
DATIM Fri Oct 09 11:59:33 2015
OBNUC 13C
EXMOD BCM
OBFRQ 99.45 MHz
OBSET 94.00 KHz
OBFIN 10309.00 Hz
POINT 32768
FREQU 26845.64 Hz
SCANS 256
ACQTM 1.2206 sec
PD 1.7790 sec
PW1 6.40 usec
IRNUC 1H
CTEMP 22.6 c
SLVNT CDCL3
EXREF 77.00 ppm
BF 0.12 Hz
RGAIN 28
PPM
174.008
77.321
77.000
76.679
64.071
34.383
31.841
30.697
29.406
29.249
29.222
29.142
25.010
22.648
19.134
14.073
13.686

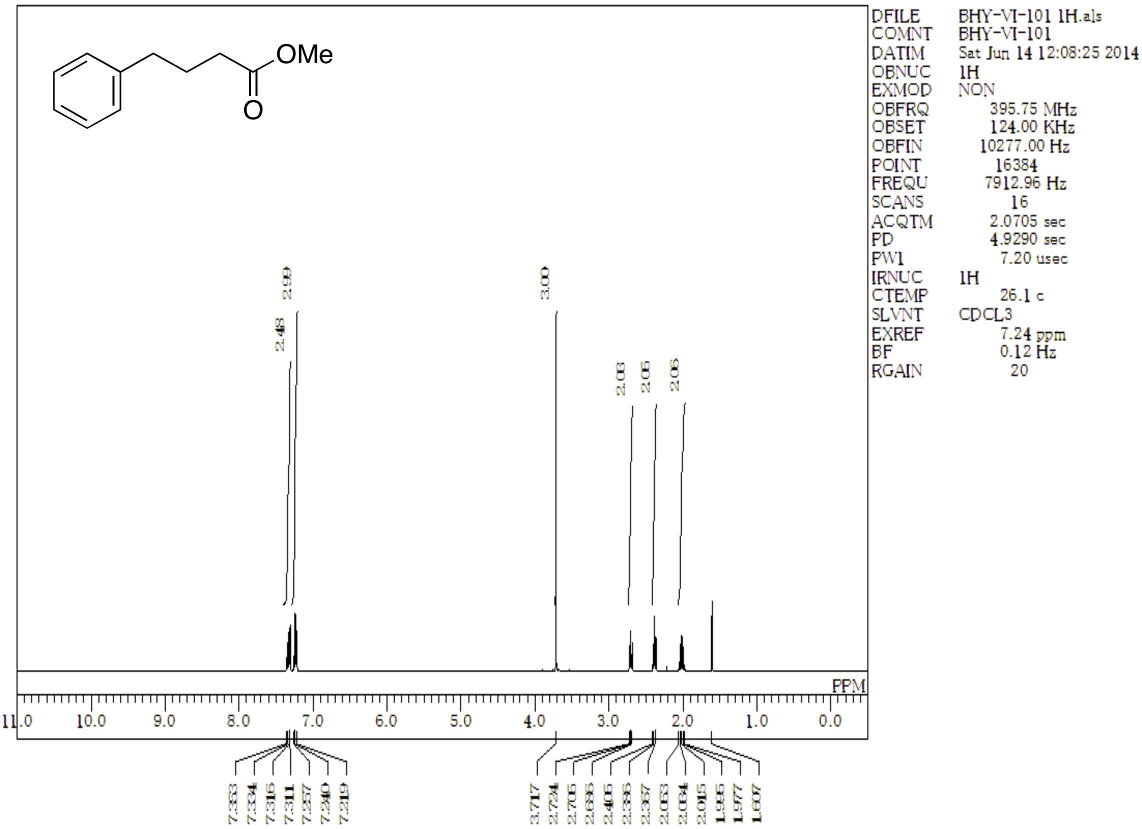
OMe
O
DFILE BHY-VI-101 1H.als
COMNT BHY-VI-101
DATIM Sat Jun 14 12:08:25 2014
OBNUC 1H
EXMOD NON
OBFRQ 395.75 MHz
OBSET 124.00 KHz
OBFIN 10277.00 Hz
POINT 16384
FREQU 7912.96 Hz
SCANS 16
ACQTM 2.0705 sec
PD 4.9290 sec
PW1 7.20 usec
IRNUC 1H
CTEMP 26.1 c
SLVNT CDCL3
EXREF 7.24 ppm
BF 0.12 Hz
RGAIN 20
PPM

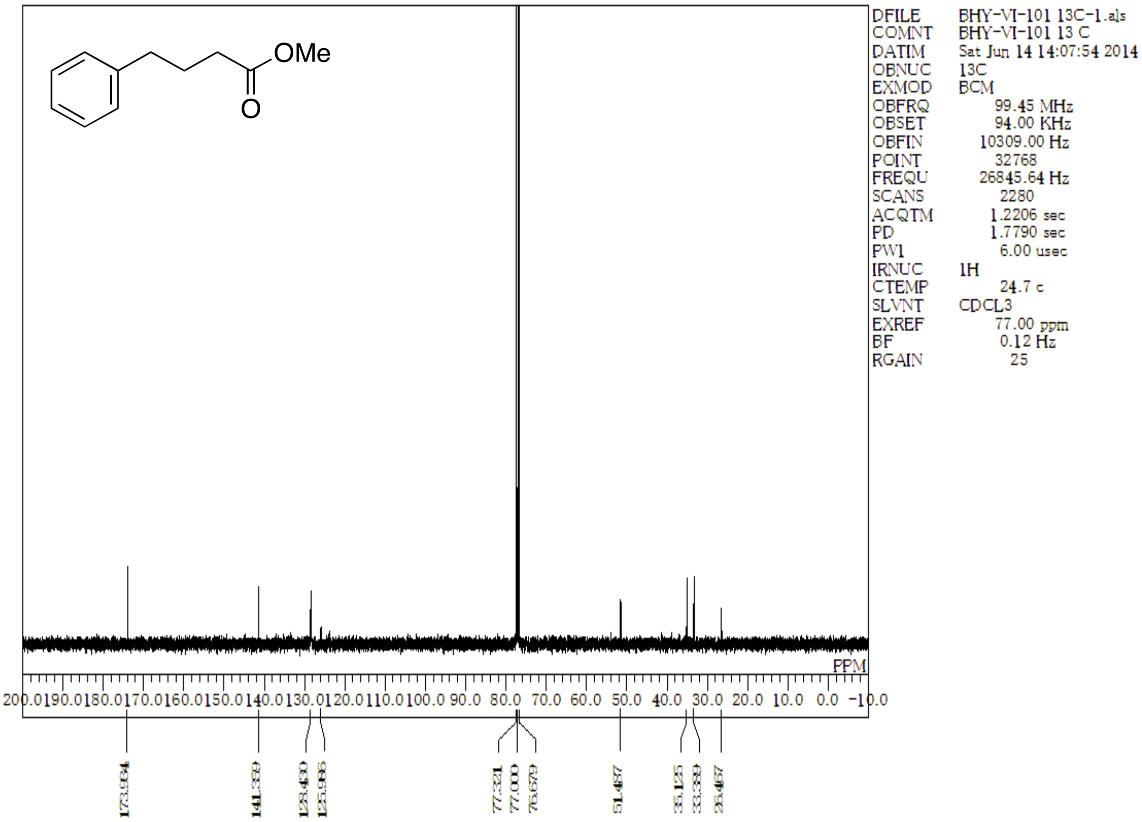
OMe
O
DFILE BHY-VI-101 13C-1.als
COMNT BHY-VI-101 13 C
DATIM Sat Jun 14 14:07:54 2014
OBNUC 13C
EXMOD BCM
OBFRQ 99.45 MHz
OBSET 94.00 KHz
OBFIN 10309.00 Hz
POINT 32768
FREQU 26845.64 Hz
SCANS 2280
ACQTM 1.2206 sec
PD 1.7790 sec
PW1 6.00 usec
IRNUC 1H
CTEMP 24.7 c
SLVNT CDCL3
EXREF 77.00 ppm
BF 0.12 Hz
RGAIN 25
PPM

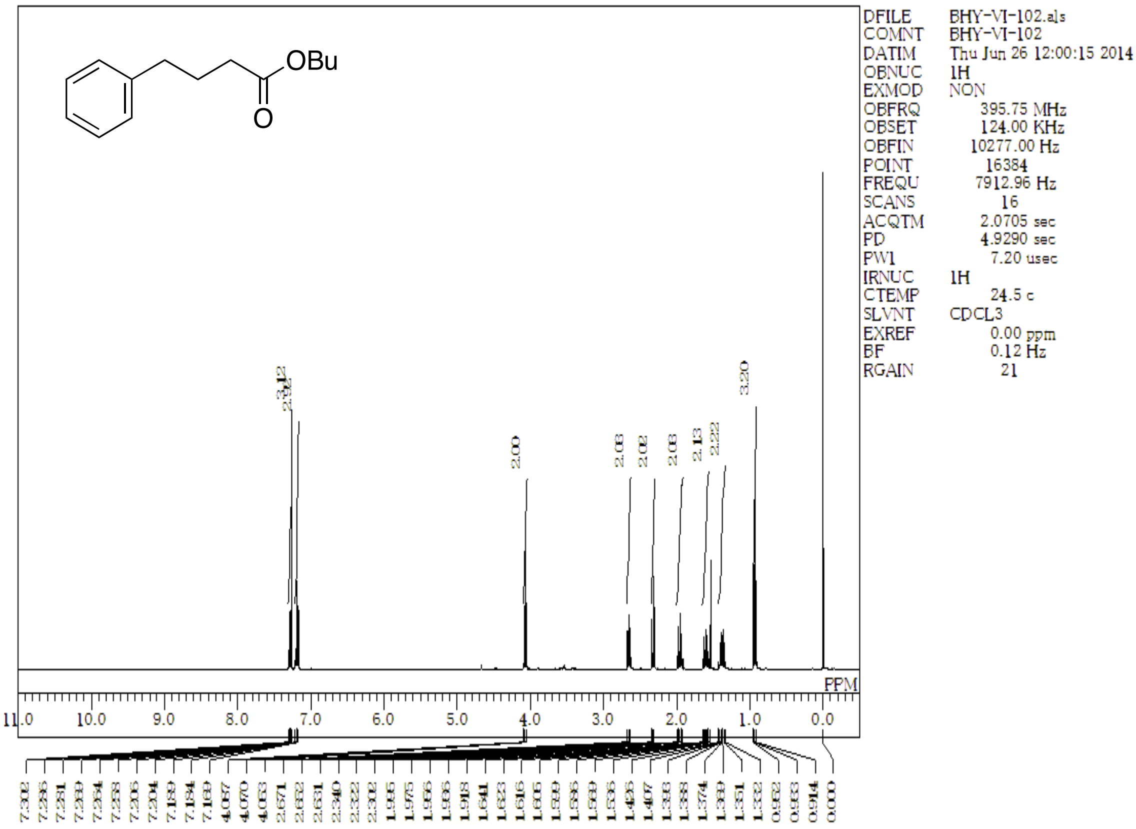

DFILE	BHY-VI-102.als
COMNT	BHY-VI-102
DATIM	Thu Jun 26 12:00:15 2014
OBNUC	1H
EXMOD	NON
OBFRQ	395.75 MHz
OBSET	124.00 KHz
OBFIN	10277.00 Hz
POINT	16384
FREQU	7912.96 Hz
SCANS	16
ACQTM	2.0705 sec
PD	4.9290 sec
PW1	7.20 usec
IRNUC	1H
CTEMP	24.5 c
SLVNT	CDCL3
EXREF	0.00 ppm
BF	0.12 Hz
RGAIN	21

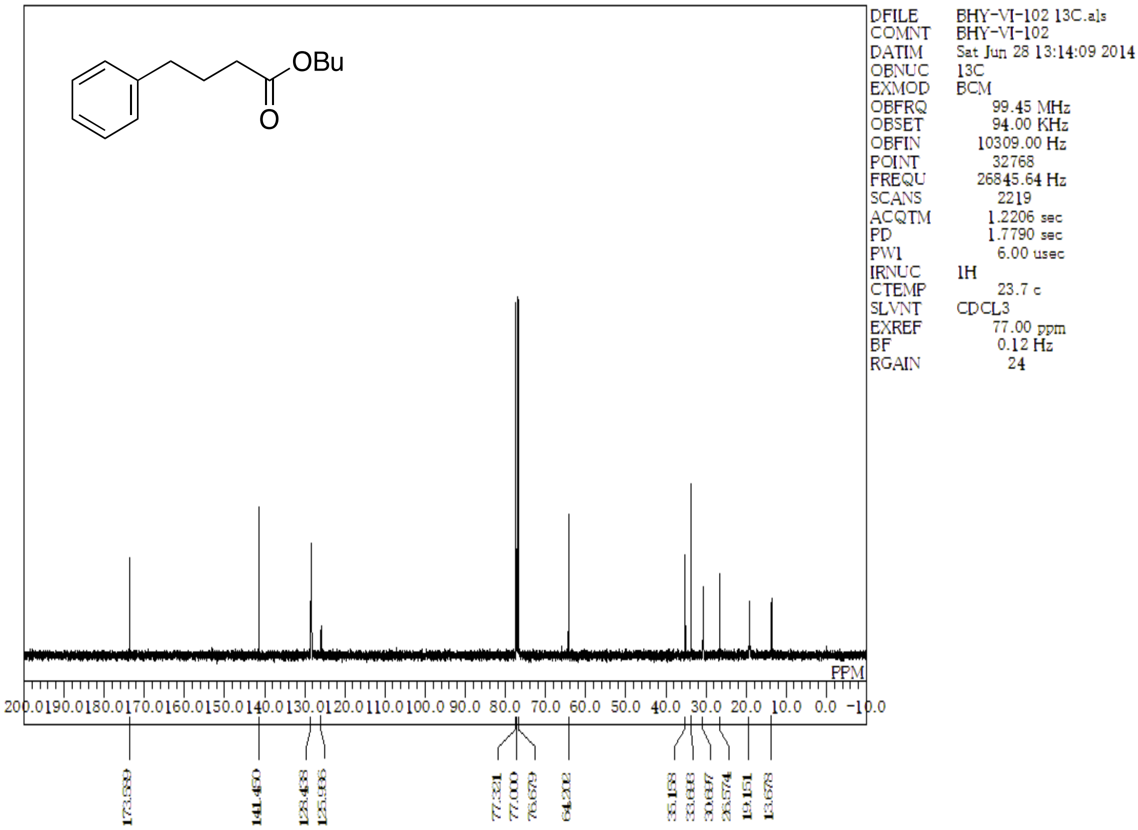

DFILE	BHY-VI-102 13C.als
COMNT	BHY-VI-102
DATIM	Sat Jun 28 13:14:09 2014
OBNUC	13C
EXMOD	BCM
OBFRQ	99.45 MHz
OBSET	94.00 KHz
OBFIN	10309.00 Hz
POINT	32768
FREQU	26845.64 Hz
SCANS	2219
ACQTM	1.2206 sec
PD	1.7790 sec
PW1	6.00 usec
IRNUC	1H
CTEMP	23.7 c
SLVNT	CDCL3
EXREF	77.00 ppm
BF	0.12 Hz
RGAIN	24

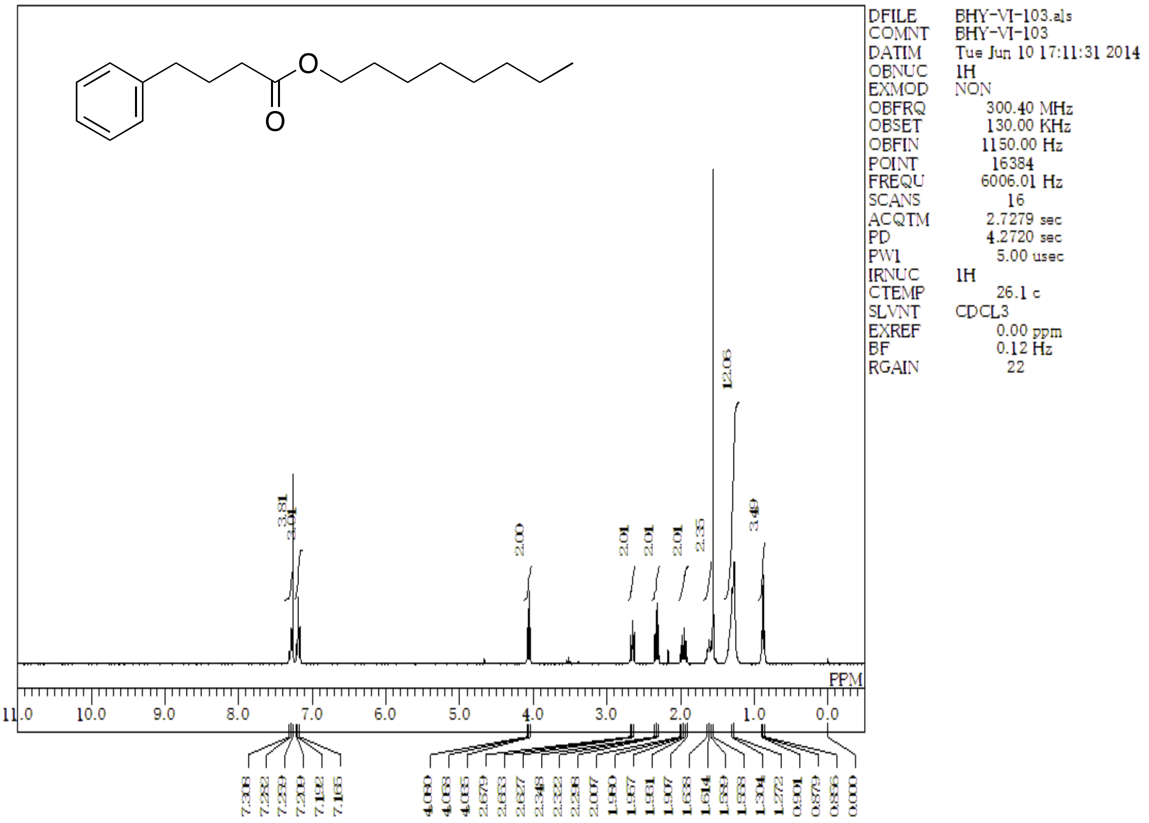
O
O
DFILE BHY-VI-103.als
COMNT BHY-VI-103
DATIM Tue Jun 10 17:11:31 2014
OBNUC 1H
EXMOD NON
OBFRQ 300.40 MHz
OBSET 130.00 KHz
OBFIN 1150.00 Hz
POINT 16384
FREQU 6006.01 Hz
SCANS 16
ACQTM 2.7279 sec
PD 4.2720 sec
PW1 5.00 usec
IRNUC 1H
CTEMP 26.1 c
SLVNT CDCL3
EXREF 0.00 ppm
BF 0.12 Hz
RGAIN 22
3.81
2.01
2.00
2.01
2.01
2.01
2.35
12.06
3.49
PPM
11.0 10.0 9.0 8.0 7.0 6.0 5.0 4.0 3.0 2.0 1.0 0.0
7.308 7.282 7.259 7.200 7.182 7.165
4.080 4.058 4.035 2.679 2.653 2.627 2.348 2.322 2.298 2.007 1.980 1.957 1.931 1.907 1.638 1.614 1.589 1.328 1.304 1.272 0.901 0.879 0.856 0.000

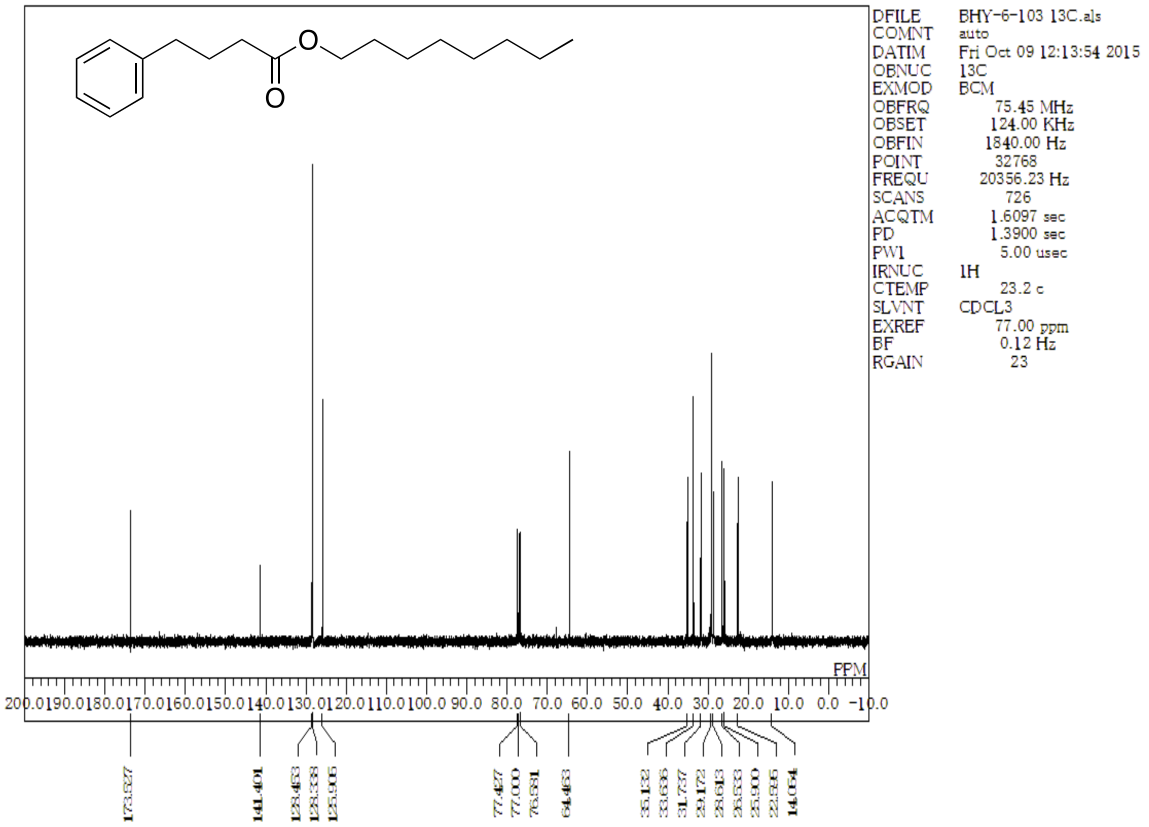
O
O
DFILE BHY-6-103 13C.als
COMNT auto
DATIM Fri Oct 09 12:13:54 2015
OBNUC 13C
EXMOD BCM
OBFRQ 75.45 MHz
OBSET 124.00 KHz
OBFIN 1840.00 Hz
POINT 32768
FREQU 20356.23 Hz
SCANS 726
ACQTM 1.6097 sec
PD 1.3900 sec
PW1 5.00 usec
IRNUC 1H
CTEMP 23.2 c
SLVNT CDCL3
EXREF 77.00 ppm
BF 0.12 Hz
RGAIN 23
PPM
200.0 190.0 180.0 170.0 160.0 150.0 140.0 130.0 120.0 110.0 100.0 90.0 80.0 70.0 60.0 50.0 40.0 30.0 20.0 10.0 0.0 -10.0
173.527
141.401
128.453 128.338 125.905
77.427 77.000 76.581
64.463
35.132 33.636 31.737 29.172 28.613 26.533 25.900 22.585 14.064
